# SLC2A3‐Mediated Lactate Metabolism Promotes Lung Cancer Bone Metastasis by Modulating P53 Lactylation and Immune Evasion

**DOI:** 10.1002/advs.202516622

**Published:** 2026-02-04

**Authors:** Yi Ding, Yuying Tian, Wenjie Ren, Xianglin Hu, Mengjuan Li, Bei Liu, Chen chen, Yunhan Lu, Lei Li, Wangjun Yan, Kun Li

**Affiliations:** ^1^ Tongde Hospital of Zhejiang Province Affiliated to Zhejiang Chinese Medical University (Tongde Hospital of Zhejiang Province) Hangzhou China; ^2^ Jiangong Hospital Clinical Research Center East China Normal University Shanghai China; ^3^ Shanghai Key Laboratory of Regulatory Biology School of Life Sciences East China Normal University Shanghai China; ^4^ Department of Musculoskeletal Oncology Fudan University Shanghai Cancer Center Shanghai China; ^5^ Ningxia Normal University Guyuan China; ^6^ Chongqing Key Laboratory of Precision Optics Chongqing Institute of East China Normal University Chongqing China

**Keywords:** glycolysis, lactylation, lung cancer bone metastasis, PD‐1, SLC2A3

## Abstract

Bone metastasis is a devastating consequence of lung cancer. However, the key metabolic factors that determine the risk of bone metastasis remain unclear. Here, we show that glucose transporter type 3 (SLC2A3) is notably overexpressed by lung cancer bone metastatic cells and tissues, as a facilitator of lung cancer bone metastasis. Additionally, SLC2A3 promotes glucose metabolism, which promotes tumor cell proliferation and metastasis via lactate‐mediated p53 lactylation. Within the tumor microenvironment, cancer cells serve as the primary source of secreted lactate, which induces protumor bone metastasis via osteoclast differentiation and suppresses the antitumor activity of CD8^+^ T cells. Subsequently, we developed Paris saponin VII, a SLC2A3 inhibitor that effectively suppressed bone metastasis in lung cancer bone metastasis mouse models and patient organoids. Notably, either inhibition of SLC2A3 or lactate limitation improved the tumor response and increased the sensitivity of lung cancer bone metastases to PD‐1 treatment. Collectively, our findings highlight that targeting SLC2A3‐mediated lactate metabolism, either alone or in combination with PD‐1 inhibition, is a potential strategy for treating lung cancer bone metastasis.

## Graphical abstract

SLC2A3 derived lactate promotes metastasis through p53 lactylation at K120 and osteoclast differentiation. Pharmacological inhibition of SLC2A3 upregulates PD‐1 expression on CD8^+^ T cells via lactate induced p53 lactylation, which modulates immune evasion.

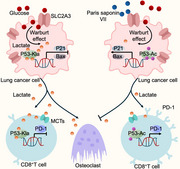

## 1 Introduction

Lung cancer is the leading cause of cancer‐related death worldwide [1]. Because of the complex etiology and insidious early symptoms of lung cancer, more than 70% of patients are diagnosed with metastatic stage IV disease at the time of presentation, leaving very limited treatment options; thus, the 5‐year survival rate is less than 4% [2, 3, 4]. The distant metastasis of lung cancer cells is the primary determinant of a poor lung cancer prognosis. Bone is the predominant target site for the distant metastasis of lung cancer. Up to 39% of patients with lung adenocarcinoma (LUAD) develop bone metastasis, with a median overall survival (OS) of only 5 months [5]. Even worse, bone metastasis, a frequent event in patients with advanced‐stage malignancies, often causes skeletal‐related events (SREs), such as pain, fractures, and hypercalcemia, greatly reduces quality of life and predicts a poor prognosis [6, 7]. Therefore, elucidating the mechanism of lung‐to‐bone metastasis to support the search for new targeted drugs and combination therapies for the treatment of lung cancer bone metastasis is highly clinically important.

Lactate (LA) secretion, a classic metabolic hallmark of cancer, is a consequence of the Warburg effect. This phenomenon exemplifies the metabolic reprogramming of cancer cells to prioritize glycolysis for energy generation, even in oxygen‐replete environments, which drives LA accumulation [8, 9, 10, 11]. In addition to simply supplying energy, abnormal LA metabolism drives lung cancer progression, such as by counteracting oxidative stress, suppressing antitumor immunity, and mediating therapeutic resistance [12, 13, 14]. In addition, LA‐induced lactylation, as a posttranslational modification, enhances the interaction between metabolic states and epigenetic regulation, accelerating tumor initiation, progression, and the development of drug resistance [15, 16, 17]. Research on NSCLC has shown that histone lysine lactylation (Kla) drives the downregulation of glycolytic enzymes and the upregulation of TCA cycle enzymes, driving metabolic dysregulation in tumor cells [18]. The functional importance of LA and LA‐induced lactylation in tumorigenesis and progression has been further supported by the development and application of LA and lactylation inhibitors, including AT‐101 (gossypol) and its derivatives, such as FX‐11 [19, 20, 21, 22, 23, 24], and the anti‐APOC2K70‐lac antibody [25]. Moreover, LA promotes the formation of a metastatic niche in bone metastases of colorectal cancer [26]. Therefore, in‐depth studies into the functions and roles of tumor metabolites, such as LA, in cancer progression and their impact on the tumor microenvironment (TME) are urgently needed.

Solute carrier family 2 member 3 (SLC2A3, also known as glucose transporter type 3 or GLUT3), a member of the solute carrier 2A (SLC2A) family of glucose transporters, contains 12 transmembrane domains and catalyzes the facilitative diffusion of D‐glucose [27, 28]. SLC2A3 has been reported to promote the progression of multiple cancers, including gastric cancer, head and neck squamous cell carcinoma, oral squamous cell carcinoma, colorectal cancer, testicular cancer and ovarian cancer [29, 30, 31, 32, 33, 34]. SLC2A3 regulates tumor immunity via glycolytic reprogramming [29]. A recent study also revealed that SLC2A3 promotes tumor progression through the LA‐promoted TGF‐β signaling pathway in oral squamous cell carcinoma [31], which suggests that targeting SLC2A3‐mediated LA metabolic reprogramming could be a potential therapeutic strategy for lung cancer bone metastasis.

In this study, we investigated the role and mechanisms of SLC2A3‐mediated LA metabolism in lung cancer bone metastasis. Our findings revealed that SLC2A3 plays a vital role in lung cancer bone metastasis. Altered glucose utilization through SLC2A3 facilitates increased p53 lactylation in cells, a molecular trait that promotes the tumor‐specific transcriptome. Cancer cell‐secreted LA maintains osteoclast differentiation and suppresses CD8^+^ T cell antitumor activity. Collectively, these findings reveal that SLC2A3‐ mediated LA metabolism is a key driver of lung cancer bone metastasis and highlight that LA acts as a metabolic checkpoint for controlling immune responses in the TME.

## 2 Results

### 2.1 SLC2A3 Overexpression Contributes to Lung Cancer Bone Metastasis

**FIGURE 1:**
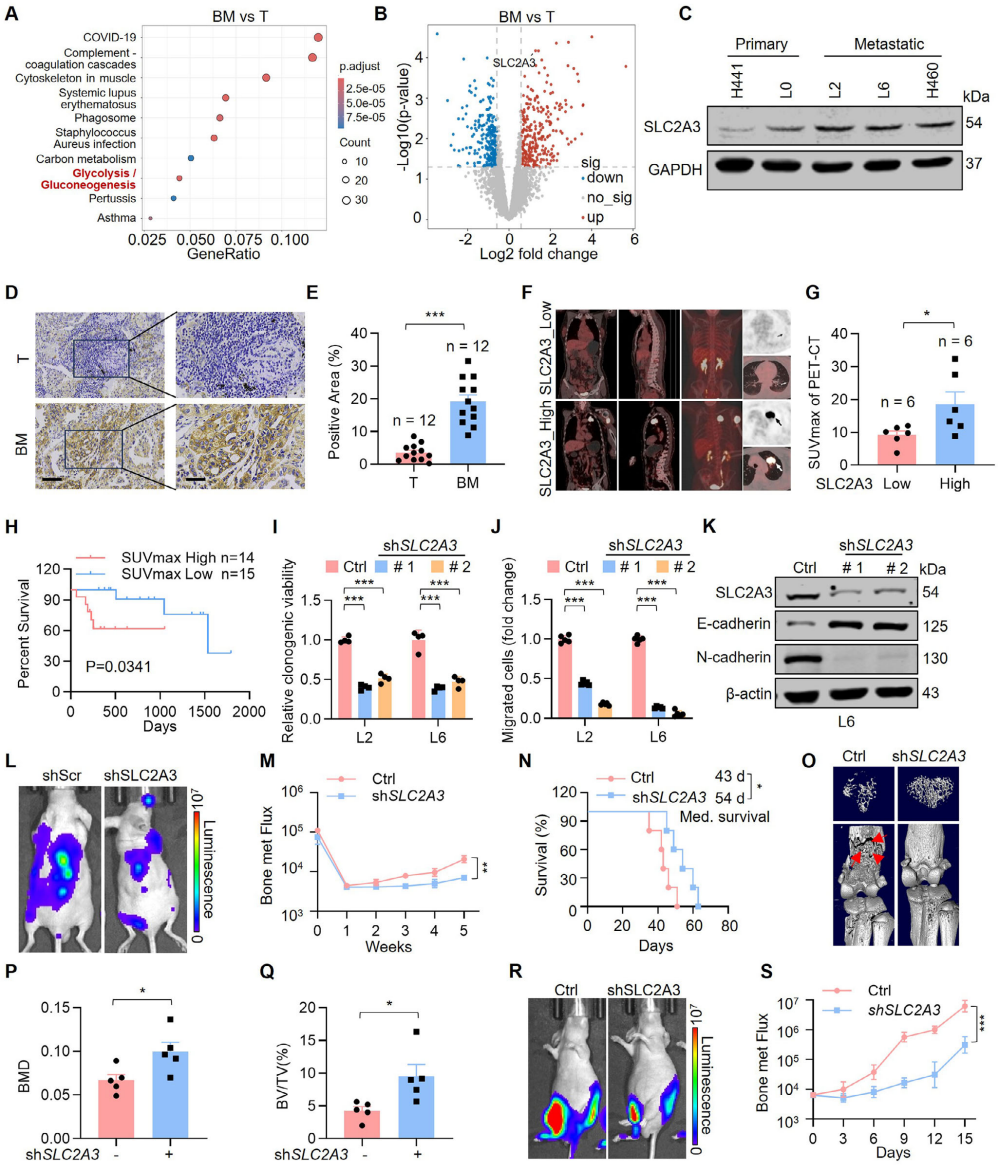
SLC2A3 promotes bone metastasis of lung cancer. (A) Pathway enrichment analysis reveals increased activation of the glycolysis/gluconeogenesis signaling pathway in lung cancer bone metastatic tumors (*n* = 6) compared to primary tumor tissues (*n* = 6). (B) Volcano plot shows markedly expressed proteins (raw *p* < 0.05, fold change < 0.67 or fold change > 1.5) between the tumor tissues of bone metastatic lesions and primary tumors in lung cancer patients. (*n* = 6). (C) Western blot analysis of SLC2A3 expression in primary and metastatic lung cancer cells. (D) Representative IHC images of SLC2A3 expression in primary lung cancer tissues and bone metastatic tumor tissues. Right: A higher magnification of sections. Scale bars: 100 µm (left), 50 µm (right). (E) SLC2A3 staining scores are shown. Each value represents mean ± SEM (*n* = 12), ^***^ *p* < 0.001. *p* value was measured by unpaired two‐tailed Student's *t* test. (F) Lung cancer bone metastasis patients were intravenously injected with ^18^F‐FDG in a fasting state, rested for 60 min, and then underwent Positron Emission Tomography‐Computed Tomography (PET‐CT) imaging. PET‐CT radiological images show the lung cancer metastasis status of patients with different SLC2A3 expressions. (G) Correlation analysis between the maximum ^18^F‐FDG uptake value (SUV_max_) and SLC2A3 expression level in patients. Each value represents mean ± SEM (*n* = 6), ^*^ *p* < 0.05. *p* value was measured by unpaired two‐tailed Student's *t* test. (H) Kaplan‐Meier survival analysis comparing patient survival between maximum ^18^F‐FDG uptake value (SUV_max_) high (SUV_max_ > 10, *n* = 14) versus low (SUV_max_ ≤ 10, *n* = 15) value. ^*^ *p* = 0.0341 by log‐rank test. (I) Statistical analysis of the colony formation of metastatic cells after knocking down SLC2A3. The relative clonogenic viability was normalized to vehicle‐treated control. Each value represents mean ± SEM (*n* = 4), ^***^ *p* < 0.001; *p* values were measured by one‐way ANOVA with Tukey's multiple comparison test. (J) Statistical analysis of the number of migrated cells after knocking down SLC2A3. The relative migration ability was normalized to vehicle‐treated control. Each value represents mean ± SEM (*n* = 5). ^***^ *p* < 0.001; *p* values were measured by unpaired two‐tailed Student's *t* test. (K) Western blot analysis of EMT markers after knocking down SLC2A3. (L) Representative images of relative bioluminescence intensity (BLI) in BALB/c nude mice injected with L6 Ctrl (*n* = 5) or L6‐shSLC2A3 (*n* = 5) cells via intracardiac (i.c.) injection. IVIS images were captured once every three days post injection, the i.c. injection model was lasted for 5 weeks. (M) BLI of the two groups in (L). Two‐way ANOVA was used to compare groups, ^**^ *p* < 0.01. (N) Kaplan‐Meier survival analysis of the BALB/c nude mice after the indicated treatments (L6Ctrl: *n* = 5; L6shSLC2A3: *n* = 5). Med., median. ^*^ *p* < 0.05 by log‐rank test. (O) Representative micro‐CT images of the tibia and trabecular bone from the mice in (L). (P, Q) Bar graph shows the quantitative micro‐CT analysis of trabecular bone from the tibia. BMD, bone mineral density; BV/TV, bone volume total/volume. Error bars are mean ± SEM. ^*^ *p* < 0.05 using unpaired two‐tailed Student's *t*‐test. (R) Representative images of relative BLI in BALB/c nude mice injected with L6Ctrl (*n* = 5) or L6‐shSLC2A3 (*n* = 5) via intratibial injection. IVIS images were captured once every three days post injection, the intratibial injection model was lasted for 15 days. (S) BLI of the two groups in (R). BLI represents the tumor burden in the tibia bone of the mice. Two‐way ANOVA was used to compare groups, ^***^ *p* < 0.001.

To identify therapeutically viable protein targets for lung cancer bone metastasis, we performed tandem mass tag (TMT)‐labeled quantitative proteomics analysis of samples from patients with lung cancer bone metastasis (BM tumors) and primary lung cancer patients (primary tumors). Our analysis revealed a total of 4532 proteins; among them, 279 proteins were significantly downregulated, and 271 proteins were significantly upregulated, with a > 1.5‐fold higher change in BM tumors than in primary tumors. Kyoto Encyclopedia of Genes and Genomes (KEGG) analysis revealed that these differentially regulated proteins (14 downregulated and 7 upregulated) were enriched in pathways related to carbon metabolism and glycolysis/gluconeogenesis (Figure 1A). These results may indicate that glucose reprogramming is involved in lung cancer bone metastasis. We noted that the expression of metabolic proteins involved in glycolysis, such as the glucose transporter SLC2A3 (also known as GLUT3), was significantly enriched in BM tumors compared with primary tumors (Figure 1B). SLC2A3, a main classical transporter for glucose uptake, serves as both a viable therapeutic drug and a predictor of poor prognosis in tumors [35, 36, 37]. Therefore, we focused on SLC2A3 for subsequent investigations.

We then validated the proteomics data by detecting the protein level of SLC2A3 in metastatic lung cancer cells and tissues by western blotting. The protein level of SLC2A3 was significantly increased in metastatic lung cancer cells (Figure 1C) and bone metastatic tissues (Figure 1D,E) relative to the primary tumors. Next, we detected radiological characteristics and SLC2A3 expression levels in a PET cohort including 12 patients with lung cancer bone metastasis who had undergo ^18^F‐FDG PET/CT scanning. Our results revealed that the maximum ^18^F‐FDG uptake value (SUV_max_) of patients was positively correlated with the expression of SLC2A3 (Figure 1F,G; Figure S1A), the details of the data are in the Table S5, and a high SUV_max_ value was correlated with poor overall survival (Figure 1H), suggesting a potential positive correlation between SLC2A3 expression and patient survival.

To explore the function of SLC2A3 in the bone metastasis of lung cancer, we transfected either SLC2A3 shRNA or control shRNA into the luciferase‐labeled bone‐metastatic A549 cell sublines (L2 and L6). We first compared the proliferation and migration abilities of SLC2A3‐knockdown (KD) cells with those of control cells. Colony formation and transwell assays revealed that SLC2A3 knockdown significantly decreased the number of formed colonies (Figure 1I; Figure S1B) and migrating cells (Figure 1J; Figure S1C). EMT features associated with bone metastasis [38, 39] were observed in both sublines, and western blot analysis revealed that reduced SLC2A3 expression in metastatic cells markedly increased E‐cadherin levels while decreasing N‐cadherin levels (Figure 1K). Compared with the control cells, cells with SLC2A3 overexpression (OE) exhibited greatly increased cell proliferation (Figure S1D,E) and migration (Figure S1F,G). Importantly, overexpression of SLC2A3 in SLC2A3‐knockdown cells fully restored both proliferation(Figure S1H,I) and migration ability (Figure S1J,K), whereas overexpression of SLC2A1‐another glucose transporter known to play crucial roles in lung cancer progression [40] only weakly rescued the impaired colony formation (Figure S1L,M) and migratory capacity (Figure S1N,O). Western blot analysis showed that re‐expression of SLC2A3, but not SLC2A1, in SLC2A3‐knockdown cells effectively restored the upregulated levels of E‐cadherin (Figure S1P,Q). Together, these results indicate that SLC2A3 plays an essential role in regulating the key metastatic processes in lung cancer colony formation and cellular migration.

We further investigated whether SLC2A3‐KD suppresses lung cancer bone metastasis in vivo via intracardiac (i.c.) injection [41] of luciferase‐labeled control and SLC2A3‐KD L6 cells into nude mice. Compared with the control mice, the mice harboring SLC2A3‐KD cells presented substantially reduced metastatic burdens (Figure 1L,M) and prolonged overall survival (Figure 1N), suggesting that SLC2A3 is critical for lung cancer bone metastasis. Micro‐CT analysis revealed lower bone resorption in the tibias of shSLC2A3 mice than in those of control mice (Figure 1O); the decrease in bone resorption was indicated by increased bone mineral density, bone volume, and trabecular number (Figure 1P,Q; Figure S1R). To further investigate the role of SLC2A3 in tumor progression and osteolysis, we intratibially injected control and SLC2A3‐KD cells into nude mice. Consistent with the intracardiac injection [42] results, SLC2A3‐KD significantly reduced the tumor growth rate compared with that in the control group (Figure 1R; Figure S1S). Collectively, these results suggest that SLC2A3 is a critical regulator for lung cancer bone metastasis.

### 2.2 SLC2A3‐Induced Glycolysis Promotion Is Associated with Lung Cancer Bone Metastasis and a Poor Prognosis

**FIGURE 2:**
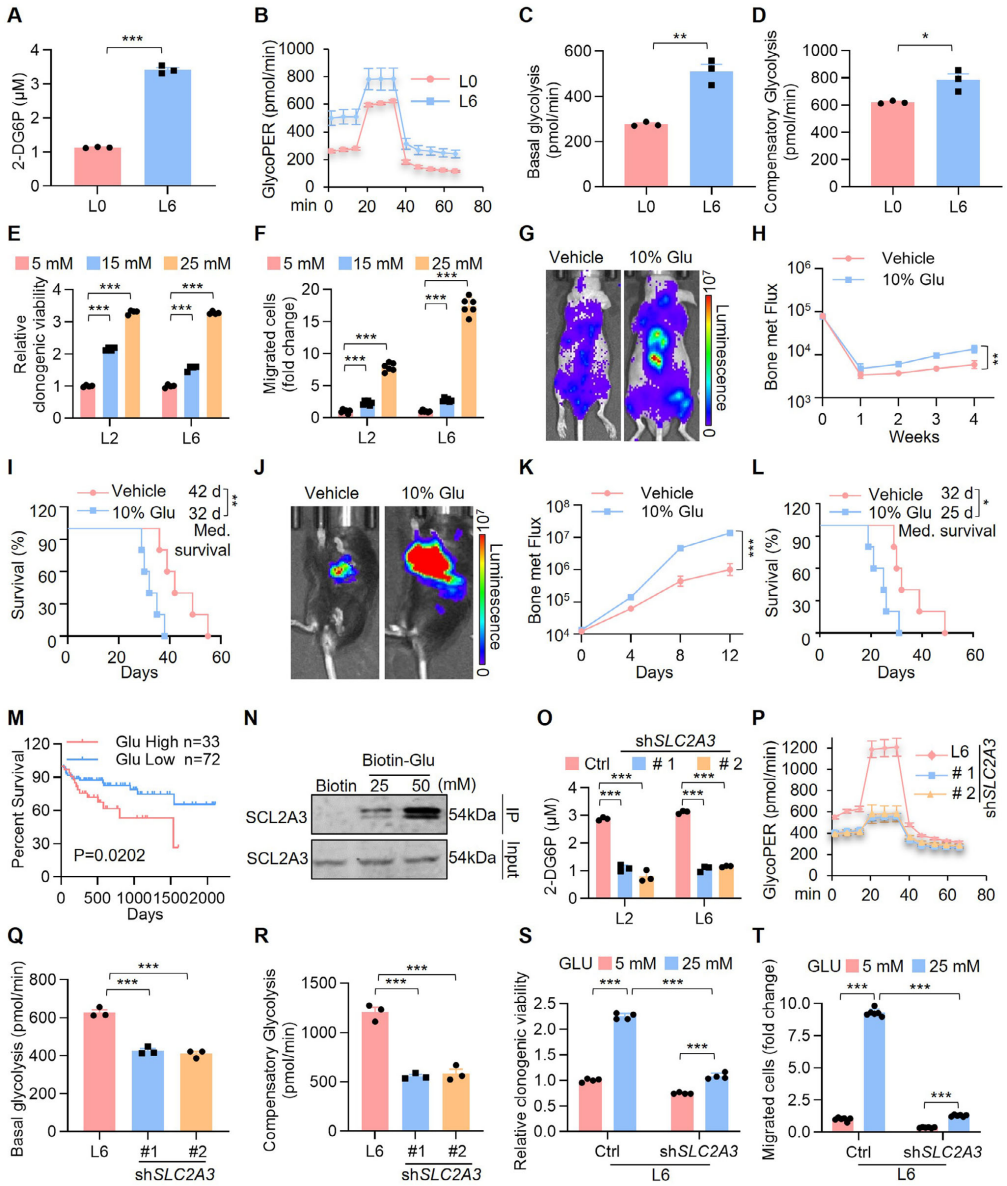
SLC2A3‐mediated glycolysis is associated with bone metastasis and poor prognosis in lung cancer. (A) Glucose uptake capacity assay in primary and metastatic lung cancer cells. Each value represents mean ± SEM (*n* = 3). ^***^ *p* < 0.001; *p* value was measured by unpaired two‐tailed Student's *t* test. (B–D) Glycolysis rate assay (*n* = 3) was conducted on primary and metastatic lung cancer cells. Statistical analysis was performed by Two‐way ANOVA test followed by Tukey post hoc test for (C) (basal glycolysis) and (D) (compensatory glycolysis), and by unpaired two‐tailed Student's *t* test. Each value represents mean ± StDev (*n* = 3), ^*^ *p* < 0.05; ^**^ *p* < 0.01. GlycoPER indicates glycolytic proton efflux rate. (E) The colony‐forming ability of metastatic cell lines after treatment with glucose at the concentration of 5 mM (Low), 15 mM (Medium) and 25 mM (High). The relative clonogenic viability was normalized to low glucose‐treated control. Each value represents mean ± SEM (*n* = 4). ^***^ *p* < 0.001; *p* values were measured by one‐way ANOVA with Tukey's multiple comparison test. (F) The migration ability of metastatic cell lines after treatment with glucose at the concentration of 5 mM (Low), 15 mM (Medium) and 25 mM (High). The relative migration viability was normalized to low glucose‐treated control. Each value represents mean ± SEM (*n* = 6). ^***^ *p* < 0.001; *p* values were measured by one‐way ANOVA with Tukey's multiple comparison test. (G) Representative images of relative BLI in BABL/c nude mice injected with L6 (*n* = 5) via i.c. injection. Mice in the experimental group were given glucose (10% glucose in water). IVIS images were captured once every three days post injection, the i.c. injection model was lasted for 4 weeks. (H) BLI of the two groups in (G). Two‐way ANOVA was used to compare groups, ^**^ *p* < 0.01. (I) Kaplan‐Meier survival analysis of the BALB/c nude mice after the indicated treatments (vehicle: *n* = 5; 10% glucose: *n* = 5). Med., median. ^**^ *p* <0.01 by log‐rank test. (J) Representative images of relative BLI in C57BL/6 mice injected with CMT167 (*n* = 5) via i.c. injection. Mice in the experimental group were given glucose (10% glucose in water). IVIS images were captured once every three days post injection, the i.c. injection model was lasted for 12 days. (K) BLI of the two groups in (J). Two‐way ANOVA was used to compare groups, ^***^ *p* < 0.001. (L) Kaplan‐Meier survival analysis of the BALB/c nude mice after the indicated treatments (vehicle: *n* = 5; 10% glucose: *n* = 5). Med., median. ^*^ *p* < 0.05 by log‐rank test. (M) Kaplan‐Meier survival analysis was used to compare patient survival, with patients divided into a glucose‐low group (3.9 mM/L < fasting blood glucose < 6.1 mM/L, *n* = 72) and a glucose‐high group (fasting blood glucose ≥ 6.1 mM/L, *n* = 33) based on normal fasting blood glucose levels as the criterion. ^*^ *p* = 0.0202 by log‐rank test. (N) L6 cells were lysed with CHAPS buffer, then treated with biotin or biotin‐glucose for 6 h. Pull‐down was performed using streptavidin agarose resin, followed by western blot analysis with antibodies against the indicated proteins. (O) Glucose uptake capacity assay in lung cancer metastatic cells after knocking down SLC2A3. Each value represents mean ± SEM (*n* = 3). ^***^ *p* < 0.001; *p* values were measured by one‐way ANOVA with Tukey's multiple comparison test. (P–R) Glycolysis rate assay (*n* = 3) was conducted on L6 after knocking down SLC2A3. Each value represents mean ± StDev (*n* = 3), ^***^ *p* < 0.001. (S) The colony‐forming ability of L6 cells after SLC2A3 knockdown and treatment with glucose at the concentration of 5 mM (Low), and 25 mM (High). The relative clonogenic viability was normalized to low glucose‐treated control. Each value represents mean ± SEM (*n* = 4). ^***^ *p* < 0.001; *p* values were measured by one‐way ANOVA with Tukey's multiple comparison test. Each value represents mean ± SEM (*n* = 4). ^***^ *p* < 0.001; *p* values were measured by unpaired two‐tailed Student's *t* test. (T) The migration ability of L6 cells after SLC2A3 knockdown and treatment with glucose at the concentration of 5 mM (Low), and 25 mM (High). The relative migration viability was normalized to low glucose‐treated control. Each value represents mean ± SEM (*n* = 6). ^***^ *p* < 0.001; *p* values were measured by one‐way ANOVA with Tukey's multiple comparison test.

To investigate the downstream pathways regulated by SLC2A3, we conducted metabolomics analysis in SLC2A3‐knockdown cells, which revealed a significant alteration in the glycolytic signaling pathway (Figure S2A). Given the glucose transporter SLC2A3 is involved in the first step of glycolysis and facilitates glucose entry into cells [29], we assessed alterations in glucose metabolism in lung cancer bone metastatic cells. The results revealed that bone metastatic cells exhibited both increased glucose uptake (Figure 2A) and elevated extracellular acidification rate (ECAR) (Figure 2B–D) compared to primary lung cancer cells. We also observed that patients with a greater tumor glucose uptake capacity, as indicated by a higher SUV_max_, tended to have a greater tumor metastasis burden (Figure 1F,G).

The increase in glycolysis begins with glucose addiction [43]; therefore, we examined the effect of a high‐glucose diet (HGD) on lung cancer bone metastasis. We first compared the cell proliferation and migration ability of dietary glucose with those of the controls. Colony formation and transwell assays revealed that dietary glucose significantly increased the number of formed colonies (Figure 2E; Figure S2B) and migrating cells (Figure 2F; Figure S2C). Next, BALB/c nude mice injected with luciferase‐labeled L6 cells into the left ventricle were treated with vehicle or 10% glucose water for 4 weeks, with tumor burden quantified weekly by bioluminescence imaging (BLI). Our BLI results revealed that glucose water significantly increased bone metastasis (Figure 2G,H), bone destruction (Figure S2D) and the number of osteolytic bone lesions (Figure S2E–G). Moreover, Kaplan–Meier survival tests indicated that dietary glucose significantly shortened metastasis‐free survival in the dietary glucose‐treated mice to a median of 32 days compared with the median of 42 days in the control mice (Figure 2I). In addition, we injected luciferase‐labeled CMT167 cells into immunocompetent C57BL/6 mice through left ventricular injection. Consistent with the BALB/c nude results, dietary glucose significantly increased the tumor growth rate and bone resorption, whereas the addition of glucose markedly decreased survival, with an added median survival time of 7 days (Figure 2J–L). We further explored the associations between glucose levels and survival outcomes using a retrospective clinical study of a cohort of lung cancer patients with localized bone metastasis. The group with high glucose levels had a worse survival rate than the group with low glucose levels did (Figure 2M). These results revealed that increased cellular glycolytic capacity facilitates lung cancer bone metastasis.

We next aimed to determine whether the effect of glycolysis on lung cancer bone metastasis was correlated with SLC2A3. We synthesized biotin‐labeled glucose, incubated it with cell lysate from L6 cells, and pulled down potential glucose‐interacting proteins with streptavidin beads, followed by immunoblot analysis. The results revealed that biotin‐glucose directly interacts with SLC2A3 (Figure 2N). Moreover, SLC2A3 inhibition impaired glucose uptake (Figure 2O) and glycolytic capacity (Figure 2P–R). We also observed that glucose supplementation stimulated cancer cell growth (Figure 2S; Figure S2H) and migration (Figure 2T; Figure S2I), and this effect was greatly diminished by SLC2A3 inhibition, suggesting that SLC2A3 is required for the glucose metabolic process. Thus, we conclude that SLC2A3‐mediated high‐glucose metabolic processes trigger the development of lung cancer bone metastasis.

### 2.3 SLC2A3 Mediates the Participation of Intracellular LA in p53 Lactylation at K120

**FIGURE 3:**
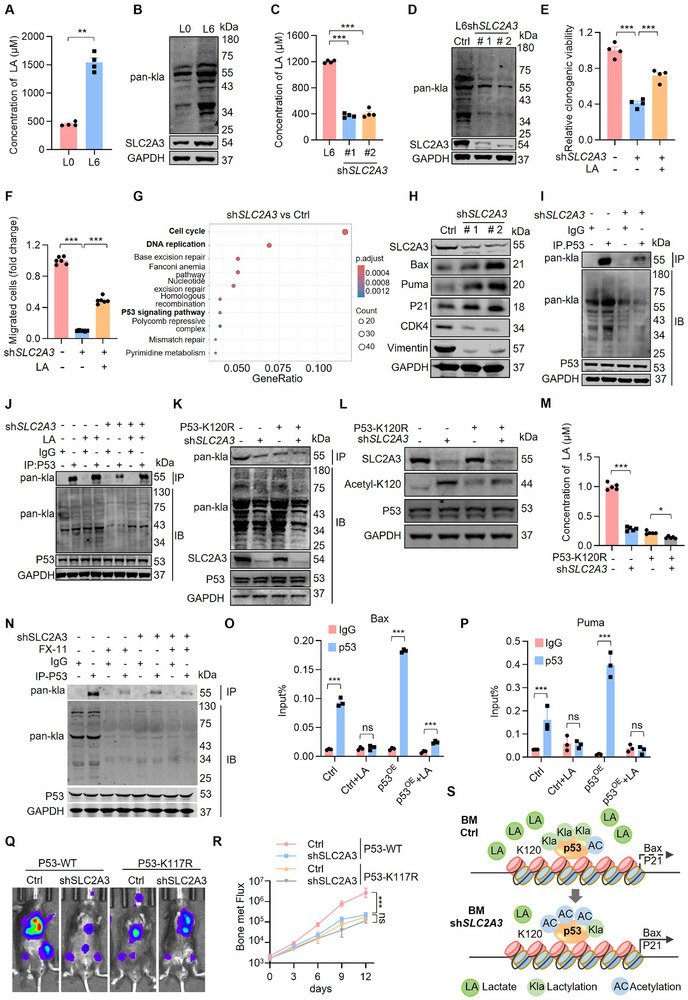
SLC2A3‐mediated intracellular LA accumulation promotes p53 lactylation at K120. (A) Bar graph shows the LA levels in primary and metastatic lung cancer cells. Each value represents mean ± SEM (*n* = 4). ^***^ *p* < 0.001; *p* value was measured by unpaired two‐tailed Student's *t* test. (B) Western blot analysis of pan‐lactylation levels in primary and metastatic lung cancer cells. (C) Bar graph shows the LA levels in L6 cells after knocking down SLC2A3. Each value represents mean ± SEM (*n* = 4). ^***^ *p* < 0.001; *p* values were measured by one‐way ANOVA with Tukey's multiple comparison test. (D) Western blot analysis of pan‐Kla levels in L6 cells after knocking down SLC2A3. (E) Statistical analysis of the colony formation results of L6 cells after knocking down SLC2A3 and treatment with or without LA (20 mM). The relative clonogenic viability was normalized to vehicle‐treated control. Each value represents mean ± SEM (*n* = 4). ^***^ *p* < 0.001; *p* values were measured by one‐way ANOVA with Tukey's multiple comparison test. (F) Statistical analysis of the number of migrated cells of L6 cells after knocking down SLC2A3 and treatment with or without LA (20 mM). The relative migration ability was normalized to vehicle‐treated control. Each value represents mean ± SEM (*n* = 6). ^***^ *p* < 0.001; *p* values were measured by one‐way ANOVA with Tukey's multiple comparison test. (G) Pathway enrichment analysis reveals the signaling pathways downregulated in L6 cells after SLC2A3 knockdown (*n* = 3). (H) Western blot analysis of p53 signaling pathway markers in L6 cells after knocking down SLC2A3. (I) L6‐Ctrl and L6‐shSLC2A3 cells were lysed with CHAPS lysis buffer. Immunoprecipitations of cell lysates with anti‐p53 or anti‐IgG, followed by immunoblotting with antibodies against the indicated proteins. (J) L6Ctrl and L6shSLC2A3 cells treatment with or without LA (20 mM) were lysed with CHAPS lysis buffer. Immunoprecipitations of cell lysates with anti‐p53 or anti‐IgG, followed by immunoblotting with antibodies against the indicated proteins. (K) L6 cells after SLC2A3 knockdown and p53‐K120 mutant were lysed with CHAPS lysis buffer. Immunoprecipitations of cell lysates with anti‐HA agarose, followed by immunoblotting with antibodies against the indicated proteins. (L) Western blot analysis of p53 (acetyl‐K120) expression in L6 cells after SLC2A3 knockdown and p53‐K120 mutant. (M) Bar graph shows the lactate (LA) levels following SLC2A3 knockdown combined with LDHA inhibitor. (FX‐11, 8 µM) treatment in L6 cells. Each value represents mean ± SEM (*n* = 5). ^*^ *p* < 0.05, ^***^ *p* < 0.001; *p* values were measured by one‐way ANOVA with Tukey's multiple comparison test. (N) Western blot analysis of p53 lactylation following SLC2A3 knockdown combined with LDHA inhibitor (FX‐11, 8 µM) treatment in L6 cells. (O, P) Chromatin immunoprecipitation (ChIP)‑qPCR analysis of p53 enrichment at the *PUMA* and *BAX* promoter in L6 cells with or without exogenous LA (20 mM) treatment. The relative enrichment was normalized to the input control and presented as fold change over the untreated group. Each value represents mean ± SEM (n = 3). ^***^ *p* < 0.001; *p* value was measured by unpaired two‑tailed Student's *t* test. (Q, R) Representative bioluminescence images of mice at the endpoint after intracardiac injection with/without p53‑K117R knock‑in CMT167 or CMT167sh*SLC2A3* cells (*n* = 5). Two‐way ANOVA was used to compare groups, ^***^ *p* < 0.001. (S) The schematic diagram illustrates SLC2A3‐mediated lactylation of p53‐K120.

Lactylation is a posttranslational modification that represents the intersection of metabolism and epigenetics. This modification enhances the interaction between metabolic states and epigenetic regulation, accelerating tumor onset and metastasis [44, 45]. High LA levels in cancer cells lead to protein lactylation [46]. To determine the roles of LA and protein lactylation, we measured LA and lactylation level alterations in lung cancer bone metastatic cells. Compared with those in primary lung cancer cells, cellular LA production (Figure 3A) and pan‐lactylation (Figure 3B) were significantly promoted in bone metastatic cells. We wondered whether protein lactylation controlled lung cancer bone metastasis in an SLC2A3‐dependent manner. As shown in Figure 3C and Figure 3D, SLC2A3‐KD diminished cellular LA production and pan‐lactylation in lung cancer bone metastatic cells. We also found that tumor cells depleted of SLC2A3 presented markedly reduced colony formation (Figure 3E; Figure S3A) and cell migration (Figure 3F; Figure S3B), while the tumor‐suppressive effects were subsequently rescued by LA treatment. Both tumor cells and immune cells primarily uptake LA through monocarboxylate transporters MCT1 [46]. AZD3965 is an inhibitor of MCT1. Inhibition of MCT1, which blocks exogenous lactate uptake, still allowed SLC2A3 knockdown to effectively suppress lung cancer cell migration, indicating that cell migration is mediated by SLC2A3‐driven endogenous lactate rather than exogenous uptake (Figure S3C–E). These observations indicate that SLC2A3‐mediated lactylation upregulation contributes to the malignant behavior of lung cancer bone metastasis.

Lactylation alterations promote a malignant phenotype via multiple molecular mechanisms [46], and we used mass spectrometry (MS) to characterize SLC2A3‐mediated signaling pathways. KEGG analysis of these differentially regulated proteins revealed enriched pathways related to the p53 signaling pathway and its downstream signaling pathways, including the cell cycle and DNA replication pathways (Figure 3G; Figure S3F). Western blot analysis also showed that SLC2A3 inhibition significantly increased the levels of the apoptosis‐related proteins (Puma and BAX) and the cell cycle related protein (p21), while decreasing the migration marker Vimentin (Figure 3H). Moreover, we measured the apoptotic capacity and found that SLC2A3 depletion increased the apoptosis of lung cancer bone metastasis cells (Figure S3G–I). Given that p53 lactylation in its DNA‐binding domain (DBD) can reduce its transcriptional activity and tumor‐suppressive role [47], we wondered whether SLC2A3‐mediated lactylation of p53 contributes to its transcription. As shown in Figure 3I and Figure 3J, SLC2A3 depletion sharply decreased p53 lactylation, and the decrease in p53 lactylation was abolished by LA treatment.

Lysine 120 (K120) within the DNA‐binding domain of p53 is evolutionarily conserved across all species, and K120 appears to be one of the key p53 residues that modulate its essential functions [48]. K120 of p53 is modified not only by lactylation but also by acetylation, which increases p53 transcriptional activity of apoptosis‐related genes (*PUMA*, *BAX*) and other p53 target genes (*p21*, *MDM2*) [47, 49]. Moreover, the acetylation of K120 in p53 is often suppressed by its lactylation [47]. To evaluate whether these modifications are involved in the transcriptional activation of p53‐mediated apoptosis‐related genes (*PUMA*, *BAX*) and other p53 target genes (*p21*, *MDM2*), we generated a series of p53 mutants in which the lysine residues were replaced with arginine to block lactylation or acetylation. SLC2A3 depletion significantly decreased p53 lactylation in L6 p53 (WT) cells but not in L6 p53 (K120R) cells (Figure 3K). In contrast, disruption of SLC2A3 in L6 p53(WT) cells significantly increased p53 acetylation but had no effect on the catalysis of p53 acetylation in L6 p53 (K120R) cells (Figure 3L). Direct inhibition of LDHA (with FX‐11) confirmed that lactate production is the upstream driver of p53 K120 lactylation, as it significantly reduced both intracellular lactate levels and this specific modification (Figure 3M,N). Chromatin immunoprecipitation (ChIP) assays revealed that exogenous lactate treatment significantly diminished p53 enrichment at the promoters of its target genes, *PUMA* and *BAX* (Figure 3O,P). Critically, the inhibitory effect of SLC2A3 knockdown on cell migration—observed in control cells—was substantially weakened in p53‐K120R mutant cells (Figure S3M,N). These results indicate the functional importance of this lactylation site. We established p53‐K117R knock‐in cell lines in which K117 (K120 in humans) is replaced by arginine as rescue models, which specifically prevent lactylation modification at the K117 site of the p53 protein [48]. In an in vivo bone metastasis model established by intracardiac injection, compared to wild‐type controls, the anti‐metastatic effect of SLC2A3 knockdown was significantly attenuated in p53‐K117R knock‐in cells (Figure 3Q,R). Taken together, our results suggest that K120 is the main site of SLC2A3‐LA‐mediated p53 lactylation (Figure 3S).

### 2.4 SLC2A3 regulates LA Derived from Lung Cancer Bone Metastasis Cells to Promote Osteoclast Differentiation

**FIGURE 4:**
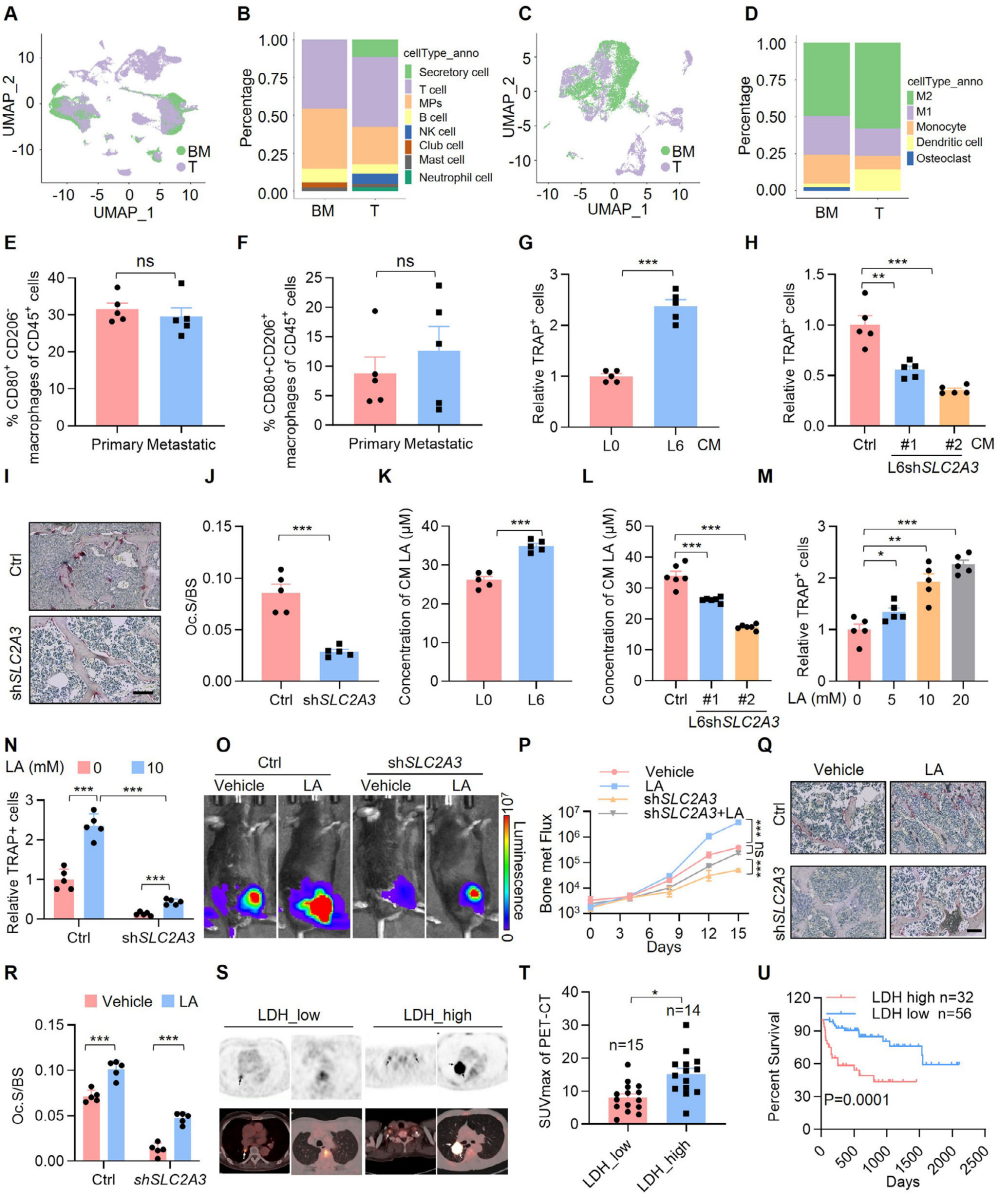
SLC2A3 regulates LA accumulation to promote osteoclast differentiation. (A) UMAP of the distribution and proportion of primary lung cancer cells (T) and bone metastatic lung cancer cells (BM) within immune cell clusters. The samples were derived from patient tissues. (B) Stacked bar graph shows the proportions of different type of immune cells in primary lung cancer tissues (T) and bone metastatic lung cancer tissues (BM). (C) UMAP of the distribution and proportion of primary lung cancer cells (T) and bone metastatic lung cancer cells (BM) within myeloid immune cell clusters. The samples were derived from patient tissues. (D) Stacked bar graph shows the proportions of different type of myeloid immune cells in primary lung cancer tissues (T) and bone metastatic lung cancer tissues (BM). (E, F) FACS quantitative analysis shows the effects on immune cell infiltration within the tumor from the mice in situ injection (*n* = 5) and via intratibial injection (*n* = 5) in mice, including M1‐like macrophages and M2‐like macrophages. *P* values were measured by unpaired two‐tailed Student's *t* test. (G) Statistical analysis of the number of Trap^+^ cells of osteoclast differentiation from bone marrow macrophages (BMMs) treated with L0 or L6 CM. Each value represents mean ± SEM (*n* = 5). ^***^ *p* < 0.001. *p* value was measured by unpaired two‐tailed Student's *t* test. (H) Statistical analysis of the number of Trap^+^ cells of osteoclast differentiation from BMMs treated with L6‐Ctrl or L6‐shSLC2A3 CM. The relative osteoclast differentiation ability was normalized to vehicle‐treated control. Each value represents mean ± SEM (*n* = 5). ^**^ *p* < 0.01; ^***^ *p* < 0.001; *p* values were measured by one‐way ANOVA with Tukey's multiple comparison test. (I, J) Representative TRAP staining images from (Figure 1R) mice with intratibial injection model. Scale bars: 100 µm. Osteoclast surface area / bone surface area (Oc.S/BS) was quantified. Each value represents mean ± SEM (*n* = 5), ^***^ *p* < 0.001; *p* value was measured by unpaired two‐tailed Student's *t* test. (K) Bar graph shows LA levels in CM of primary and metastatic lung cancer cells. Each value represents mean ± SEM (*n* = 5). ^***^ *p* < 0.001; *p* value was measured by unpaired two‐tailed Student's *t* test. (L) Bar graph shows LA levels in CM of L6 cells with or without SLC2A3 knockdown. Each value represents mean ± SEM (*n* = 5). ^***^ *p* < 0.001; *p* values were measured by one‐way ANOVA with Tukey's multiple comparison test. (M) Statistical analysis of the number of Trap^+^ cells of osteoclast differentiation from BMMs treated with LA at the concentration of 5 mM, 10 mM and 20 mM. The relative osteoclast differentiation ability was normalized to vehicle‐treated control. Each value represents mean ± SEM (*n* = 5). ^*^ *p* < 0.05; ^**^ *p* < 0.01; ^***^ *p* < 0.001; *p* values were measured by one‐way ANOVA with Tukey's multiple comparison test. (N) Statistical analysis of the number of Trap^+^ cells osteoclast differentiation from BMMs treated with L6‐Ctrl or L6‐shSLC2A3 CM and supplemented with or without LA (10 mM). The relative osteoclast differentiation ability was normalized to vehicle‐treated control. Each value represents mean ± SEM (*n* = 5). ^*^ *p* < 0.05; ^**^ *p* < 0.01; ^***^ *p* < 0.001; *p* values were measured by one‐way ANOVA with Tukey's multiple comparison test. (O) Representative IVIS images illustrate tumor growth. C57BL/6 mice were injected via intratibial injection with CMT167‐Ctrl or CMT167‐shSLC2A3 cells and treated with either vehicle or LA (1 mM of 100 µL). IVIS images were captured once every three days since treatment with LA via intraperitoneal (i.p.) injection, the intratibial injection model was lasted for 15 days. (P) BLI of the four groups in (O). BLI represents the tumor burden in the tibia of the mice. Two‐way ANOVA was used to compare groups, ^***^ *p* < 0.001. (Q, R) Representative TRAP staining images from mice with intratibial injection model in (O). Scale bars: 100 µm. Osteoclast surface area / bone surface area (Oc.S/BS) was quantified. Each value represents mean ± SEM (*n* = 5), ^***^ *p* < 0.001; *p* values were measured by one‐way ANOVA with Tukey's multiple comparison test. (S) Representative PET‐CT images show pulmonary tumors and spinal metastatic lesions in lung cancer patients with high (> 250 U/L) or low (≤ 250 U/L) LDH levels. (T) Correlation analysis between the maximum ^18^F‐FDG uptake value (SUV_max_) and the level of LDH in fasting blood in patients. Each value represents mean ± SEM (LDH_low: *n* = 15, LDH‐high: *n* = 14), ^*^ *p* < 0.05, *p* value was measured by unpaired two‐tailed Student's *t* test. (U) Kaplan‐Meier survival analysis was used to compare patient survival, with patients classified into a low‐group (≤ 250 U/L) (*n* = 56) and a high‐LDH group (> 250 U/L) (*n* = 32) based on normal fasting LDH levels as the criterion. ^***^ *p* = 0.0001 by log‐rank test.

The TME represents a complex ecosystem harboring cancer cells, stromal cells, and immune cells. This complicated network orchestrates crucial aspects of tumor bone metastasis [50, 51]. To identify the potential role of TME alterations in SLC2A3‐mediated lung cancer bone metastasis, we analyzed a single‐cell atlas of human lung cancer bone metastasis, with a focus on immune cells. Uniform manifold approximation and projection (UMAP) classified a total of 64, 156 cells into eight distinct clusters: secretory cells, T cells, mononuclear cells (MPSs), B cells, natural killer (NK) cells, club cells, mast cells and neutrophils (Figure 4A,B). The proportions of the T‐cell and mononuclear cell populations were significantly lower in the lung cancer bone metastatic fraction than in the primary lung cancer fractions (Figure 4B). Subclustering of mononuclear cells indicated a marked increase in the number of osteoclasts and M1‐like macrophages and a decrease in the number of M2‐like macrophages and dendritic cells in lung cancer bone metastasis tumors (Figure 4C,D). Next, we developed an orthotopic lung cancer model by direct injection of CMT167 cells into the left lung parenchyma of immunocompetent C57BL/6 mice, followed by flow cytometric profiling of TME alterations. M1‐like macrophages and M2‐like macrophages were unchanged in lung cancer bone metastatic tumors compared with primary lung cancer tumors (Figure 4E,F; Figure S4A). Preinduced primary preosteoclasts were exposed to conditioned medium (CM) from lung cancer bone metastatic cells (L6) and primary lung cancer cells (L0). Tartrate‐resistant acid phosphatase (TRAP) staining revealed that the number of TRAP^+^ osteoclasts induced by L6 CM was significantly greater than that induced by L0 CM (Figure 4G; Figure S4B). Osteoclasts produce significant amounts of acid and proteases to help degrade bone, which contributes to lung cancer bone metastasis [39, 51].

Next, we examined whether SLC2A3 regulates osteoclast differentiation to mediate the bone metastasis of lung cancer. The TRAP staining results revealed that CM from SLC2A3‐KD cells markedly inhibited osteoclasts formation compared to control CM. (Figure 4H; Figure S4C). We further investigated osteoclast differentiation in intracardially injected mice (Figure 4I). The number of osteoclasts in the bones of shSLC2A3 mice appeared to be significantly lower than that in the bones of control mice (Figure 4J). Increased aerobic glycolysis and glycolysis‐derived LA production have been reported to be positively related to osteoclast‐mediated bone resorption [52], and we wondered whether LA production in the TME contributes to osteoclast differentiation. The results revealed that LA production (Figure 4K) was significantly greater in bone metastatic CM than in primary lung cancer CM and that SLC2A3 depletion sharply attenuated LA production in CM (Figure 4L). We also compared the osteoclast differentiation ability of LA‐treated cells with that of control cells. The TRAP staining results revealed that LA treatment increased the number of TRAP^+^ osteoclasts in a dose‐dependent manner (Figure 4M; Figure S4D) and pit formation assay demonstrated that lactate treatment significantly enhanced the area of bone matrix resorption (Figure S4E,F). Moreover, we observed that SLC2A3 inhibition attenuated osteoclast differentiation (Figure 4N; Figure S4G), which was greatly diminished by LA treatment, suggesting that the osteoclast differentiation process requires LA. Mice injected with luciferase‐labeled cells into the tibia were treated with vehicle or LA for 15 days. The results revealed that LA treatment did not promote bone metastasis (Figure 4O,P) or osteoclast differentiation (Figure 4Q,R) when SLC2A3 was depleted in tumor cells. Cancer cells activate aerobic glycolysis and convert the majority of glucose into LA. LA dehydrogenase (LDHA) is a glycolytic enzyme that catalyzes the conversion of pyruvate to LA [53]. We detected radiological features and LDH levels in a PET cohort including 29 patients with lung cancer bone metastasis who had undergo ^18^F‐FDG PET/CT scans. Our results revealed that the maximum ^18^F‐FDG uptake value (SUV_max_) of patients was positively correlated with the expression of LDH (Figure 4S,T), and a high SUV_max_ value was also correlated with poor overall survival (Figure 4U), implying a potential positive correlation between LA production and patient survival. Together, these findings suggest that SLC2A3 stimulates LA production in the TME to trigger bone resorption.

Tumor cells primarily export lactate through monocarboxylate transporters MCT1 and MCT4 [46], and we examined which transporter facilitates SLC2A3‐driven lactate secretion in lung cancer bone metastatic cells. We first knocked down MCT1 or MCT4 in the bone metastatic L6 cell line and measured lactate levels in the conditioned media. The data revealed that MCT4 knockdown effectively reduced lactate secretion, while MCT1 knockdown showed minimal effect (Figure S4H–J). Furthermore, we found that SLC2A3 overexpression significantly enhanced lactate release into the media, and this effect was substantially attenuated by concurrent MCT4 knockdown rather than MCT1 inhibition (Figure S4K). Next, we collected conditioned media (CM) from these cell groups and applied them to osteoclast differentiation assays. Consistent with the lactate measurements, conditioned medium from SLC2A3‐overexpressing cells robustly promoted osteoclast differentiation, and this effect was abolished when MCT4 was concurrently knocked down (Figure S4L,M), suggesting that MCT4 serves as the primary lactate transporter responsible for SLC2A3‐driven lactate export from tumor cells. Subsequently, we performed RNA sequencing (RNA‐seq) to identify the signaling pathways through which lactate regulates osteoclast differentiation. Pathway enrichment analysis confirmed that lactate treatment significantly altered the osteoclast differentiation signaling pathway (Figure S4N) and the details of the data are in the Table S6. Given osteoclast differentiation is primarily mediated through two key signaling pathways: NF‑κB (p65) and MAPK (including p38 and ERK) [54]. Western blot validation was subsequently performed to assess the activation of these classic pathways. The results demonstrated that, among the pathways examined, only p38 and its downstream effector MAPK13 were significantly upregulated upon lactate treatment, while the phosphorylation levels of p65 (NF‑κB) and ERK remained statistically unchanged (Figure S4O). Together, these findings identify MCT4 as the key transporter for SLC2A3‐driven lactate secretion, which in turn promotes osteoclast differentiation via activation of the p38 pathway.

### 2.5 SLC2A3 Deficiency Mediates Extrinsic LA‐Mediated Destabilization of p53 Lactylation at K120 and Activation of PD‐1 Transcription in CD8^+^ T Cells

**FIGURE 5:**
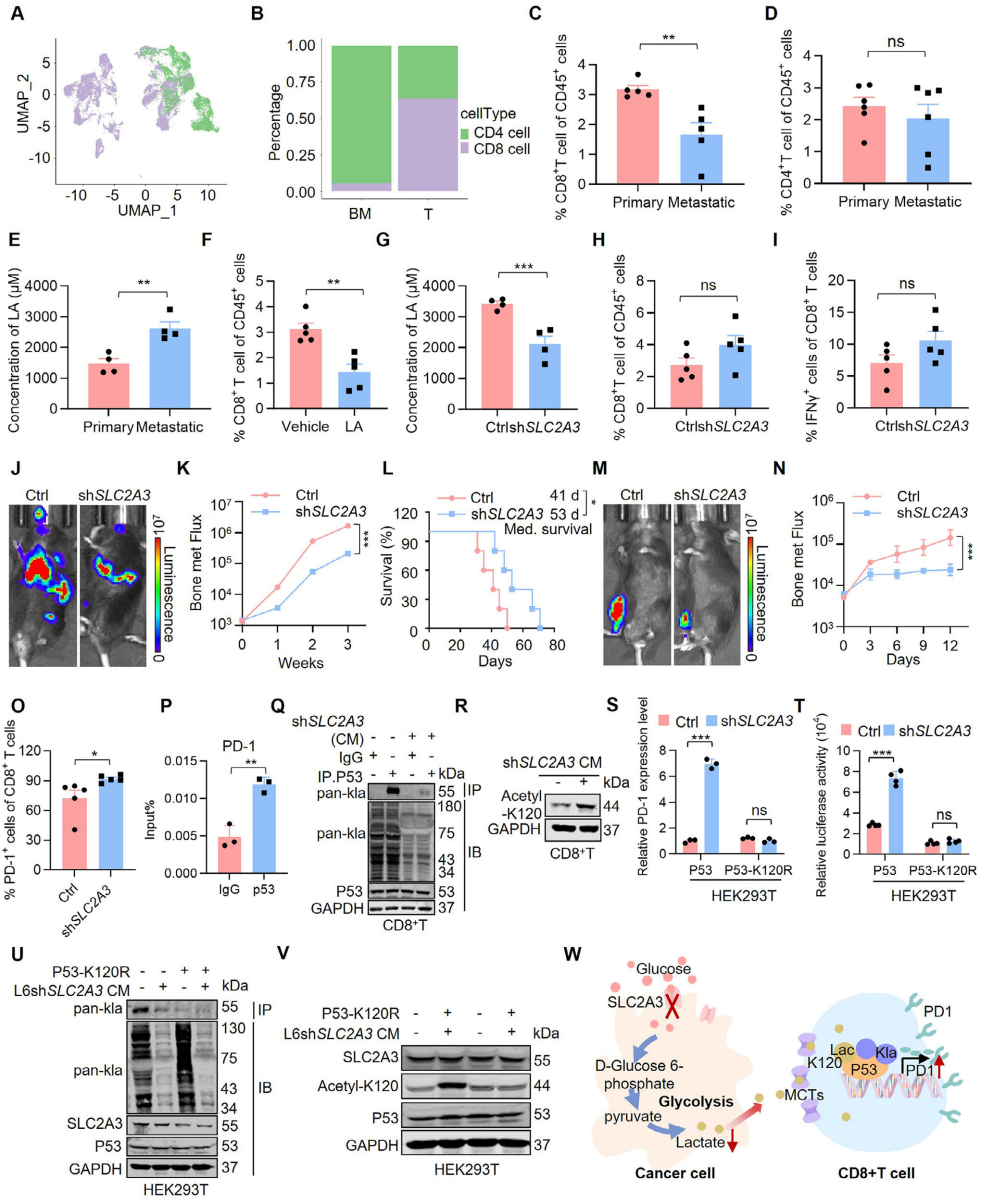
SLC2A3 deficiency‐induced LA depletion enhances PD‐1 expression in CD8^+^ T cells through reducing lactylation of p53 at K120. (A) UMAP shows the distribution of primary lung cancer cells (T) and bone metastatic lung cancer cells (BM) within T‐cell clusters. (B) Stacked bar graph shows the proportions of CD4^+^ T and CD8^+^ T cells in primary lung cancer cells (T) and bone metastatic lung cancer cells (BM). (C, D) FACS quantitative analysis shows the differences on immune cell infiltration within the tumor from the mice in situ injection and intratibial injection, including CD8^+^ T and CD4^+^ T cells. *n* = 4 mice per group. *p* value was measured by unpaired two‐tailed Student's *t* test. (E) Bar graph shows LA levels of the tumor from the mice in situ injection and intratibial injection. Each value represents mean ± SEM (*n* = 4). ^***^ *p* < 0.001. *p* value was measured by unpaired two‐tailed Student's *t* test. (F) FACS quantitative analysis shows the effect of LA treatment on CD8^+^ T cells within the tumor from the mice via intratibial injection. The group of Vehicle: *n* = 4; the group of LA treatment: *n* = 4. *p* value was measured by unpaired two‐tailed Student's *t*‐test. (G) Bar graph shows LA levels of the tumor from the mice via intratibial injection. Each value represents mean ± SEM (*n* = 4). ^***^ *p* < 0.001. *p* value was measured by unpaired two‐tailed Student's *t* test. (H, I) FACS quantitative analysis shows the effect of SLC2A3 knockdown on CD8^+^ T cells and IFNγ^+^ CD8^+^ T cells within the tumor from the mice via intratibial injection. *n* = 5 mice per group. *p* value was measured by unpaired two‐tailed Student's *t*‐test. (J) Representative IVIS images illustrate tumor growth and metastasis in C57BL/6 mice injected with CMT167‐Ctrl or CMT167‐shSLC2A3 (*n* = 5) via i.c. injection. IVIS images were captured once every three days post injection, the i.c. injection model was lasted for 21 days. (K) BLI of the two groups in (J). Two‐way ANOVA was used to compare groups, ^***^ *p* < 0.001. (L) Kaplan‐Meier survival analysis of the mice after the indicated treatments (CMT167Ctrl: *n* = 5; CMT167shSLC2A3: *n* = 5). Med., median. ^*^ *p* < 0.05 by log‐rank test. (M) Representative IVIS images illustrate tumor growth and metastasis in C57BL/6 mice injected with CMT167‐Ctrl or CMT167‐shSLC2A3 (*n* = 5) via intratibial injection. IVIS images were captured once every three days post injection, the intratibial injection model was lasted for 12 days. (N) BLI of the two groups in (M). Two‐way ANOVA was used to compare groups, ^***^ *p* < 0.001. (O) FACS quantitative analysis shows the effect of SLC2A3 knockdown on PD‐1^+^ CD8^+^ T within the tumor from the mice via intratibial injection. *n* = 5 mice per group. *p* value was measured by unpaired two‐tailed Student's *t*‐test. (P) ChIP‑qPCR analysis of p53 enrichment at the R2 region (∼−1.7 kb upstream of the PD‑1 TSS). The relative enrichment was normalized to the input control. Each value represents mean ± SEM (n = 3). ^**^ *p* < 0.01; *p* value was measured by unpaired two‑tailed Student's *t* test. (Q) CD8^+^ T cells were isolated from C57BL/6 mice spleens and stimulated with L6‐Ctrl or L6‐shSLC2A3 CM for 24 h. CD8^+^ T cells were then lysed with CHAPS lysis buffer. Immunoprecipitations of cell lysates were performed using anti‐p53 or anti‐IgG, followed by immunoblotting with antibodies against the indicated proteins. (R) Western blot analysis of p53 (acetyl‐K120) expression in CD8^+^ T cells after stimulating with L6‐Ctrl or L6‐shSLC2A3 CM. (S) Relative mRNA expression of PD‐1 in HEK293T cells. SLC2A3 knockdown increased the PD‐1 expression. Mutation at p53‐K120 abrogated this effect. Each value represents mean ± SEM (*n* = 4). ^***^ *p* < 0.001; *p* values were measured by one‐way ANOVA with Tukey's multiple comparison test. (T) Luciferase reporter assay shows that SLC2A3 knockdown increased the reporter gene expression in HEK293T cells. Mutation at p53‐K120 abrogated this effect. Luciferase reporter vectors with promoters contain the indicated PD‐1 promoter regions. ^***^ *p* < 0.001; *p* values were measured by one‐way ANOVA with Tukey's multiple comparison test. (U) HEK293T‐p53‐WT and HEK293T‐p53‐K120R cells after stimulating with L6‐Ctrl or L6‐shSLC2A3 CM were lysed with CHAPS lysis buffer. Immunoprecipitations of cell lysates with anti‐HA agarose, followed by immunoblotting with antibodies against the indicated proteins. (V) Western blot analysis of p53 (acetyl‐K120) expression in HEK293T‐p53‐WT and HEK293T‐p53‐K120R cells after stimulating with L6Ctrl or L6shSLC2A3 CM. (W) Schematic diagram illustrates p53 lactylation‐mediated regulation of PD‐1 transcription in CD8^+^ T cells.

The proportions of the T‐cell populations were also significantly lower in the lung cancer bone metastatic fraction than in the primary lung cancer fractions (Figure 4A,B). Subclustering of T cells indicated a marked decrease in CD8^+^ T cells and an increase in CD4^+^ T cells in lung cancer bone metastasis tumors (Figure 5A,B). Next, we characterized the flow cytometric profile of T‐cell alterations in an orthotopic lung cancer model. Flow cytometry analysis revealed reduced frequencies of CD8^+^ T cells (Figure 5C; Figure S5A) and unchanged frequencies of CD4^+^ T cells (Figure 5D) in lung cancer bone metastatic tumors compared with those in primary lung cancer tumors. Furthermore, high concentrations of tumor‐derived LA in the TME reportedly impede LA efflux in CD8^+^ T cells, thereby affecting their metabolism and effector functions [55, 56].

Next, we examined whether LA regulates CD8^+^ T cells function in the TME. The results revealed that LA production in the TME was augmented in lung cancer bone metastatic tumors but not in primary lung tumors (Figure 5E), and LA treatment markedly inhibited CD8^+^ T cells (Figure 5F; Figure S5E). Notably, SLC2A3 depletion sharply decreased LA production in the TME (Figure 5G). However, SLC2A3‐KD induction in activated CD8^+^ T cells was minimal (Figure 5H,I; Figure S5F,G). In addition, we performed an i.c. injection of luciferase‐labeled CMT167 cells into immunocompetent C57BL/6 mice. Compared with immunodeficient hosts (Figure 1L,M), immunocompetent hosts did not potentiate the inhibitory effect of SLC2A3 knockdown on lung cancer metastatic capacity (Figure 5J,K) or prolong mouse survival (Figure 5L). Consistent results were observed in the intratibial injection models (Figure 5M,N).

Previous reports have suggested that PD‐1 is a key negative regulator of CD8^+^ T‐cell activation [57] and that CD8^+^ T cells exhibit lower PD‐1 expression in highly glycolytic tumors [58]. We hypothesized that PD‐1 expression by CD8^+^ T cells was upregulated in SLC2A3 depletion‐induced low‐glycolytic tumors. Notably, PD‐1 expression by CD8^+^ T cells was significantly elevated with SLC2A3 inhibition (Figure 5O; Figure S5H). Given direct binding of p53 to PD‐1, approximately −1.7 kb upstream of the Transcription Start Site (TSS) [59]. ChIP‐qPCR demonstrated significant enrichment of p53 specifically at the R2 region (∼−1.7 kb upstream of the TSS) (Figure 5P). We next investigated factors impacting PD‐1 expression by CD8^+^ T cells in the TME. As p53 is a master regulator that transcriptionally activates PD‐1 by acetylation at K120 in cancer cells [59], we asked whether p53 regulates PD‐1 expression by lactylation at K120 in CD8^+^ T cells. The results revealed that SLC2A3 depletion CM sharply decreased p53 lactylation (Figure 5Q; Figure S5I) and increased p53 acetylation in CD8^+^ T cells (Figure 5R).

Furthermore, we determined whether SLC2A3‐KD CM regulated p53 lactylation at K120, which inhibits PD‐1 transcriptional activation, by generating a pair of HEK293T‐inducible cell lines that expressed wild‐type p53 and an acetylation‐deficient p53 mutant. As shown in Figure 5S and Figure 5T, SLC2A3‐KD CM promoted PD‐1 expression in wild‐type p53‐overexpressing cells, whereas the p53 (K120R) mutant almost completely lost the ability to induce PD‐1 transcription. In addition, SLC2A3‐KD CM significantly decreased p53 lactylation and increased p53 acetylation in p53 (WT) cells but had no effect on the catalysis of p53 lactylation and acetylation in p53 (K120R) cells (Figure 5U,V; Figure S5J). Together, our data suggest that the lactylation of K120 upon p53 activation is critically involved in SLC2A3‐mediated PD‐1 transcription (Figure 5W).

### 2.6 Paris Saponin VII, a Potent SLC2A3 Inhibitor, Suppresses Lung Cancer Bone Metastasis

**FIGURE 6:**
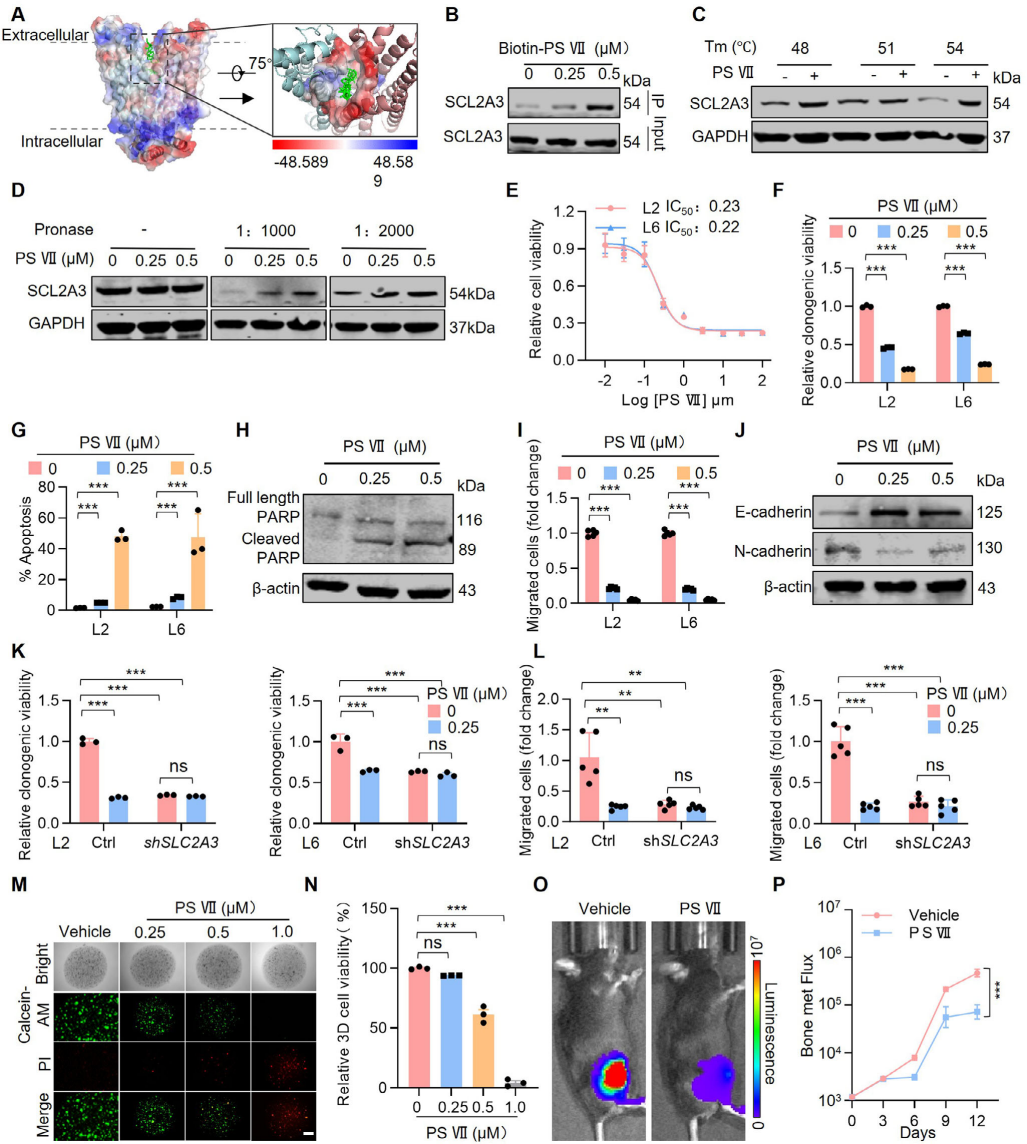
Paris saponin VII as a potent SLC2A3 inhibitor suppresses lung cancer bone metastasis. (A) Crystal structure of Paris saponin VII‐bound SLC2A3 (SLC2A3‐Paris saponin VII) in an outward‐facing state. SLC2A3exo is domain colored with cyan, rose and purple for the N‐terminal, C‐terminal, and ICH domains, respectively. (B) L6 protein lysates were treated with biotin or biotin‐Paris saponin VII (biotin‐PS VII) for 3 h, performed pull‐down using Streptavidin Agarose Resin, followed by western blot. (C) The DARTS method was used for drug target identification. Immunoblotting shows that Paris saponin VII protects the SLC2A3 subunit from pronase proteolysis. (D) The CETSA method was used for drug‐binding thermal stability. Immunoblotting shows that Paris saponin VII protects the SLC2A3 from thermal degradation. (E) Inhibitory effects of Paris saponin VII on L2 and L6 isogenic cell lines. (F) The colony‐forming ability of metastatic cell lines after treatment with Paris saponin VII at the concentration of 0.25 µM and 0.5 µM. The relative clonogenic viability was normalized to vehicle‐treated control. Each value represents mean ± SEM (*n* = 5). ^***^ *p* < 0.001; *p* values were measured by one‐way ANOVA with Tukey's multiple comparison test. (G) Statistical analysis of the flow cytometry apoptosis in L2 and L6 cells after Paris saponin VII treatment. Each value represents mean ± SEM (*n* = 3). ^***^ *p* < 0.001; *p* values were measured by one‐way ANOVA with Tukey's multiple comparison test. (H) Western blot analysis of PARP1 expression in L6 cells after treatment with Paris saponin VII. (I) The migration ability of metastatic cell lines after treatment with Paris saponin VII at the concentration of 0.25 µM and 0.5 µM. The relative migration ability was normalized to vehicle‐treated control. Each value represents mean ± SEM (*n* = 3). ^***^ *p* < 0.001; *p* values were measured by one‐way ANOVA with Tukey's multiple comparison test. (J) Western blot analysis of EMT markers in L6 cells after treatment with Paris saponin VII. (K) The colony‐forming ability of metastatic cells after SLC2A3 knockdown and treatment with Paris saponin VII at the concentration of 0.25 µM. The relative clonogenic viability was normalized to vehicle‐treated control. Each value represents mean ± SEM (n = 3). ^***^ *p* < 0.001; *p* values were measured by one‐way ANOVA with Tukey's multiple comparison test. (L) The migration ability of metastatic cells after SLC2A3 knockdown and treatment with Paris saponin VII at the concentration of 0.25 µM. The relative migration viability was normalized to vehicle‐treated control. Each value represents mean ± SEM (*n* = 5). ^***^ *p* < 0.001; *p* values were measured by one‐way ANOVA with Tukey's multiple comparison test. (M) Immunofluorescent images of the organoids after treatment with Paris saponin VII for four days, then stained by the Calcein/PI kit. Scale bar: 400 µm. (N) Relative quantification of the viability of the 3D organoids. Error bars are mean ± SEM. *P* values were calculated using one‐way ANOVA with Tukey's multiple comparison test, ^***^ *p* < 0.001. (O) Representative IVIS images illustrate tumor growth in C57BL/6 mice injected with CMT167. After 3 days of intratibial injection, mice were treated with Paris saponin VII (2 mg/kg, *n* = 5) via i.p. injection for an additional 12 days (*n*  =  5 per group). (P) BLI of the two groups in (O). Two‐way ANOVA was used to compare groups, ^***^ *p* < 0.001.

To investigate the therapeutic potential of blocking the activity of SLC2A3 as a targeting strategy in lung cancer bone metastasis, we sought to develop an SLC2A3 inhibitor by using the cryo‐EM structure of the glycosylation site‐eliminated variant SLC2A3 (N43T). The transmembrane region of GLUT3 (N43T) contains a canonical major facilitator superfamily (MFS) fold with 12 transmembrane segments (TMs) folded into the N‐terminal and C‐terminal domains, each comprising “3+3” inverted repeats [28, 60]. The binding models confirmed that Paris saponin VII is unambiguously resolved in the central cavity, which opens to the extracellular side (Figure 6A). Next, we applied biotin‐tagged Paris saponin VII (hereafter Bio‐PS VII) to reveal the potential direct target of Paris saponin VII. Upon incubation with senescent cell lysates, Bio‐PS VII bound to proteins before streptavidin‐agarose beads were applied to pull down the Bio‐PS VII‐protein complex for western blotting. We observed that the PS VII protein interacts with the SLC2A3 protein, but not other glucose transporters, SLC2A1 or SLC2A4 (Figure 6B; Figure S6A). The Glucose Uptake results showed that PS VII treatment still significantly inhibited glucose uptake in SLC2A1 or SLC2A4 knockdown cells, to an extent similar to its effect in control cells (Figure S6B), indicating a selective interaction between PS VII and SLC2A3. To further discover bona fide target proteins, we followed another approach to analyze protein thermal stabilization after a ligand binding cellular thermal shift assay (CETSA) in the temperature range from 48 to 57°C. We observed an increase in the stability of SLC2A3 upon incubation with PS VII compared with the vehicle (Figure 6C). To validate target identification, we employed drug affinity responsive target stability (DARTS) assays, which confirmed binding between SLC2A3 and PS VII through increased proteolytic stability of SLC2A3 (Figure 6D). These data suggest that PS VII binds to and occupies the SLC2A3‐bound pocket.

We next examined the cytotoxicity of PS VII in lung cancer bone metastatic cells. Our results revealed that treatment with PS VII led to a concentration‐dependent decrease in the viability of L2 and L6 cells, with IC_50_ values of 0.23 and 0.22 µM, respectively (Figure 6E). We also analyzed the clonogenic growth of L2 and L6 cells after exposure to different concentrations of PS VII. Our findings demonstrated that PS VII effectively suppressed the clonogenic growth of lung cancer bone metastatic cells (Figure 6F; Figure S6C). Furthermore, we measured the apoptotic capacity by Annexin V staining and observed that PS VII increased the level of apoptosis in lung cancer bone metastasis cells (Figure 6G; Figure S6D) and the levels of the apoptosis‐related proteins cleaved PARP (Cl‐PARP) and cleaved caspase‐3 (Cl‐caspase‐3) (Figure 6H; Figure S6E). In addition, transwell assays revealed that PS VII inhibited the migration of lung cancer bone metastatic cells in a dose‐dependent manner (Figure 6I; Figure S6F), and western blot analysis revealed that PS VII markedly increased E‐cadherin levels and decreased N‐cadherin levels (Figure 6G). We further validated the on‐target specificity of PS VII, and colony formation and transwell assays were conducted to characterize the cytotoxicity alterations induced by PS VII treatment or SLC2A3 knockdown in L2 and L6 cells. The results showed that PS VII did not reduce the growth (Figure 6K; Figure S6G) or migration (Figure 6L; Figure S6H) ability of SLC2A3‐KD cells. These data suggest that the inhibitory effect of PS VII on lung cancer bone metastasis occurs mainly through the targeting of SLC2A3.

To gain a better understanding of PS VII and develop a treatment strategy based on its use, we generated patient‐derived organoids (PDOs) as a disease model and conducted drug treatment assays to assess the therapeutic potential of PS VII. PS VII strikingly suppressed organoids growth at the tested concentrations (Figure 6M,N). In parallel, we generated intratibial injection mouse models to evaluate the therapeutic efficacy of PS VII against lung cancer bone metastasis in vivo. As shown in Figure 6O,P, tumor growth was significantly inhibited in the PS VII‐treated groups than in the vehicle control group. These findings demonstrate that the SLC2A3 inhibitor PS VII has a potent inhibitory effect on lung cancer bone metastasis in vivo. We further investigated the pharmacokinetics and toxicity of PS VII in ICR mice. Toxicity assessment at various doses (2, 5, 10, and 20 mg/kg) revealed that PS VII was well tolerated at doses 2 mg/kg, with no significant body weight changes, signs of toxicity, and blood biochemistry parameters (Figure S6I–K). In contrast, higher doses (10 and 20 mg/kg) induced significant body weight loss and mortality. Following a single intraperitoneal (i.p.) injection and an intravenous (i.v.) injection of Paris saponin VII (at 10 mg/kg and 1 mg/kg, respectively), its pharmacokinetics were analyzed. Following intravenous injection, PS VII exhibited a half‑life of 2.40 ± 0.33 h, a maximum concentration (Cmax) of 868.0 ± 72.6 ng/mL at 0.0833 h, and an area under the curve of 1796 ± 250 h·ng/mL. In contrast, intraperitoneal injection resulted in a half‐life of 33.80 ± 3.72 h, with Cmax reaching 2310 ± 322 ng/mL at 8 h and an AUC of 7632 ± 284 h·ng/mL (Figure S6L), supporting its biological activity in our in vivo dosing regimen.

### 2.7 Paris Saponin VII Attenuates Lung Cancer Bone Metastasis by Reducing LA

**FIGURE 7:**
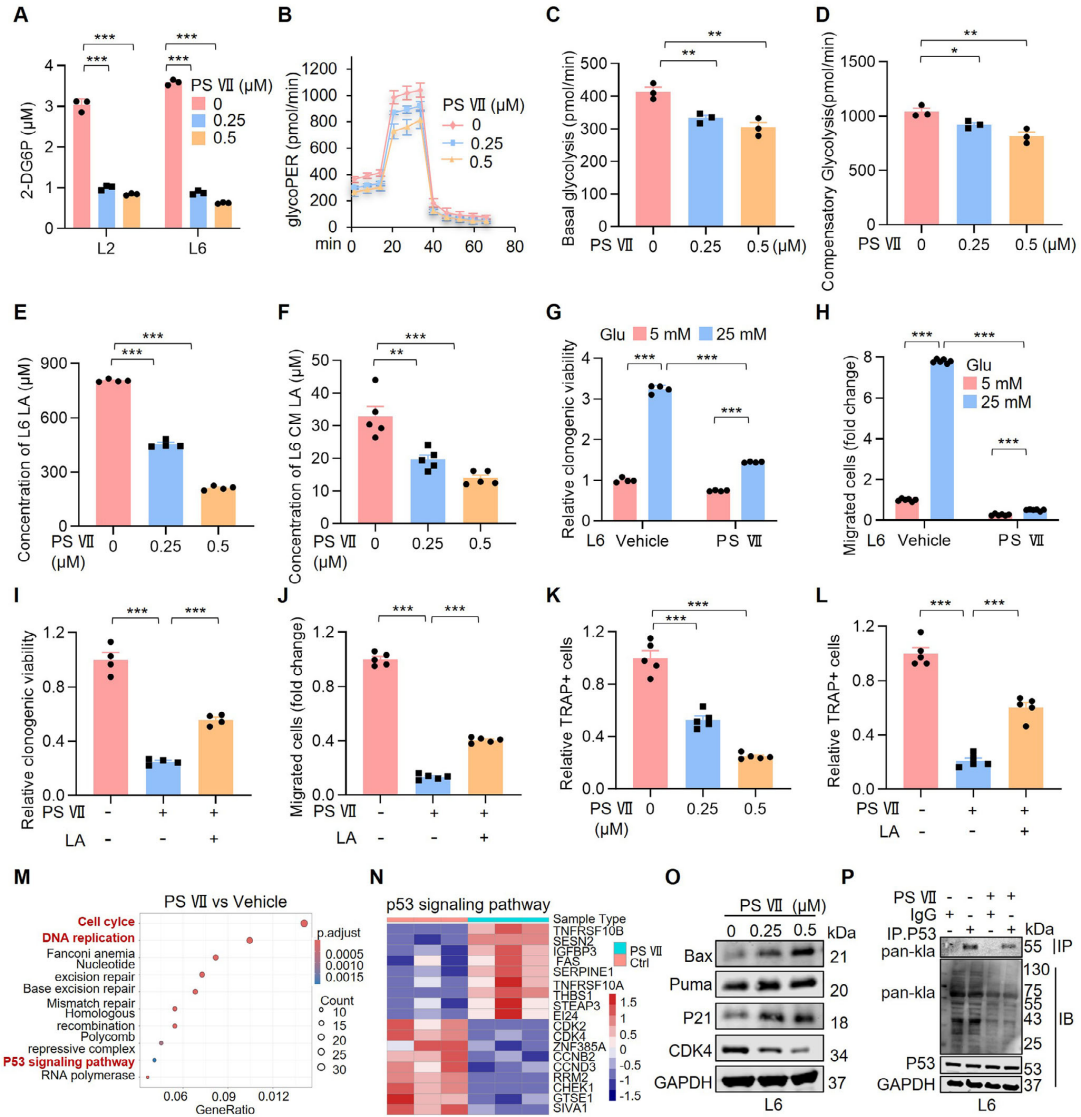
Paris saponin VII inhibits lung cancer bone metastasis by reducing LA secretion. (A) Glucose uptake capacity assay in metastatic lung cancer cells after treatment with Paris saponin VII. Each value represents mean ± SEM (*n* = 3). ^***^ *p* < 0.001; *p* values were measured by one‐way ANOVA with Tukey's multiple comparison test. (B–D) Glycolysis rate assay (*n* = 3) was conducted on metastatic lung cancer cells after treatment with Paris saponin VII. Each value represents mean ± StDev (*n* = 3) ^*^ *p* <0.05; ^**^ *p* <0.01. (E) Bar graph shows the LA levels in L6 cells after treatment with Paris saponin VII. Each value represents mean ± SEM (*n* = 4). ^***^ *p* < 0.001; *p* values were measured by one‐way ANOVA with Tukey's multiple comparison test. (F) Bar graph shows LA levels in CM of L6 cells after treatment with Paris saponin VII. Each value represents mean ± SEM (*n* = 5). ^**^ *p* < 0.01, ^***^ *p* < 0.001; *p* values were measured by one‐way ANOVA with Tukey's multiple comparison test. (G) L6 cells were treated with glucose (5 mM or 25 mM) and Paris saponin VII (0.25 µM). The relative clonogenic viability was normalized to vehicle‐treated control. Each value represents mean ± SEM (*n* = 4). ^***^ *p* < 0.001; *p* values were measured by one‐way ANOVA with Tukey's multiple comparison test. (H) L6 cells were treated with glucose (5 mM or 25 mM) and Paris saponin VII (0.25 µM). The relative migration ability was normalized to vehicle‐treated control. Each value represents mean ± SEM (*n* = 6). ^***^ *p* < 0.001; *p* values were measured by one‐way ANOVA with Tukey's multiple comparison test. (I) Statistical analysis of the colony formation results of L6 cells after LA (20 mM) and Paris saponin VII (0.25 µM) treatment. The relative clonogenic viability was normalized to vehicle‐treated control. Each value represents mean ± SEM (*n* = 4). ^***^ *p* < 0.001; *p* values were measured by one‐way ANOVA with Tukey's multiple comparison test. (J) Statistical analysis of the number of migrated cells in L6 cells after LA (20 mM) and Paris saponin VII (0.25 µM) treatment. The relative migration ability was normalized to vehicle‐treated control. Each value represents mean ± SEM (*n* = 5). ^***^ *p* < 0.001; *p* values were measured by one‐way ANOVA with Tukey's multiple comparison test. (K) Statistical analysis of the number of Trap^+^ cells of osteoclast differentiation from BMMs. The relative osteoclast differentiation ability was normalized to vehicle‐treated control. The osteoclast differentiation of BMMs was induced by conditioned media collected from L6 cells pretreated with varying concentrations (0, 0.25, 0.5 µM) of Paris saponin VII. Each value represents mean ± SEM (*n* = 5). ^***^ *p* < 0.001; *p* values were measured by one‐way ANOVA with Tukey's multiple comparison test. (L) Effect of conditioned medium from Paris saponin VII‐treated cells versus direct lactate stimulation on osteoclast differentiation. Cells were first treated with 0.25 mM Paris saponin VII, and the conditioned medium was collected to stimulate osteoclast differentiation. This was compared with direct stimulation using 20 mM LA. The relative osteoclast differentiation ability was normalized to vehicle‐treated control. Each value represents mean ± SEM (*n* = 5). ^***^ *p* < 0.001; *p* values were measured by one‐way ANOVA with Tukey's multiple comparison test. (M) Pathway enrichment analysis reveals the signaling pathways downregulated in L6 cells after treatment with Paris saponin VII (0.25 µM) (*n* = 3 per group). (N) Heatmap of significantly differentially expressed proteins involved in p53 signaling pathway in L6 cells after Paris saponin VII treatment (0.25 µM) (*n* = 3 per group). (O) Western blot analysis of p53 signaling pathway markers after treatment with Paris saponin VII (0.25 µM). (P) L6 cells were treated with Paris saponin VII (0.25 µM) for 12 h, and whole cell extracts (WCEs) were collected for IP with anti‐p53 antibody, followed by immunoblotting.

To elucidate the downstream molecular mechanism underlying the anti‐bone‐metastasis effect of PS VII targeting SLC2A3, alterations in glucose metabolism processes were assessed. The results revealed that PS VII significantly impaired glucose uptake (Figure 7A), glycolytic capacity (Figure 7B–D), the cellular LA level (Figure 7E), and the CM LA level (Figure 7F). We also observed that PS VII treatment reduced cancer cell growth (Figure 7G; Figure S7A) and migration (Figure 7H; Figure S7B), and this effect was greatly diminished by high‐glucose conditions and PS VII treatment, suggesting that PS VII attenuates SLC2A3‐mediated glucose metabolism. Moreover, we observed that LA treatment greatly attenuated the inhibition of cell growth (Figure 7I; Figure S7C,D) and migration (Figure 7J; Figure S7E) caused by PS VII treatment. The TRAP staining results revealed that PS VII treatment decreased the number of TRAP^+^ osteoclasts in a dose‐dependent manner (Figure 7K; Figure S7F), which was greatly diminished by LA treatment (Figure 7L; Figure S7G). These findings demonstrate that LA is required for the attenuation of metastasis by PS VII.

To further investigate whether PS VII protects against lung cancer bone metastasis by regulating the p53 lactylation signaling pathway, proteomic analysis was performed to analyze the activation of p53 signaling. After PS VII treatment, KEGG analysis revealed that these differentially regulated proteins were enriched in pathways related to the p53 signaling pathway and its downstream signaling pathways, including the cell cycle and DNA replication pathways (Figure 7M). We also noted that the expression of apoptosis‐related proteins in the p53 signaling pathway (Puma and BAX) and the expression of a cell cycle‐related protein (p21) were significantly increased by PS VII treatment (Figure 7N,O). PS VII treatment sharply decreased p53 lactylation (Figure 7P; Figure S7H). These data suggest that PS VII suppresses lung cancer bone metastasis by SLC2A3‐mediated p53 lactylation.

### 2.8 Targeting SLC2A3‐Mediated LA Metabolism Increases the Sensitivity of Lung Cancer Bone Metastasis to anti‐PD‐1 Treatment

**FIGURE 8:**
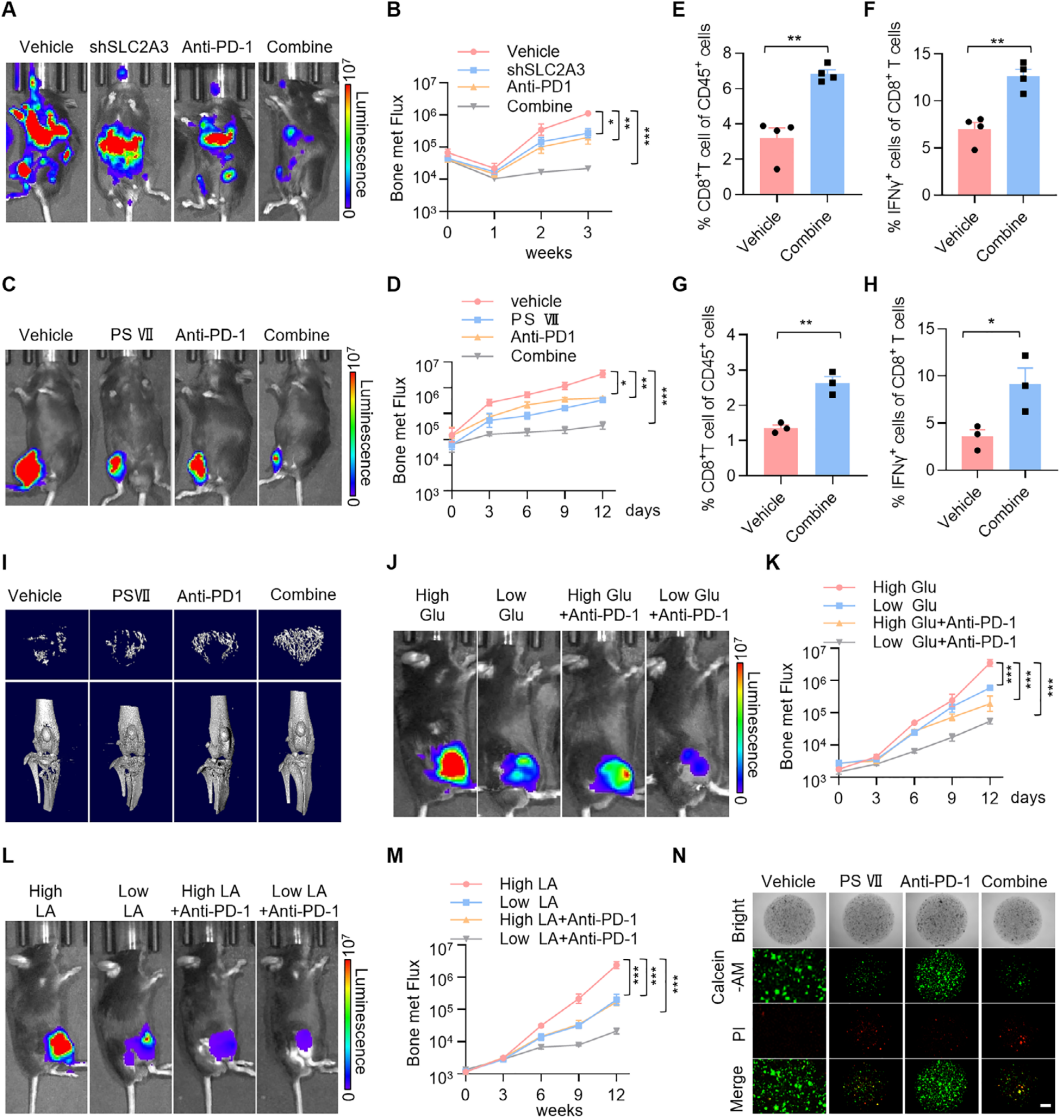
Paris saponin VII enhances the sensitivity of lung cancer bone metastasis to anti‐PD‐1 treatment. (A) Representative IVIS images of indicated mice. After 7 days of i.c. injection with CMT167‐Ctrl or CMT167‐shSLC2A3 cells, mice were treated with PD‐1 antibody (50 µg) via i.p. injection for an additional 21 days (*n*  =  5 per group). (B) BLI of the four groups in (A). Two‐way ANOVA was used to compare groups, ^*^ *p* < 0.05, ^***^ *p* < 0.01, ^***^ *p* < 0.001. (C) Representative IVIS images of indicated mice. After 3 days of intratibial injection with CMT167 cells, mice were treated with Paris saponin VII (2 mg/kg) and PD‐1 antibody (50 µg) via i.p. injection for an additional 12 days (*n*  =  5 per group). (D) BLI of the four groups in (C). Two‐way ANOVA was used to compare groups, ^*^ *p* < 0.05, ^**^ *p* < 0.01, ^***^ *p* < 0.001. (E, F) FACS quantitative analysis shows the effects of Paris saponin VII and anti‐PD‐1 treatment on CD8^+^ T cells and IFNγ^+^ CD8^+^ T cells infiltration within the tumor from the mice via i.c. injection. *n* = 4 mice per group. ^**^ *p* < 0.01, *p* values were measured by unpaired two‐tailed Student's *t* test. (G, H) FACS quantitative analysis shows the effects of Paris saponin VII and anti‐PD‐1 treatment on CD8^+^ T cells and IFNγ^+^ CD8^+^ T cells infiltration within the tumor from the mice via intratibial injection. *n* = 3 mice per group. ^*^ *p* < 0.05, ^***^ *p* < 0.001 *p* values were measured by unpaired two‐tailed Student's *t* test. (I) Representative micro‐CT images of the tibia and trabecular bone from the mice in (C). (J) Representative IVIS images of indicated mice. After 3 days of intratibial injection with CMT167 cells, mice were given glucose (High glucose:10% glucose in water; Low glucose: 1% glucose in water) and treated with PD‐1 antibody (50 µg) via i.p. injection for an additional 12 days (*n*  =  5 per group). (K) BLI of the four groups in (J). Two‐way ANOVA was used to compare groups, ^***^ *p* < 0.001. (L) Representative IVIS images of indicated mice. After 3 days of intratibial injection with CMT167 cells, mice were treated with LA (Low: 0.2 mM, High: 1 mM) and PD‐1 antibody (50 µg) via i.p. injection for an additional 12 days (*n*  =  5 per group). (M) BLI of the four groups in (J). Two‐way ANOVA was used to compare groups, ^***^ *p* < 0.001. (N) Immunofluorescent images of the organoids after treatment with Paris saponin VII (1 µM) and PD‐1 antibody (40 mg/mL) for four days, then stained by the Calcein/PI kit. Scale bar: 400 µm.

The data in Figure 5R,S indicate that SLC2A3 inhibition in lung cancer bone metastatic cells elevated PD‐1 transcriptional activation in CD8^+^ T cells. We investigated whether SLC2A3 inhibition improves the therapeutic effects of anti‐PD‐1 treatment on lung cancer bone metastasis. C57BL/6J mice with palpable tumors were treated with vehicle, genetic or pharmacological (PS VII) inhibition of SLC2A3 alone, anti‐PD‐1 alone, or the combination of SLC2A3 inhibition and anti‐PD‐1. The results revealed that genetic or pharmacological (PS VII) inhibition of SLC2A3 significantly improved the efficacy of anti‐PD‐1 mAbs (Figure 8A–D) and enhanced the function of CD8^+^ T cells (Figure 8E–H; Figure S8A–D). Micro‐CT analysis of the tibias of combination‐treated mice compared with those of vehicle‐treated and single‐strategy‐treated mice revealed decreased bone resorption in the combination group (Figure 8I; Figure S8E–G). Similar to SLC2A3 genetic or pharmacological (PS VII) inhibition, low‐glucose or low‐LA conditions synergize with anti‐PD‐1 blockade (Figure 8J–M). These data suggest that targeting SLC2A3 alters LA metabolism to promote CD8^+^ T‐cell infiltration and cytotoxicity in lung cancer bone metastasis immunotherapy.

The culture of PDOs of the original tumor epithelium en bloc with endogenous immune stroma enables investigations to facilitate anti‐PD‐1 and/or anti‐PD‐L1 immunotherapy testing. Interleukin‐2 (IL‐2) preserves intraorganoid PDO CD3^+^ TIL clusters and CD4^+^ and CD8^+^ subsets [61]. The antitumor effects of individual and combination treatments were also tested using organoids derived from patients with lung cancer bone metastasis that were supplemented with supported with IL‐2. Confocal images revealed that the combination treatment induced profound cell death in the PDOs relative to the vehicle and individual treatments (Figure 8N; Figure S8H). These data demonstrate that PS VII increases the sensitivity of lung cancer bone metastasis organoids to anti‐PD‐1 treatment.

## 3 Discussion

Lung cancer is the leading cause of cancer‐related death, and bone is one of the most common sites of metastasis in lung cancer [41]. Metabolic communication between cancer cells and immune cells within the TME plays a critical role in shaping TME dynamics [62, 63]. Our study demonstrated that SLC2A3‐mediated lactate metabolism drives lung cancer bone metastasis through metabolic reprogramming of tumor cells and immune microenvironment remodeling. We found that SLC2A3‐mediated lactate production induces p53 lactylation at K120 in bone metastatic tumor cells, effectively suppressing the transcriptional activity and tumor‐suppressive function of p53, which ultimately promotes tumor cell proliferation and metastasis. Furthermore, lactate accumulation in the TME stimulates osteoclast‐mediated bone resorption. Notably, genetic blockade of SLC2A3 in bone metastatic tumor cells induces PD‐1 expression in CD8^+^ T cells in the TME through p53 lactylation‐dependent epigenetic regulation, suggesting the potential for targeting the SLC2A3/lactate/p53 lactylation axis to strengthen immune checkpoint blockade (ICB) therapy for lung cancer bone metastasis.

Glucose serves as the primary energy source for tumor proliferation and metastasis [64, 65]. In our study, SLC2A3‐mediated glucose uptake increased the cellular glycolytic capacity, thereby promoting lung cancer bone metastasis. Compared with other GLUTs, the glucose transporter SLC2A3, a member of the SLCA2 family, takes up glucose with higher affinity and greater transport capacity [66]. We revealed that SLC2A3 is selectively overexpressed in bone metastatic tumors and that its expression is significantly negatively correlated with clinical outcomes in multiple patient cohorts. Furthermore, SLC2A3 knockdown significantly inhibited lung cancer bone metastasis both in vitro and in vivo. These findings suggest that SLC2A3 may be a novel therapeutic target for lung cancer bone metastasis.

Lactate, an abundant component produced from glycolytic flux in tumor cells [15], has been associated with tumorigenesis and multiple biological functions, including its role as a precursor for lysine lactylation, a novel epigenetic modification that directly stimulates gene transcription [67, 68, 69]. The tumor suppressor p53, a transcription factor, prevents malignant transformation by regulating diverse cellular processes, including DNA repair, cell cycle progression, and survival under stress [70, 71]. Several studies have shown that lactylation of p53 at K120 and K139 within its DNA‐binding domain might dampen its tumor‐suppressive function [47]. Our data revealed that SLC2A3 knockdown significantly reduced both cellular lactate production and pan‐lactylation in bone metastatic tumor cells. Further investigation demonstrated that SLC2A3 modulates the p53 signaling pathway in bone metastatic tumor cells by enhancing p53 K120 lactylation, which antagonizes acetylation at the same residue, thereby suppressing p53 transcriptional activity. As a result, our findings establish an SLC2A3/lactate/p53 lactylation axis in bone metastatic tumors, which may constitute a novel metabolic‒epigenetic mechanism driving metastatic progression.

In response to oncogenic signals from growing tumors, the TME undergoes continuous remodeling during cancer progression [72]. Bone metastasis is a complex process characterized by immune microenvironment remodeling and a disrupted balance between bone formation and resorption [73, 74]. Recent reports suggest the role of lactate efflux in maintaining an acidic TME, thereby promoting angiogenesis, cell invasion, and metastasis [75]. Elevated lactate levels in the TME inhibit monocyte differentiation into dendritic cells, reduce cytokine production, and diminish cytotoxic activity by T cells and NK cells, collectively contributing to immune evasion by cancer cells [76, 77]. Furthermore, increased lactate was shown to be positively related to osteoclast‐mediated bone resorption in vitro [78]. Our single‐cell analysis revealed a significant reduction in CD8^+^ T‐cell infiltration and increased osteoclast abundance in bone metastases compared with those in primary lung cancer. Notably, our further investigation revealed that while SLC2A3‐mediated lactate production potently stimulated bone resorption, SLC2A3 knockdown had a minimal effect on activated CD8^+^ T cells in the TME. Emerging evidence indicates that p53 acetylation at K120/164 is essential for p53‐dependent PD‐1 transcriptional regulation [59]. Our data revealed that reducing bone metastatic tumor cell‐derived SLC2A3‐mediated lactate production in the TME attenuates p53 K120 lactylation while promoting K120 acetylation in CD8^+^ T cells, ultimately leading to increased PD‐1 expression. Therefore, SLC2A3‐mediated lactate accumulation in the TME promotes immune evasion by inducing PD‐1 expression in CD8^+^ T cells, thereby suppressing their antitumor activity. A recent study revealed that SLC2A3 is highly expressed in tumor‐infiltrating immune cells [79], suggesting that CD8^+^ T cells may increase glucose uptake through SLC2A3, leading to increased PD‐1 expression. Future studies should explore the metabolic communication between bone metastatic tumor cells and CD8^+^ T cells in the TME mediated by SLC2A3‐dependent lactate production and develop more targeted therapeutic strategies for immune evasion.

Accumulating evidence shows that targeted agents are effective at preventing host cells from reacting to tumor products, making them valuable additional approaches to conventional treatments for bone metastasis [80, 81]. As metabolic adaptation mechanisms in cancer cells have been observed in situ in patients with bone metastasis [82], SLC2A3, as a key glucose transporter, is a promising therapeutic target for lung cancer bone metastasis. Here, we identified an SLC2A3 inhibitor, Paris saponin VII, through structure‐based molecular design. Previous studies indicate that PS VII targets RORα to regulate glycolysis, which aligns with our findings [83]. Any potential off‐target effects of this natural compound remain within glucose metabolic pathways and do not conflict with the core mechanism we report. As a natural product, PS VII's multi‐target characteristics may indeed contribute to its anti‐tumor efficacy, though developing more specific derivatives represents an important direction. Furthermore, we demonstrated that PS VII could improve the therapeutic efficacy of anti‐PD‐1 treatment for lung cancer bone metastasis through multiple preclinical models. Therefore, as SLC2A3 inhibition upregulates PD‐1 expression in tumor‐infiltrating CD8^+^ T cells, our data clearly illustrate that targeting SLC2A3 may increase the efficacy of ICBs in the treatment of lung cancer bone metastasis.

In conclusion, our results establish the SLC2A3/lactate/p53 lactylation axis as a key driver of tumor progression and immune evasion in lung cancer bone metastasis. This immunometabolic mechanism provides a potential clinical treatment strategy, either through SLC2A3‐targeted monotherapy or in combination with PD‐1 blockade, for managing lung cancer bone metastasis.

## 4 Methods

### 4.1 Data and Code Availability

The oligonucleotide sequences used in this study are provided in Table S1. The proteomic data of primary lung cancer tissue vs. bone metastatic lung cancer tissue are provided in Table S2. The proteomic data (L6 cells vs. L6shSLC2A3 cells; L6 cells vs. L6 cells treatment with Paris saponin VII) were provided as Table S3. Non‐Targeted Metabolomics data are provided in Table S4. The RNA‐seq data (Osteoclast vs. Osteoclast‐LA) were provided as Table S6. In addition, all data are available upon request. No code was developed for this study.

### 4.2 Cell Lines

The NSCLC cell line A549 (L0) and derived metastatic clones (L2, L6) were obtained from professor Luo Jian (Tongji University School of Medicine, China). L0, L2, L6, and mouse‐derived lung cancer cell line CMT167 were grown in DMEM with 10% fetal bovine serum and 100 mg/mL penicillin/streptomycin. NCI‐H441 (H441), NCI‐H460 (H460) cells were grown in RPMI1640 with 10% FBS and 100 mg/mL penicillin/streptomycin. All cells were maintained at 37°C with 5% CO_2_ in a cell incubator.

### 4.3 Detection of Cellular Glycolysis Rate

Glycolysis rate was measured using the Seahorse XF glycolysis rate assay kit (cat. no. 103344, Agilent) according to the manufacturer's instructions. Briefly, cells were seeded on Seahorse XF‐96 plates at a density of 1×10^5^ cells/well. Before glycolytic Proton Efflux Rate (glycoPER) measurements, NRCM culture medium was replaced with Seahorse XF glycolysis rate assay solution containing glucose (cat. no. 103577, Agilent), glutamine (cat. no. 103579, Agilent), sodium pyruvate (cat. no. 103578, Agilent), and HEPES buffer, and incubated in a 37°C non‐CO_2_ incubator for 1 h. The glycoPER was measured at baseline and after sequential treatment with 0.5 µM Rot/AA (Rotenone/Antimicrobial A, mitochondrial electron transport chain inhibitors) and 50 µM 2‐DG (2‐deoxy D‐glucose) on a Seahorse XF flux analyzer 96. Experimental data were analyzed using the Agilent Seahorse Glycolysis Rate Assay report generator.

### 4.4 Western Blotting

Cultured cells were lysed with RIPA lysis buffer (20 mM Tris‐HCl pH 7.6, 150 mM NaCl, 1% NP‐40 detergent, 1% sodium deoxycholate, 0.1% SDS) with phosphatase and protease inhibitors (1:100, cat. no. A32959; Thermo Fisher), followed by centrifugation at 12, 000 × g for 15 min. The protein concentration was measured with a BCA Protein Assay Kit. Proteins (20 µg) were separated by 10%–12% SDS‐PAGE gel, placed onto polyvinylidene fluoride (PVDF) membranes, and blocked with 5% nonfat dry milk for the duration of 1 h. Primary antibodies were incubated at 4°C overnight. Secondary antibodies were incubated for one hour at room temperature. Images were captured by Odyssey software (Li‐Cor). Antibodies used were β‐actin (1:5, 000 dilution, cat. no. 30101, Yeasen), β‐tublin (1:5, 000 dilution, cat. no. M20005, Abmart), Bax (1:2, 000 dilution, cat. no. 2772, Cell Signaling Technology), Cleaved PARP (1:2, 000 dilution, cat. no. 5625, Cell Signaling Technology), CDK4 (1:2, 000 dilution, cat. no. 12790, Cell Signaling Technology), Puma (1:2, 000 dilution, cat. no. 4976, Cell Signaling Technology), p21 (1:2, 000 dilution, cat. no. 2947, Cell Signaling Technology), Cleaved Caspase 3 (1:2, 000 dilution, cat. no. 25128‐1‐AP, Proteintech), E‐cadherin (1:2, 000 dilution, cat. no. 20874‐1‐AP, Proteintech), N‐cadherin (1:2, 000 dilution, cat. no. 22018‐1‐AP, Proteintech), p53 (1:2, 000 dilution, cat. no. 10442‐1‐AP, Proteintech), p53 (acetyl K120) (1:2, 000 dilution, cat. no. ab78316, Abcam), Pan Lactylated‐Lysine (1:1, 000 dilution, cat. no. SHBP0618, SHANGHAI BIOPROFILE), SLC2A3 (1:2, 000 dilution, cat. no. ABHB‐19, Boster), GAPDH (1:10, 000 dilution, cat. no. A19056, ABclonal), MCT1 (1:1, 000 dilution, cat. no. YN0868, Immunoway), MCT4 (1:1, 000 dilution, cat. no. YT2685, Immunoway), SLC2A4 (1:1, 000 dilution, cat. no. YM9019, Immunoway), MAPK13 (1:1, 000 dilution, cat. no. YN1615, Immunoway), p‐MAPK13(1:1, 000 dilution, cat. no. PB9721, Boster), p38 (1:2, 000 dilution, cat. no. 8690, Cell Signaling Technology), p‐p38 (1:2, 000 dilution, cat. no. 4511, Cell Signaling Technology), p44/42 MAPK (Erk1/2) (1:2, 000 dilution, cat. no. 4695, Cell Signaling Technology), p‐p44/42 MAPK (Erk1/2) (1:2, 000 dilution, cat. no. 4370, Cell Signaling Technology), p65 (1:2, 000 dilution, cat. no. 8242, Cell Signaling Technology), p‐p65 (1:2, 000 dilution, cat. no. 3033, Cell Signaling Technology).

### 4.5 Colony Formation Assay

Cells were plated in 12‐well plates at a density of 3, 000 cells per well and treated with the indicated agents the following day, followed by culture in complete medium for 14 days. After incubation, cells were fixed with 4% paraformaldehyde (PFA) and stained with crystal violet. The stained crystal violet was dissolved in 10% acetic acid, and absorbance was measured at 595 nm.

### 4.6 Transwell Assay

The migration capacity of cancer cells was determined using a 24‐well plate, transwell chambers (Corning, USA) with an 8 µm pore size and matrigel (100 µg/ml). 5 × 10^4^ cells in 100 µl serum‐free DMEM were plated in the upper chambers, and 600 µL medium containing 10% FBS was added to the lower chambers. Incubate cells under suitable conditions for 24 h, and then cells were fixed with 4% PFA and stained with crystal violet after invading the lower side of the membrane. Finally, migrated cells were observed and counted.

### 4.7 Immunohistochemistry (IHC) Staining and Scoring Assay

Immunohistochemistry (IHC) was performed using a detection kit (cat. no. 6312ES50, Yeasen) according to the manufacturer's instructions. Briefly, paraffin sections were deparaffinized with fresh xylene and hydrated in gradient alcohol. Antigen retrieval was conducted in citric acid buffer at 100°C for 30 min, followed by the addition of an appropriate amount of endogenous peroxidase blocker. The sections were blocked with standard goat serum working solution at room temperature for 15 min, then transferred to a 4°C refrigerator and incubated overnight with SLC2A3 primary antibodies. The next day, horseradish peroxidase‐labeled streptavidin working solution was applied and incubated at room temperature for 15 min, followed by three PBS washes, 3 min each. Color development was performed using diaminobenzidine (DAB), and sections were counterstained with hematoxylin for 30 s, rinsed with tap water for 5 min, dehydrated, cleared, and mounted with neutral gum. The IHC‐stained sections were independently reviewed and scored by two senior pathologists, and a final score was calculated by ImageJ.

### 4.8 Osteoclastogenesis Assay

Mononuclear cells were isolated from mouse bone marrow and plated in culture dishes, then cultured in α‐MEM complete medium supplemented with M‐CSF (50 ng/mL) and RANKL (100 ng/mL). After osteoclast formation, we performed a TRAP assay using a TRAP staining kit (cat. no. 387A, Sigma–Aldrich) following the manufacturer's instructions. The multinucleated TRAP‐positive cells (mature osteoclasts) were monitored by a Leica microscope (Leica, Germany).

### 4.9 TRAP Staining

After treatment with a dehydration gradient, the paraffin sections were treated with 0.1% Triton X‐100 for 30 min, and then stained with TRAP staining kit (cat. no.387A, Sigma–Aldrich) at 37°C for 1 h. ImageJ was used to analyze the surface area of osteoclasts.

### 4.10 Pit Formation Assay

Mature osteoclasts were induced from primary bone marrow stromal cells by treatment with RANKL (100 ng/mL) for 4 days, gently detached using versene, and subsequently seeded onto bovine bone slices. Following 48 h of culture in medium supplemented with M‐CSF (50 ng/mL) and RANKL (100 ng/mL), the bone slices were fixed, exposed to ammonium hydroxide to remove residual cells, stained with toluidine blue, and thoroughly washed. Resorption pits were initially visualized under a light microscope and further characterized morphologically through imaging and quantification using two‐photon laser confocal microscopy (Leica TCS SP8).

### 4.11 Chromatin Immunoprecipitation (ChIP) Assay

Cells were fixed with 1% formaldehyde for 10 min at room temperature, followed by a brief wash with cold PBS. The fixed cells were then lysed in ChIP lysis buffer on ice for 10 min. After sonication, lysates were centrifuged at 8, 000 rpm for 5 min at 4°C to collect the supernatant. For immunoprecipitation, p53 antibody or control IgG was pre‐incubated with protein A/G beads for 6 h at 4°C. The cleared lysates were then added and incubated with the bead‐antibody complexes overnight at 4°C. The beads were sequentially washed with the following buffers: TSE I (10 mM Tris‐HCl pH 7.5, 1 mM EDTA, 0.5 M NaCl, 1% Triton X‐100, 0.1% SDS, 0.1% deoxycholate), TSE II (10 mM Tris‐HCl pH 7.5, 1 mM EDTA, 0.1% SDS, 1% Triton X‐100, 0.1% deoxycholate), Buffer III (10 mM Tris‐HCl pH 7.5, 1 mM EDTA, 0.25 M LiCl, 0.5% deoxycholate, 0.5% NP‐40), and finally Buffer TE (10 mM Tris‐HCl pH 7.5, 1 mM EDTA). Bound protein‐DNA complexes were eluted in elution buffer (0.5% SDS, 0.1 M NaHCO_3_) supplemented with 100 µg/mL RNase A and 200 µg/mL proteinase K, followed by reverse cross‐linking at 65°C for ≥6 h. DNA was purified using a PCR purification kit (cat. no. DP214‐03, TIANGEN) and analyzed by real‐time PCR to determine the relative enrichment of target proteins or modifications at specific genomic loci. The primer sequences were listed in\ Table S1.

### 4.12 Drug Affinity Responsive Target Stability Assay (DARTS)

The protein was isolated from L6 cells by using CHAPS lysis buffer pH7.4 (120 mM NaCl, 40 mM HEPES, 10 mM β‐glycerophosphate, 1 mM EDTA, 0.3% CHAPS) supplemented with phosphatase and protease inhibitors (1:100, cat. no. A32959; Thermo Fisher), and centrifuged at 12, 000 *×* g at 4°C for 15 min. The supernatant was collected. Protein concentration was quantified using the BCA assay kit (cat. no. 23227, Thermo Fisher). Samples of each group were treated with Paris saponin VII (cat. no. T4085, Target Molecule) and DMSO for 1 h. Samples were then incubated with pronase (cat. no. 53702, Merck) in different proportions for 5 min at 37°C. After stopping the reaction, the samples were detected for western blot.

### 4.13 Cellular Thermal Shift Assay (CETSA)

The protein was extracted from A549‐L6 cells by using CHAPS lysis buffer pH7.4 (120 mM NaCl, 40 mM HEPES, 10 mM β‐glycerophosphate, 1 mM EDTA, 0.3% CHAPS) supplemented with phosphatase and protease inhibitors (1:100, cat. no. A32959; Thermo Fisher). The lysate was centrifuged at 12, 000 × g for 15 min at 4°C, and the supernatant was collected. Protein concentration was quantified using the BCA assay kit (cat. no. 23227, Thermo Fisher). The supernatant was divided into two groups: (1) control (untreated) and (2) 0.25 µM Paris saponin VII, followed by incubation at room temperature for 30 min. Each group was further divided into five aliquots and subjected to thermal denaturation at increasing temperatures (48, 51, and 54°C) for 3 min, cooled for 3 min at room temperature. After stopping the reaction, the samples were analyzed by western blot.

### 4.14 Drug Semi‐Inhibitory Concentration Detection

Cell proliferation was evaluated using the CCK‐8 assay kit (cat. no. 40203ES80, Yeasen). The day before, cells were seeded in 96‐well plates in triplicate at 3, 000 cells per well and incubated at 37°C with 5% CO_2_ in cell incubator overnight. Paris saponin VII (cat. no. T4085, Target Molecule) was dissolved with DMSO and diluted in DMEM into a gradient concentration. Cells were treated with the drug for growth to an appropriate density, added 100 µL to each well, and treated with equal amounts of DMEM instead of drugs as a control group, and cultured for 48 h.Then 100 µL of free medium containing 10 µL of CCK‐8 reagent was added to each well. After incubating at 37°C for 2 h with light avoidance, absorbance at 450 nm was measured using a multifunctional microplate reader (BioTek, USA). The following formula was used: cell viability (%) = [(OD of the experimental samples/OD of the control) × 100%], and half‐maximum inhibitory concentration was determined by GraphPad Prism8.0 nonlinear regression analysis (IC_50_).

### 4.15 Immunoprecipitation

Cells were lysed with CHAPS lysis buffer pH7.4 (120 mM NaCl, 40 mM HEPES, 10 mM β‐glycerophosphate, 1 mM EDTA, 0.3% CHAPS) supplemented with phosphatase and protease inhibitors (1:100, cat. no. A32959; Thermo Fisher), incubated on ice for 5 min, sonicated for 10 min at 4°C, and then centrifuged at 12, 000 rpm at 4°C for 15 min. The cell supernatant was incubated for 12 h at 4°C with the antibodies or anti‐immunoglobulinG (IgG) (cat. no. HY‐P73904, MedChemExpress) and then incubated with prewashed protein A/G beads (cat. no. 320422, Thermo Fisher) for 3 h at 4°C, followed by washing, elution, and immunoblotted detection.

### 4.16 Streptavidin‐Biotin Affinity Pull‐Down Assay

Cells were lysed with CHAPS lysis buffer pH7.4 (120 mM NaCl, 40 mM HEPES, 10 mM β‐glycerophosphate, 1 mM EDTA, 0.3% CHAPS) supplemented with phosphatase and protease inhibitors (1:100, cat. no. A32959; Thermo Fisher), incubated on ice for 5 min, sonicated for 10 min at 4°C, and then centrifuged at 12, 000 rpm at 4°C for 15 min. The cell supernatant was incubated for 3 h at room temperature with free biotin or biotin‐Paris saponin VII (biotin‐PS VII) (Shanxi Xingbei Aike Biotechnology Co., Ltd). Subsequently, the prewashed streptavidin agarose beads (cat. no. 20349, Thermo Fisher) were added to the system as above and incubated 1 h at room temperature with rotation. The beads were washed three times with elution buffer (200 mM NaCl, 50 mM Tris‐HCl PH7.5, 10%Glycerol, 1 mM EDTA) and then detected by western blot or IP‐MS.

### 4.17 Luciferase Reporter Assay

Promoter sequence of PD‐1 was cloned into pGL3‐basic luciferase reporter plasmid. PD‐1‐luciferase reporter together with either pCMV‐TP53(Human)‐K120R‐HA‐neo (cat. no. 411304, ShangHaiHeWu) or pCMV‐TP53(Human) ‐HA‐neo (cat. no. P54044, ShangHaiHeWu) was transfected into HEK293T cells. At 72 h after transfection, the luciferase activities in cell lysates were measured with the luciferase assay system (BMG Labtech, Germany).

### 4.18 Plasmid Construction

Plasmids and primers used in this study are summarized in Table S1. Target full‐length *SLC2A3*, cDNA was cloned into a PCDH vector using ClonExpress II One Step Cloning Kit (cat. no. C112‐01, Vazyme) and corresponding primers. A short hairpin RNA (shRNA) sequence was cloned into pLKO.1 plasmid vector. HEK293T cells were cotransfected with lentiviral plasmid DNA, pMD2.G, and psPAX2 for 48 h. Then culture medium containing lentivirus was collected and filtered with 0.45 µm PVDF membrane (Merck, Germany). Target cells were infected by virus for 12 h and further selected with puromycin (cat. no. 60210ES60, Yeasen) or blasticidin (cat. no. SBR00022, Merck). Selected cells were verified by western blotting and then used for further experiments.

### 4.19 Cell Apoptosis Assay

Cells were plated in 6‐well plates at 1 × 10^5^ cells per well and treated with the indicated agents the next day for 48 h. Cells were washed with cold PBS and resuspended in Annexin V binding buffer and stained with Annexin V and PI at room temperature by using the Annexin V Apoptosis Detection Kit (cat. no. 556547, BD Biosciences). Annexin V‐positive cells were detected using BD FACSCanto II (BD Biosciences, USA) within 30 min after staining.

### 4.20 Quantitative Real‐Time PCR

RNA extraction was using the RNA TRIzol Reagent. RNA samples were reverse‐transcribed using the Hifair II first Strand cDNA Synthesis SuperMix (cat. no. 11120ES60, Yeasen). Quantitative real‐time PCR (qPCR) was performed using Hieff qPCR SYBR Green Master Mix (cat. no. 11201ES08, Yeasen) according to the manufacturer's instructions. Data were generated using the comparative threshold cycle (ΔCt) method with normalization to the reference gene *18S*. The primer sequences were listed Table S1.

### 4.21 Patient‐Derived Organoid (PDO) Assay

Tumor tissues derived from surgical resections were cut into small pieces finely on ice, and enzymatically digested using collagenase type I (cat. no. 17100017, Gibco). Cells were further disassociated with shaking for 30 min at 37°C until the solution was turbid, ground, and filtered with 100 µm filtering. The resulting cells were centrifuged with 1500 rpm for 5 min and keep the cell precipitate. Red blood cells were lysed with RBC lysis buffer (cat. no. 00‐4333‐58, Thermo Fisher) for 2 min on ice and then centrifuged with 1500 rpm for 5 min. The resulting cells were washed with PBS containing 10% FBS and 2% penicillin/streptomycin. Then the number of cells was then calculated and embedded in Matrigel (cat. no. 354248, Corning). After solidification for 30 min at 37°C, cells were overlaid with human breast cancer organoid medium. In vitro organoid killing assay was performed as the size of organoids reached 30–100 µm. Calcein‐AM/PI (cat. no. 92210, Merck) was used to measure the survival rate of organoids. Fluorescence images were taken and analyzed with ImageJ.

### 4.22 Micro‐CT Analysis

The femurs and tibias were isolated from mice, muscle tissue was removed, and then fixed with 4% polyformaldehyde for two days. The bones were scanned by Micro‐CT (Hiscan XM, China) with 60 kV and 166 µA and using a detection pixel size of 9 µm. Hiscan Analyzer V3.0 was used to reconstruct and analyze the scanned images respectively. Reconstructed images were used to analyze the trabecular bone of the vertebra and the tibia, quantify morphometric indices, including bone volume fraction (BV/TV), bone mineral density (BMD) and trabecular number (Tb.N).

### 4.23 CD8^+^ T Cell Isolation and Stimulation

Peripheral CD8^+^ T cells were isolated from mouse spleens by MojoSort Mouse CD8^+^ T Cell Isolation Kit (480008, BioLegend). The cells were then stimulated with plate‐bound anti‐CD3 (1 µg/mL) (cat. no. 567115, BD Pharmingen) and anti‐CD28 (1 µg/mL) (cat. no. 567110, BD Pharmingen) antibodies, followed by culturing with complete RPMI 1640 medium containing 10% FBS and IL2 (10 ng /mL) (cat. no. 550069, BD Pharmingen) in 96‐well plates.

### 4.24 Mouse Experiments

Our study exclusively examined male mice. It is unknown whether the findings are relevant to female mice. Male BALB/c nude mice and C57BL/6 mice aged 6 weeks were used in this study, and they were provided by East China Normal University purchased from Jihui Animal Feeding Co, Ltd (Shanghai, China). All animal protocols were approved by East China Normal University and were performed in accordance with the guidelines of the Ethics Committee of East China Normal University. Mice were caged in groups of five in a laminar air flow cabinet under specific pathogen‐free conditions. They were fed with abundant food and water, and kept on a 12 h light/dark cycle. L6 cells expressing shSLC2A3 or scrambled shRNA were injected into the left ventricle or tibia of the BALB/c nude mice for the lung cancer model. Nude mice were injected with 1 × 10^5^ cancer cells at per mouse. CMT167 cells expressing shSLC2A3 or scrambled shRNA were injected into the left ventricle or tibia of the C57BL/6 mice. C57BL/6 mice were injected with 5 × 10^4^ cancer cells per mouse. For LA treatment, mice were injected with 100 µL of 0.2 mM or 1 mM LA three times per week via intraperitoneal injection (i.p.) after cancer cells were injected for 12 or 15 days since day 3. For anti‐PD‐1 antibody (cat. no. BP0146, BioXcell) treatment, mice were i.p. injected with 100 µL of 50 µg anti‐PD‐1 three times per week after cancer cells were injected for 12 or 21 days since day 3 or 7. For Paris saponin VII (cat. no. T4085, Target Molecule) treatment, mice were i.p. injected with Paris saponin VII (2 mg/kg) three times per week after cancer cells were injected for 12 or 21 days since day 3 or 7. For glucose (cat. no. ST1227, Beyotime Biotechnology) uptake experiments, mice were treated with glucose added to their drinking water (1% glucose or 10% glucose) after cancer cells were injected for 12 or 28 days since day 3 or 7. Then live‐animal images were acquired with IVIS Spectrum CT (PerkinElmer).

### 4.25 Proteomic Analysis

Frozen tumor tissues were lysed with lysis buffer (8 M Urea, 100 mM Tris‐HCl pH8.0) supplemented with phosphatase and protease inhibitors (1:100, cat. no. A32959; Thermo Fisher), incubated on ice for 15 min, homogenized for 2 min with 70 Hz, sonicated for 10 min at 4°C, and then centrifuged at 12, 000 × g at 4°C for 15 min. The supernatant protein concentration was quantified using BCA assay kit, then 250 µg of protein was reduced with 10 mM of Dithiothreitol (DTT) (cat. no. R0861, Thermo Fisher) in 55°C for 30 min. After cooled to room temperature, the protein was alkylated with iodoacetamide (IAM, 15 mM) (cat. no. 35603, Thermo Fisher) in the dark for 30 min. The buffer was exchanged to 0.1 M TEAB (cat. no. T7408, MERCK) by overnight precipitation with acetone. Samples were digested overnight at 37°C with trypsin (cat. no. V5111, Promega), desalted, and labeled by a 10‐plex tandem mass tag (TMT) labeling reagent (cat. no. 90110, Thermo Fisher) following the manufacturer's instructions. The remaining steps of offline fractionation and LC‐MS/MS analysis were essentially the same as previously described [84].

For cell pellets, each sample was lysed in 8 M urea buffer with protease and phosphatase inhibitors on ice for 30 min. incubated on ice for 5 min, sonicated for 10 min at 4°C, and then centrifuged at 12, 000 rpm at 4°C for 15 min. Protein concentration of the supernatant was quantified using the BCA assay kit, then protein (30 µg) was reduced with dithiothreitol (DTT, 10 mM) at 55°C for 30 min. After cooled to room temperature, the protein was alkylated with iodoacetamide (IAM, 15 mM) in the dark for 30 min. Afterwards, four volumes of 50 mM NH_4_HCO_3_ solution were added. Samples were digested at 37°C with trypsin (1:100) overnight. Finally, 1% final concentration of formic acid was added to terminate the enzymatic reaction. The samples were then desalted, dried, and resuspended in buffer A (2% ACN, 0.1% formic acid) for LC‐MS/MS. For each sample, peptides were subjected to chromatographic separation using a Vanquish Neo UHPLC system (Thermo Fisher, USA). The buffers were as follows: Solvent A was 0.1% formic acid in water, and Solvent B was 0.1% formic acid in 80% acetonitrile (in water). The chromatographic column was equilibrated with 96% Solvent A. Samples were injected onto a trap column (PepMap Neo 5 µm C18, 300 µm × 5 mm, Thermo Fisher, USA) and then separated by an analytical column (µPAC Neo High Throughput column, Thermo Fisher, USA) using the following linear gradient program: 4%–6% phase B for 0.1 min, 6%–12% phase B for 1 min, 12%–22.5% phase B for 3.2 min, 22.5%–45% phase B for 1.8 min, 99% phase B for 1.9 min. After peptide separation, Data Independent Acquisition (DIA) mass spectrometry analysis was performed using an Orbitrap Astral mass spectrometer (Thermo Fisher, USA) with an 8‐min analysis duration. The electrospray voltage was set to 2.2 kV in positive ion mode. The precursor scan range was 380–980 m/z, with MS1 resolution at 240, 000, AGC target at 500%, and maximum injection time (IT) at 3 ms. For MS2, the resolution was 80, 000, AGC target 500%, maximum IT 3 ms, RF‐lens 40%, activation type HCD, isolation window 2 Th, normalized collision energy 25%, and cycle time 0.6 s. The RAW data files were analyzed using DIA‐NN software.

### 4.26 10× Genomics Library Preparation and Single‐Cell RNA Sequencing

Fresh tumor specimens from lung cancer patients were cut into approximately 1 mm^3^ fragments in RPMI‐1640 medium supplemented with 10% FBS. Tissue dissociation was performed using the Tumor Dissociation Kit (human) following the manufacturer's protocol. After filtering using the 70 µm cell strainer in RPMI‐ 1640 medium, the suspended cells were centrifuged at 500 × g for 5 min. The cell pellets were washed with PBS and resuspended in sorting buffer (PBS with 2% FBS). The cell suspension was diluted to the optimal density recommended by 10× Genomics Chromium single‐cell processing and library preparation. According to the standard pipeline and default parameters, CellRanger v3.1.0 was used to process the data, map sequences to the homo genome (mm10), and compile unique molecular identifier (UMI) counts within feature‐barcode matrices. The resulting matrix was normalized through a global scaling strategy, transformed using a scaling factor, and subjected to log transformation via the “LogNormalize” function in Seurat v4.4.0 to facilitate downstream analyses.

### 4.27 Flow Cytometry

Single‐cell suspensions from CMT167 tumors were carried out using a tumor lysis buffer with 10 U/mL Collagenase type I (cat. no. 17100017, Gibco), 100 U/mL Collagenase type IV (cat. no. 17104019, Gibco), 1 mg/ml DNase I (cat. no. 10104159001, Sigma–Aldrich) and HBSS (cat. no. PB180324, Pricella) and incubated for 45 min at 37°C. Following enzymatic digestion, the tissues were passed through a 70 µm filter (Merck, Germany) and were treated with a red blood cell lysis buffer (cat. no. C3702, Beyotime). For cell surface molecules detection, a single cell suspension in PBS supplemented 2% FBS was stained with the indicated antibodies at room temperature for 30 min. For intracellular staining, cells were first stained with surface markers at 4°C for 30 min and then fixed with fixation/permeabilization buffer in the dark and at room temperature for 1 h, followed by washing twice with permeabilization buffer (cat. no. 421002, Biolegend) according to the manufacturer's instructions. For intracelluar cytokine detection, cells were fixed with 4% PFA and permeabilized using Perm Wash Buffer. Stained cells were further analyzed using flow cytometry (BD Fortessa, USA), and data were processed with Flowjo software. Antibodies were used as follows: CD45 (cat. no. 103132, BioLegend), CD3ε (cat. no. 100306, BioLegend), CD4 (cat. no. 100421, BioLegend), CD8a (cat. no. 100712, BioLegend), CD11b (cat. no. 101205, BioLegend), CD80 (cat. no. 104733, BioLegend), CD206 (cat. no. 141705, BioLegend), CD11c (cat. no. 117333, BioLegend), F4/80 (cat. no. 123116, BioLegend), IFN‐γ (cat. no. 505808, BioLegend), L/D (cat. no. 565388, BD Biosciences), PD‐1(cat. no. 135217, BioLegend), MHCII (cat. no. 107608, BioLegend).

### 4.28 Pharmacokinetics and Toxicity Studies of PS VII in Mice

6‐week‐old ICR Outbred Mice (ICR) were used in pharmacokinetics study. PS VII was injected via tail—vein injection with 1 mg/kg and intraperitoneal with 10 mg/kg, respectively. After injecting the inhibitor, 0.05 mL of blood was collected from the orbital area at 5 min, 15 min, 30 min, 1 h, 2 h, 4 h, 6 h, 8 h, and 24 h; The concentration of PS VII in mouse plasma samples was determined by LC‐MS/MS, and the pharmacokinetic parameters were calculated using WinNolin software. Absolute bioavailability calculation formula: F (%) = (Dose_iv_ × AUC_oral (0‐∞)_) / (Dose_oral_ × AUC_iv (0‐∞)_) × 100%. PS VII was intraperitoneal injection at 3‐days intervals for a total of 14 days to ICR mice. Clinical signs, body weight, food consumption were monitored throughout the study. Blood collection from the eye socket was performed on the day after the last administration. The details of the data are in Table S7.

### 4.29 Statistics

Statistical analysis was performed using GraphPad Prism 10.0 (GraphPad Software). Two‐tailed unpaired Student's *t* tests and Pearson's correlation coefficient assay were performed as indicated. For comparison of multiple groups, one‐way ANOVA with Tukey's multiple comparison test was used. A log‐rank test was used for survival analysis. Two‐way ANOVA was performed to compare continuous outcomes across multiple experimental groups. Survival curves were analyzed by the log‐rank (Mantel‐Cox) test. A *p* value of less than 0.05 was considered significant. *P* values were denoted as follows: ^*^ *p* < 0.05, ^**^ *p* < 0.01, ^***^ *p* < 0.001.

### 4.30 Study Approval

All animal treatments were performed according to the Guide for the Care and Use of Laboratory Animals (National Academies Press, 2011). All animal protocols were approved by the East China Normal University (m20250605). The clinical lung cancer and lung cancer bone metastasis samples were approved by the FUSCC Ethics Committee (050432‐4‐2108*). Written informed consent was obtained from all participants prior to their inclusion in this study.

## Author Contributions

L.L. and K.L. conceived and supervised this study. Y.D., Y.T., W.R., and X.H. performed the experiments and provided helpful discussions. M.L. and C. C. analyzed and interpreted the data. Y.D., Y.T., W.R., B.L., Y. L., W.Y., L.L., and K.L. wrote the manuscript. All authors reviewed and edited the manuscript. Y.D., Y.T., W.R., and X.H. contributed equally to this work. The order of co‐first authors was based on their contributions.

## Conflicts of Interest

The authors declare no conflicts of interest.

## Supporting information




**Supporting File 1**: advs74072‐sup‐0001‐SuppMat.pdf.

**Supporting File 2**: advs74072‐sup‐0002‐SuppMat.pdf.

## Data Availability

The data that support the findings of this study are available in the supplementary material of this article.

## References

[1] F. Bray , M. Laversanne , H. Sung , et al., “Global Cancer Statistics 2022: GLOBOCAN Estimates of Incidence and Mortality Worldwide for 36 Cancers in 185 Countries,” CA: A Cancer Journal for Clinicians 74, no. 3 (2024): 229–263. 38572751 10.3322/caac.21834

[2] H. H. Popper , “Progression and Metastasis of Lung Cancer,” Cancer and Metastasis Reviews 35, no. 1 (2016): 75–91, 10.1007/s10555-016-9618-0. 27018053 PMC4821869

[3] T. Sasaki , H. Kuno , T. Hiyama , et al., “2021 WHO Classification of Lung Cancer: Molecular Biology Research and Radiologic‐Pathologic Correlation,” Radiographics 44, no. 3 (2024): 230136, 10.1148/rg.230136. 38358935

[4] L. Yin , X. Liu , X. Shao , et al., “The Role of Exosomes in Lung Cancer Metastasis and Clinical Applications: an Updated Review,” Journal of Translational Medicine 19, no. 1 (2021): 312, 10.1186/s12967-021-02985-1. 34281588 PMC8287779

[5] M. Riihimäki , A. Hemminki , M. Fallah , et al., “Metastatic Sites and Survival in Lung Cancer,” Lung Cancer 86, no. 1 (2014): 78–84, 10.1016/j.lungcan.2014.07.020. 25130083

[6] M. E. Sowder and R. W. Johnson , “Bone as a Preferential Site for Metastasis,” JBMR Plus 3, no. 3 (2019): 10126. 10.1002/jbm4.10126 PMC6419612 30918918

[7] W. Zhang , I. Bado , H. Wang , H. C. Lo , and X. H. Zhang , “Bone Metastasis: Find Your Niche and Fit in,” Trends in Cancer 5, no. 2 (2019): 95–110, 10.1016/j.trecan.2018.12.004. 30755309 PMC6383208

[8] T. Ge , X. Gu , R. Jia , et al., “Crosstalk between Metabolic Reprogramming and Epigenetics in Cancer: Updates on Mechanisms and Therapeutic Opportunities,” Cancer Communications 42, no. 11 (2022): 1049–1082, 10.1002/cac2.12374. 36266736 PMC9648395

[9] D. Hanahan and R. A. Weinberg , “Hallmarks of Cancer: the Next Generation,” Cell 144, no. 5 (2011): 646–674, 10.1016/j.cell.2011.02.013. 21376230

[10] D. Hanahan , “Hallmarks of Cancer: New Dimensions,” Cancer Discovery 12, no. 1 (2022): 31–46, 10.1158/2159-8290.CD-21-1059. 35022204

[11] M. V. Liberti and J. W. Locasale , “The Warburg Effect: How Does It Benefit Cancer Cells?,” Trends in Biochemical Sciences 41, no. 3 (2016): 211–218, 10.1016/j.tibs.2015.12.001. 26778478 PMC4783224

[12] Y. Yang , Y. Chong , M. Chen , et al., “Targeting Lactate Dehydrogenase a Improves Radiotherapy Efficacy in Non‐small Cell Lung Cancer: from Bedside to Bench,” Journal of Translational Medicine 19, no. 1 (2021): 170, 10.1186/s12967-021-02825-2. 33902615 PMC8074241

[13] Y. Qian , A. Galan‐Cobo , I. Guijarro , et al., “MCT4‐dependent Lactate Secretion Suppresses Antitumor Immunity in LKB1‐deficient Lung Adenocarcinoma,” Cancer Cell 41, no. 7 (2023): 1363–1380, 10.1016/j.ccell.2023.05.015. 37327788 PMC11161201

[14] J. Feng , H. Yang , Y. Zhang , et al., “Tumor Cell‐derived Lactate Induces TAZ‐dependent Upregulation of PD‐L1 through GPR81 in human Lung Cancer Cells,” Oncogene 36, no. 42 (2017): 5829–5839, 10.1038/onc.2017.188. 28604752

[15] D. Zhang , Z. Tang , H. Huang , et al., “Metabolic Regulation of Gene Expression by Histone Lactylation,” Nature 574, no. 7779 (2019): 575–580, 10.1038/s41586-019-1678-1. 31645732 PMC6818755

[16] Z. Yang , C. Yan , J. Ma , et al., “Lactylome Analysis Suggests Lactylation‐dependent Mechanisms of Metabolic Adaptation in Hepatocellular Carcinoma,” Nature Metabolism 5, no. 1 (2023): 61–79, 10.1038/s42255-022-00710-w. 36593272

[17] A. Rizwan , I. Serganova , R. Khanin , et al., “Relationships between LDH‐A, Lactate, and Metastases in 4T1 Breast Tumors,” Clinical Cancer Research 19, no. 18 (2013): 5158–5169, 10.1158/1078-0432.CCR-12-3300. 23833310 PMC3877680

[18] J. Jiang , D. Huang , Y. Jiang , et al., “Lactate Modulates Cellular Metabolism through Histone Lactylation‐Mediated Gene Expression in Non‐Small Cell Lung Cancer,” Frontiers in Oncology 11 (2021): 647559, 10.3389/fonc.2021.647559. 34150616 PMC8208031

[19] J. R. Doherty and J. L. Cleveland , “Targeting Lactate Metabolism for Cancer Therapeutics,” Journal of Clinical Investigation 123, no. 9 (2013): 3685–3692, 10.1172/JCI69741. 23999443 PMC3754272

[20] X. Lv , Y. Lv , and X. Dai , “Lactate, Histone Lactylation and Cancer Hallmarks,” Expert Reviews in Molecular Medicine 25 (2023): 7, 10.1017/erm.2022.42. 36621008

[21] H. Wu , Y. Wang , M. Ying , C. Jin , J. Li , and X. Hu , “Lactate Dehydrogenases Amplify Reactive Oxygen Species in Cancer Cells in Response to Oxidative Stimuli,” Signal Transduction and Targeted Therapy 6, no. 1 (2021): 242, 10.1038/s41392-021-00595-3. 34176927 PMC8236487

[22] G. Rai , K. R. Brimacombe , B. T. Mott , et al., “Discovery and Optimization of Potent, Cell‐Active Pyrazole‐Based Inhibitors of Lactate Dehydrogenase (LDH),” Journal of Medicinal Chemistry 60, no. 22 (2017): 9184–9204, 10.1021/acs.jmedchem.7b00941. 29120638 PMC5894102

[23] A. Le , C. R. Cooper , A. M. Gouw , et al., “Inhibition of Lactate Dehydrogenase A Induces Oxidative Stress and Inhibits Tumor Progression,” Proceedings of the National Academy of Sciences 107, no. 5 (2010): 2037–2042, 10.1073/pnas.0914433107. PMC2836706 20133848

[24] Y. W. Wu , C. L. Chik , and R. A. Knazek , “An in Vitro and in Vivo Study of Antitumor Effects of Gossypol on human SW‐13 Adrenocortical Carcinoma,” Cancer Research 49, no. 14 (1989): 3754–3758. 2736516

[25] J. Chen , D. Zhao , Y. Wang , et al., “Lactylated Apolipoprotein C‐II Induces Immunotherapy Resistance by Promoting Extracellular Lipolysis,” Advanced Science 11, no. 38 (2024): 2406333, 10.1002/advs.202406333. 38981044 PMC11481198

[26] J. Qian , Z. C. Gong , Y. N. Zhang , et al., “Lactic Acid Promotes Metastatic Niche Formation in Bone Metastasis of Colorectal Cancer,” Cell Communication and Signaling 19, no. 1 (2021): 9, 10.1186/s12964-020-00667-x. 33478523 PMC7818572

[27] C. A. Colville , M. J. Seatter , T. J. Jess , G. W. Gould , and H. M. Thomas , “Kinetic Analysis of the Liver‐type (GLUT2) and Brain‐type (GLUT3) Glucose Transporters in Xenopus Oocytes: Substrate Specificities and Effects of Transport Inhibitors,” Biochemical Journal 290, no. 3 (1993): 701–706, 10.1042/bj2900701. 8457197 PMC1132337

[28] D. Deng , P. Sun , C. Yan , et al., “Molecular Basis of Ligand Recognition and Transport by Glucose Transporters,” Nature 526, no. 7573 (2015): 391–396, 10.1038/nature14655. 26176916

[29] X. Yao , Z. He , C. Qin , et al., “SLC2A3 promotes Macrophage Infiltration by Glycolysis Reprogramming in Gastric Cancer,” Cancer Cell International 20 (2020): 503, 10.1186/s12935-020-01599-9. 33061855 PMC7552479

[30] W. Jiang , S. Xu , M. Zhao , and C. Li , “SLC2A3 promotes Head and Neck Squamous Cancer Developing through Negatively Regulating CD8+ T Cell in Tumor Microenvironment,” Scientific Reports 14, no. 1 (2024): 29458, 10.1038/s41598-024-79417-9. 39604419 PMC11603017

[31] W. Jiang , S. Xu , and P. Li , “SLC2A3 Promotes Tumor Progression Through Lactic Acid‐Promoted TGF‐β Signaling Pathway in Oral Squamous Cell Carcinoma,” PLoS ONE 19, no. 4 (2024): 0301724, 10.1371/journal.pone.0301724. PMC11020985 38625978

[32] Y. Li , H. Lei , M. Zhang , et al., “The Effect of SLC2A3 Expression on Cisplatin Resistance of Colorectal Cancer Cells,” Iran J Public Health 50, no. 12 (2021): 2576. 36317019 10.18502/ijph.v50i12.7941 PMC9577146

[33] M. Tsukioka , Y. Matsumoto , M. Noriyuki , et al., “Expression of Glucose Transporters in Epithelial Ovarian Carcinoma: Correlation with Clinical Characteristics and Tumor Angiogenesis,” Oncology Reports 18, no. 2 (2007): 361–367. 17611657

[34] M. Younes , L. V. Lechago , J. R. Somoano , M. Mosharaf , and J. Lechago , “Immunohistochemical Detection of Glut3 in human Tumors and Normal Tissues.,” Anticancer Research 17, no. 4a (1997): 2747–2750. 9252709

[35] W. Dai , Y. Xu , S. Mo , et al., “GLUT3 induced by AMPK/CREB1 Axis Is Key for Withstanding Energy Stress and Augments the Efficacy of Current Colorectal Cancer Therapies,” Signal Transduction and Targeted Therapy 5, no. 1 (2020): 177, 10.1038/s41392-020-00220-9. 32873793 PMC7463260

[36] B. Yan , X. Li , M. Peng , et al., “The YTHDC1/GLUT3/RNF183 Axis Forms a Positive Feedback Loop That Modulates Glucose Metabolism and Bladder Cancer Progression,” Experimental & Molecular Medicine 55, no. 6 (2023): 1145–1158, 10.1038/s12276-023-00997-z. 37258572 PMC10318083

[37] A. Ali , E. Levantini , C. W. Fhu , et al., “CAV1 ‐ GLUT3 signaling Is Important for Cellular Energy and Can be Targeted by Atorvastatin in Non‐Small Cell Lung Cancer,” Theranostics 9, no. 21 (2019): 6157–6174, 10.7150/thno.35805. 31534543 PMC6735519

[38] B. Zhang , Y. Li , Q. Wu , et al., “Acetylation of KLF5 Maintains EMT and Tumorigenicity to Cause Chemoresistant Bone Metastasis in Prostate Cancer,” Nature Communications 12, no. 1 (2021): 1714, 10.1038/s41467-021-21976-w. PMC7969754 33731701

[39] K. Li , B. Yang , Y. Du , et al., “The HOXC10/NOD1/ERK Axis Drives Osteolytic Bone Metastasis of Pan‐KRAS‐mutant Lung Cancer,” Bone Research 12, no. 1 (2024): 47, 10.1038/s41413-024-00350-8. 39191757 PMC11349752

[40] P. B. Ancey , C. Contat , G. Boivin , et al., “GLUT1 Expression in Tumor‐Associated Neutrophils Promotes Lung Cancer Growth and Resistance to Radiotherapy,” Cancer Research 81, no. 9 (2021): 2345–2357, 10.1158/0008-5472.CAN-20-2870. 33753374 PMC8137580

[41] Y. He , W. Luo , Y. Liu , et al., “IL‐20RB Mediates Tumoral Response to Osteoclastic Niches and Promotes Bone Metastasis of Lung Cancer,” Journal of Clinical Investigation 132, no. 20 (2022): 157917. 10.1172/JCI157917 PMC9566910 36006737

[42] Y. Zhao , J. Ning , H. Teng , et al., “Long Noncoding RNA Malat1 Protects against Osteoporosis and Bone Metastasis,” Nature Communications 15, no. 1 (2024): 2384, 10.1038/s41467-024-46602-3. PMC10944492 38493144

[43] Y. Su , Y. Luo , P. Zhang , et al., “Glucose‐Induced CRL4COP1‐p53 Axis Amplifies Glycometabolism to Drive Tumorigenesis,” Molecular Cell 83, no. 13 (2023): 2316–2331, 10.1016/j.molcel.2023.06.010. 37390815

[44] H. Li , L. Sun , P. Gao , and H. Hu , “Lactylation in Cancer: Current Understanding and Challenges,” Cancer Cell 42, no. 11 (2024): 1803–1807, 10.1016/j.ccell.2024.09.006. 39393355

[45] Y. Chen , J. Wu , L. Zhai , et al., “Metabolic Regulation of Homologous Recombination Repair by MRE11 Lactylation,” Cell 187, no. 2 (2024): 294–311, 10.1016/j.cell.2023.11.022. 38128537 PMC11725302

[46] J. Chen , Z. Huang , Y. Chen , et al., “Lactate and Lactylation in Cancer,” Signal Transduction and Targeted Therapy 10, no. 1 (2025): 38, 10.1038/s41392-024-02082-x. 39934144 PMC11814237

[47] Z. Zong , F. Xie , S. Wang , et al., “Alanyl‐tRNA Synthetase, AARS1, Is a Lactate Sensor and Lactyltransferase That Lactylates p53 and Contributes to Tumorigenesis,” Cell 187, no. 10 (2024): 2375–2392, 10.1016/j.cell.2024.04.002. 38653238

[48] T. Li , N. Kon , L. Jiang , et al., “Tumor Suppression in the Absence of p53‐Mediated Cell‐Cycle Arrest, Apoptosis, and Senescence,” Cell 149, no. 6 (2012): 1269–1283, 10.1016/j.cell.2012.04.026. 22682249 PMC3688046

[49] X. Liu , F. Rong , J. Tang , et al., “Repression of p53 Function by SIRT5‐mediated Desuccinylation at Lysine 120 in Response to DNA Damage,” Cell Death & Differentiation 29, no. 4 (2022): 722–736, 10.1038/s41418-021-00886-w. 34642466 PMC8989948

[50] J. N. Cheng , Z. Jin , C. Su , et al., “Bone Metastases Diminish Extraosseous Response to Checkpoint Blockade Immunotherapy through Osteopontin‐producing Osteoclasts,” Cancer Cell 43, no. 6 (2025): 1093–1107.e9, 10.1016/j.ccell.2025.03.036. 40280123

[51] H. Fan , Z. Xu , K. Yao , et al., “Osteoclast Cancer Cell Metabolic Cross‐talk Confers PARP Inhibitor Resistance in Bone Metastatic Breast Cancer,” Cancer Research 84, no. 3 (2024): 449–467, 10.1158/0008-5472.CAN-23-1443. 38038966

[52] W. Chu , W. Peng , Y. Lu , et al., “PRMT6 Epigenetically Drives Metabolic Switch from Fatty Acid Oxidation toward Glycolysis and Promotes Osteoclast Differentiation during Osteoporosis,” Advanced Science 11, no. 40 (2024): 2403177, 10.1002/advs.202403177. 39120025 PMC11516099

[53] H. Chen , Y. Li , H. Li , et al., “NBS1 lactylation Is Required for Efficient DNA Repair and Chemotherapy Resistance,” Nature 631, no. 8021 (2024): 663–669, 10.1038/s41586-024-07620-9. 38961290 PMC11254748

[54] W. J. Boyle , W. S. Simonet , and D. L. Lacey , “Osteoclast Differentiation and Activation,” Nature 423, no. 6937 (2003): 337–342, 10.1038/nature01658. 12748652

[55] I. Elia , J. H. Rowe , S. Johnson , et al., “Tumor Cells Dictate Anti‐tumor Immune Responses by Altering Pyruvate Utilization and Succinate Signaling in CD8+ T Cells,” Cell Metabolism 34, no. 8 (2022): 1137–1150, 10.1016/j.cmet.2022.06.008. 35820416 PMC9357162

[56] A. Llibre , S. Kucuk , A. Gope , M. Certo , and C. Mauro , “Lactate: a Key Regulator of the Immune Response,” Immunity 58, no. 3 (2025): 535–554, 10.1016/j.immuni.2025.02.008. 40073846

[57] Y. Ping , J. Shan , H. Qin , et al., “PD‐1 Signaling Limits Expression of Phospholipid Phosphatase 1 and Promotes Intratumoral CD8+ T Cell Ferroptosis,” Immunity 57, no. 9 (2024): 2122–2139, 10.1016/j.immuni.2024.08.003. 39208806

[58] S. Kumagai , S. Koyama , K. Itahashi , et al., “Lactic Acid Promotes PD‐1 Expression in Regulatory T Cells in Highly Glycolytic Tumor Microenvironments,” Cancer Cell 40, no. 2 (2022): 201–218, 10.1016/j.ccell.2022.01.001. 35090594

[59] Z. Cao , N. Kon , Y. Liu , et al., “An Unexpected Role for p53 in Regulating Cancer Cell–intrinsic PD‐1 by Acetylation,” Science Advances 7, no. 14 (2021): abf4148. 10.1126/sciadv.abf4148 PMC8011965 33789902

[60] N. Wang , S. Zhang , Y. Yuan , et al., “Molecular Basis for Inhibiting human Glucose Transporters by Exofacial Inhibitors,” Nature Communications 13, no. 1 (2022): 2632, 10.1038/s41467-022-30326-3. PMC9098912 35552392

[61] J. T. Neal , X. Li , J. Zhu , et al., “Organoid Modeling of the Tumor Immune Microenvironment,” Cell 175, no. 7 (2018): 1972–1988, 10.1016/j.cell.2018.11.021. 30550791 PMC6656687

[62] A. Del Conte , E. De Carlo , E. Bertoli , et al., “Bone Metastasis and Immune Checkpoint Inhibitors in Non‐Small Cell Lung Cancer (NSCLC): Microenvironment and Possible Clinical Implications,” International Journal of Molecular Sciences 23, no. 12 (2022): 6832. 35743275 10.3390/ijms23126832 PMC9224636

[63] Y. Zhu , Z. Zhou , X. Du , et al., “Cancer Cell‐derived Arginine Fuels Polyamine Biosynthesis in Tumor‐associated Macrophages to Promote Immune Evasion,” Cancer Cell 43, no. 6 (2025): 1045–1060, 10.1016/j.ccell.2025.03.015. 40185095

[64] Q. Shi , Q. Shen , Y. Liu , et al., “Increased Glucose Metabolism in TAMs Fuels O‐GlcNAcylation of Lysosomal Cathepsin B to Promote Cancer Metastasis and Chemoresistance,” Cancer Cell 40, no. 10 (2022): 1207–1222, 10.1016/j.ccell.2022.08.012. 36084651

[65] D. Sharma , M. Singh , and R. Rani , “Role of LDH in Tumor Glycolysis: Regulation of LDHA by Small Molecules for Cancer Therapeutics,” Seminars in Cancer Biology 87 (2022): 184–195, 10.1016/j.semcancer.2022.11.007. 36371026

[66] W. Peng , C. Tan , L. Mo , et al., “Glucose Transporter 3 in Neuronal Glucose Metabolism: Health and Diseases,” Metabolism 123 (2021): 154869, 10.1016/j.metabol.2021.154869. 34425073

[67] W. Li , C. Zhou , L. Yu , et al., “Tumor‐derived Lactate Promotes Resistance to Bevacizumab Treatment by Facilitating Autophagy Enhancer Protein RUBCNL Expression through Histone H3 Lysine 18 Lactylation (H3K18la) in Colorectal Cancer,” Autophagy 20, no. 1 (2024): 114–130, 10.1080/15548627.2023.2249762. 37615625 PMC10761097

[68] J. Yu , P. Chai , M. Xie , et al., “Histone Lactylation Drives Oncogenesis by Facilitating m6A Reader Protein YTHDF2 Expression in Ocular Melanoma,” Genome Biology 22, no. 1 (2021): 85, 10.1186/s13059-021-02308-z. 33726814 PMC7962360

[69] J. Yang , L. Luo , C. Zhao , et al., “A Positive Feedback Loop between Inactive VHL‐Triggered Histone Lactylation and PDGFRβ Signaling Drives Clear Cell Renal Cell Carcinoma Progression,” International Journal of Biological Sciences 18, no. 8 (2022): 3470–3483, 10.7150/ijbs.73398. 35637958 PMC9134910

[70] D. P. Lane , “p53, guardian of the Genome,” Nature 358, no. 6381 (1992): 15–16, 10.1038/358015a0. 1614522

[71] K. H. Vousden and X. Lu , “Live or Let Die: the Cell's Response to p53,” Nature Reviews Cancer 2, no. 8 (2002): 594–604, 10.1038/nrc864. 12154352

[72] D. F. Quail and J. A. Joyce , “Microenvironmental Regulation of Tumor Progression and Metastasis,” Nature Medicine 19, no. 11 (2013): 1423–1437, 10.1038/nm.3394. PMC3954707 24202395

[73] S. Jin , J. Gao , R. Yang , et al., “A Baicalin‐Loaded Coaxial Nanofiber Scaffold Regulated Inflammation and Osteoclast Differentiation for Vascularized Bone Regeneration,” Bioactive Materials 8 (2022): 559–572. 34541420 10.1016/j.bioactmat.2021.06.028 PMC8436066

[74] T. de Jong , A. D. Bakker , V. Everts , and T. H. Smit , “The Intricate Anatomy of the Periodontal Ligament and Its Development: Lessons for Periodontal Regeneration,” Journal of Periodontal Research 52, no. 6 (2017): 965–974, 10.1111/jre.12477. 28635007

[75] K. Sun , S. Tang , Y. Hou , et al., “Oxidized ATM‐mediated Glycolysis Enhancement in Breast Cancer‐associated Fibroblasts Contributes to Tumor Invasion through Lactate as Metabolic Coupling,” EBioMedicine 41 (2019): 370–383, 10.1016/j.ebiom.2019.02.025. 30799198 PMC6442874

[76] H. Xia , W. Wang , J. Crespo , et al., “Suppression of FIP200 and Autophagy by Tumor‐derived Lactate Promotes Naïve T Cell Apoptosis and Affects Tumor Immunity,” Science Immunology 2, no. 17 (2017): aan4631, 10.1126/sciimmunol.aan4631. PMC5774333 29150439

[77] A. Goenka , F. Khan , B. Verma , et al., “Tumor Microenvironment Signaling and Therapeutics in Cancer Progression,” Cancer Communications 43, no. 5 (2023): 525–561, 10.1002/cac2.12416. 37005490 PMC10174093

[78] B. Li , W. C. Lee , C. Song , L. Ye , E. D. Abel , and F. Long , “Both Aerobic Glycolysis and Mitochondrial Respiration Are Required for Osteoclast Differentiation,” The FASEB Journal 34, no. 8 (2020): 11058–11067, 10.1096/fj.202000771R. 32627870

[79] L. Wu , Y. Jin , X. Zhao , et al., “Tumor Aerobic Glycolysis Confers Immune Evasion through Modulating Sensitivity to T Cell‐mediated Bystander Killing via TNF‐α,” Cell Metabolism 35, no. 9 (2023): 1580–1596, 10.1016/j.cmet.2023.07.001. 37506695

[80] P. Clézardin , R. Coleman , M. Puppo , et al., “Bone Metastasis: Mechanisms, Therapies, and Biomarkers,” Physiological Reviews 101, no. 3 (2021): 797–855, 10.1152/physrev.00012.2019. 33356915

[81] C. Van Poznak , M. R. Somerfield , W. E. Barlow , et al., “Role of Bone‐Modifying Agents in Metastatic Breast Cancer: an American Society of Clinical Oncology–Cancer Care Ontario Focused Guideline Update,” Journal of Clinical Oncology 35, no. 35 (2017): 3978, 10.1200/JCO.2017.75.4614. 29035643

[82] Y. J. Cha , W. H. Jung , and J. S. Koo , “Differential Site‐Based Expression of Pentose Phosphate Pathway‐Related Proteins among Breast Cancer Metastases,” Disease Markers 2017 (2017): 7062517. 28260828 10.1155/2017/7062517 PMC5312075

[83] M. Wang , C. Yuan , Z. Wu , et al., “Paris Saponin VII Reverses Resistance to PARP Inhibitors by Regulating Ovarian Cancer Tumor Angiogenesis and Glycolysis through the RORα/ECM1/VEGFR2 Signaling Axis,” International Journal of Biological Sciences 20, no. 7 (2024): 2454–2475, 10.7150/ijbs.91861. 38725854 PMC11077377

[84] H. Wan , Q. Wang , X. Chen , et al., “WDR45 contributes to Neurodegeneration through Regulation of ER Homeostasis and Neuronal Death,” Autophagy 16, no. 3 (2020): 531–547, 10.1080/15548627.2019.1630224. 31204559 PMC6999610

