# Supplementary material for: SLC2A3‐Mediated Lactate Metabolism Promotes Lung Cancer Bone Metastasis by Modulating P53 Lactylation and Immune Evasion
Source: Adv Sci (Weinh). 2026 Feb 4;13(22):e16622. doi: 10.1002/advs.202516622 (PMC13088277; doi:10.1002/advs.202516622)
Supplement: Supplementary file 1 — Supporting File 1: advs74072‐sup‐0001‐SuppMat.pdf. [file ADVS-13-e16622-s002.pdf]

Supplemental Data for

SLC2A3-mediated Lactate Metabolism Promotes Lung Cancer Bone Metastasis by Modulating P53 Lactylation and Immune Evasion

Yi Ding[1,2,3,7], Yuying Tian[3,7], Wenjie Ren[3,7], Xianglin Hu[5,7], Mengjuan Li[3], Bei Liu[3], Chen chen[6], Yunhan Lu[3], Lei Li[3,4], Wangjun Yan[5], Kun Li[1,2,3,4]*

[1] Tongde Hospital of Zhejiang Province Affiliated to Zhejiang Chinese Medical University (Tongde Hospital of Zhejiang Province); Hangzhou, 310012, China; [2] Jiangong Hospital Clinical Research Center, East China Normal University; Shanghai, 200241, China; [3] Shanghai Key Laboratory of Regulatory Biology, School of Life Sciences, East China Normal University; Shanghai, 200241, China; [4] Chongqing Key Laboratory of Precision Optics, Chongqing Institute of East China Normal University, Chongqing, 401120, China; [5] Department of Musculoskeletal Oncology, Fudan University Shanghai Cancer Center, Shanghai 200032, China; [6] Ningxia Normal University, Guyuan, 756000, China; [7] These authors contributed equally to this work.

***Corresponding authors. Email:** kli@hsc.ecnu.edu.cn (K.L.).

Running title: SLC2A3 is a therapeutic target for lung cancer bone metastasis

Key words: Lung cancer bone metastasis, SLC2A3, Glycolysis, Lactylation, PD-1

Conflicts of interest: The authors have declared that no conflict of interest exists.

This supporting information contains

1. **Supplementary Figure 1.** SLC2A3 promotes bone metastasis of lung cancer. Related to Figure 1.
2. **Supplementary Figure 2.** A high-glucose environment promotes bone metastasis of lung cancer. Related to Figure 2.
3. **Supplementary Figure 3.** SLC2A3-mediated intracellular LA accumulation promotes P53 lactylation at K120. Related to Figure 3.
4. **Supplementary Figure 4.** SLC2A3 regulates LA accumulation to promote osteoclast differentiation. Related to Figure 4.
5. **Supplementary Figure 5.** SLC2A3 deficiency-induced LA depletion enhances PD-1 expression in $CD8^+$ T cells through reducing lactylation of P53 at K120. Related to Figure 5.
6. **Supplementary Figure 6.** Paris saponin Ⅶ as a potent SLC2A3 inhibitor suppresses lung cancer bone metastasis. Related to Figure 6.
7. **Supplementary Figure 7.** Paris saponin Ⅶ inhibits lung cancer bone metastasis by reducing lactate secretion. Related to Figure 7.
8. **Supplementary Figure 8.** Paris saponin Ⅶ enhances the sensitivity of lung cancer bone metastasis to anti-PD-1 treatment. Related to Figure 8.

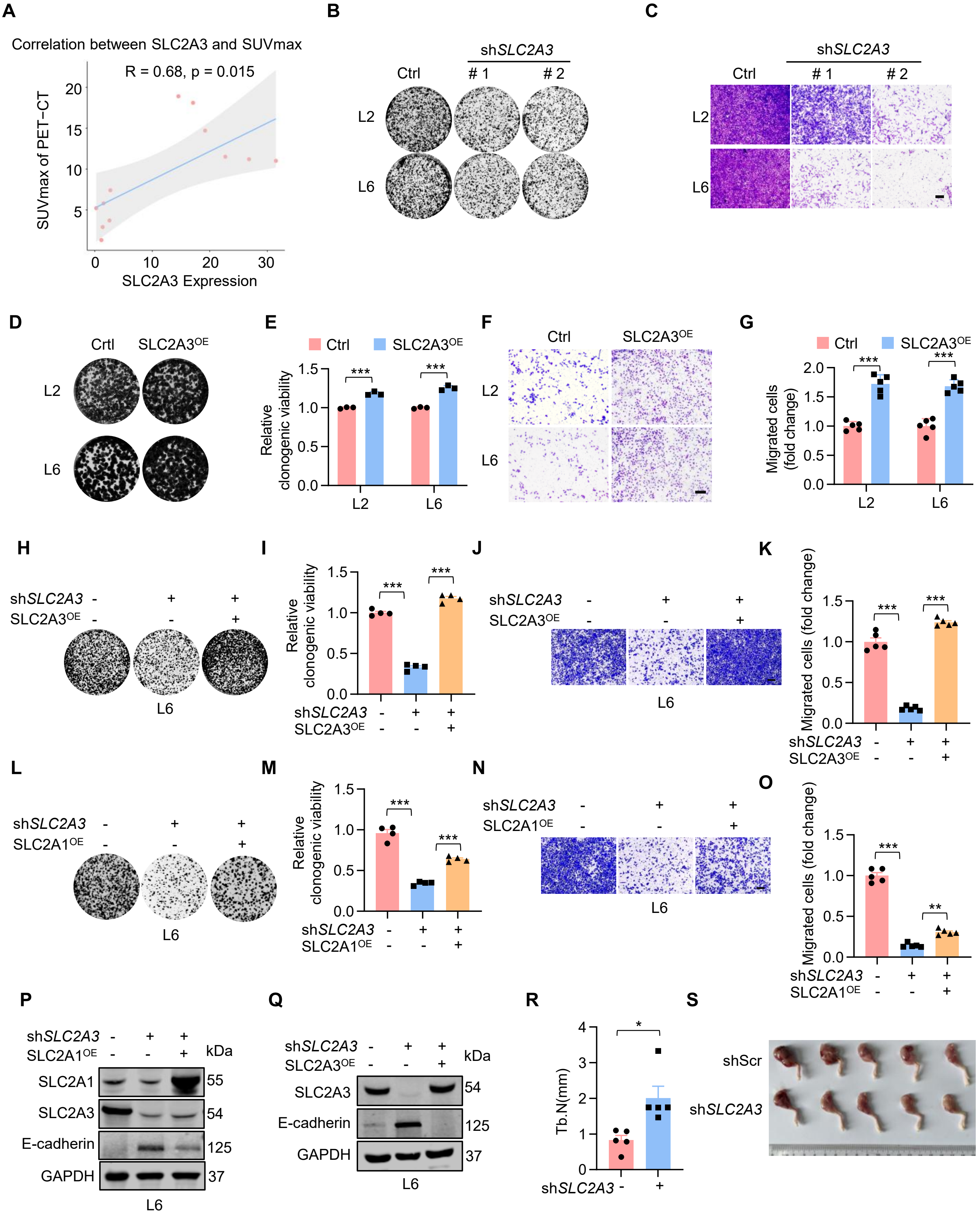

A
Correlation between SLC2A3 and SUVmax
R = 0.68, p = 0.015
SUVmax of PET−CT
SLC2A3 Expression
B
shSLC2A3
Ctrl
1
2
L2
L6
C
shSLC2A3
Ctrl
1
2
L2
L6
D
Crtl
SLC2A3OE
L2
L6
E
Ctrl
SLC2A3OE
Relative clonogenic viability
***
L2
L6
F
Ctrl
SLC2A3OE
L2
L6
G
Ctrl
SLC2A3OE
Migrated cells (fold change)
***
L2
L6
H
shSLC2A3
SLC2A3OE
L6
I
Relative clonogenic viability
shSLC2A3
SLC2A3OE
J
shSLC2A3
SLC2A3OE
L6
K
Migrated cells (fold change)
shSLC2A3
SLC2A3OE
L
shSLC2A3
SLC2A1OE
L6
M
Relative clonogenic viability
shSLC2A3
SLC2A1OE
N
shSLC2A3
SLC2A1OE
L6
O
Migrated cells (fold change)
**
shSLC2A3
SLC2A1OE
P
shSLC2A3
SLC2A1OE
kDa
SLC2A1
SLC2A3
E-cadherin
GAPDH
55
54
125
37
L6
Q
shSLC2A3
SLC2A3OE
kDa
SLC2A3
E-cadherin
GAPDH
54
125
37
L6
R
Tb.N(mm)
*
shSLC2A3
S
shScr
shSLC2A3

Supplementary Figure 1. SLC2A3 promotes bone metastasis of lung cancer. Related to Figure 1.

A. Pearson Correlation Analysis of SUV_{max} and SLC2A3 expression. **B.** Colony formation of metastatic cells after knocking down SLC2A3 expression. Images were taken 14 days after seeding. **C.** Representative images of transwell assay show cell migration after knocking down SLC2A3 expression. Images were taken 24 hours after seeding. Scale bar: 400 μm. **D.** Colony formation of metastatic cells after overexpression of SLC2A3. Images were taken 14 days after seeding. **E.** Statistical analysis of the colony formation results. Each value represents mean ± SEM (n = 3). ***P < 0.001; P values were measured by unpaired two-tailed Student's t test. **F.** Representative images of transwell assay show cell migration after overexpression of SLC2A3. Images were taken 24 hours after seeding. Scale bar: 400 μm. **G.** Statistical analysis of the number of migrated cells in (G). Each value represents mean ± SEM (n = 5). ***P < 0.001; P values were measured by unpaired two-tailed Student's t test. **H.** Colony formation of L6 cells following sequential SLC2A3 knockdown and overexpression. Images were taken 14 days after seeding. **I.** Statistical analysis of the colony formation results. Each value represents mean ± SEM (n = 4). ***P < 0.001; P values were measured by one-way ANOVA with Tukey's multiple comparison test. **J.** Representative images of transwell assay show cell migration of L6 cells following sequential SLC2A3 knockdown and overexpression. Images were taken 24 hours after seeding. Scale bar: 100 μm. **K.** Statistical analysis of the number of migrated cells in (J). Each value represents mean ± SEM (n = 5). ***P < 0.001; P values were measured by one-way ANOVA with Tukey's multiple comparison test. **L.** Colony formation of L6 cells after sequential SLC2A3 knockdown and SLC2A1 overexpression. Images were taken 14 days after seeding. **M.** Statistical analysis of the colony formation results. Each value represents mean ± SEM (n = 4). ***P < 0.001; P values were measured by one-way ANOVA with Tukey's multiple comparison test. **N.** Representative images of transwell assay show cell migration of L6 cells after sequential SLC2A3 knockdown and SLC2A1 overexpression. Images were taken 24 hours after seeding. Scale bar: 100 μm. **O.** Statistical analysis of the number of migrated cells in (N). Each value represents mean ± SEM (n = 5). **P < 0.01, ***P < 0.001; P values were measured by one-way ANOVA with Tukey's multiple comparison test. **P.** Western blot analysis shows the expression of E-cadherin in L6 cells after sequential SLC2A3 knockdown and SLC2A1 overexpression. **Q.** Western blot analysis of E-cadherin expression in L6 cells following sequential SLC2A3 knockdown and overexpression. **R.** Bar graph shows the quantitative micro-CT analysis of trabecular bone from the (Figure 1O) tibia. Tb. N, trabecular number (per mm). Error bars are mean ± SEM. *P < 0.05 using unpaired two-tailed Student's t-test. **S.** Tumor volume in the (Figure 1R) intratibial injection model (n = 5).

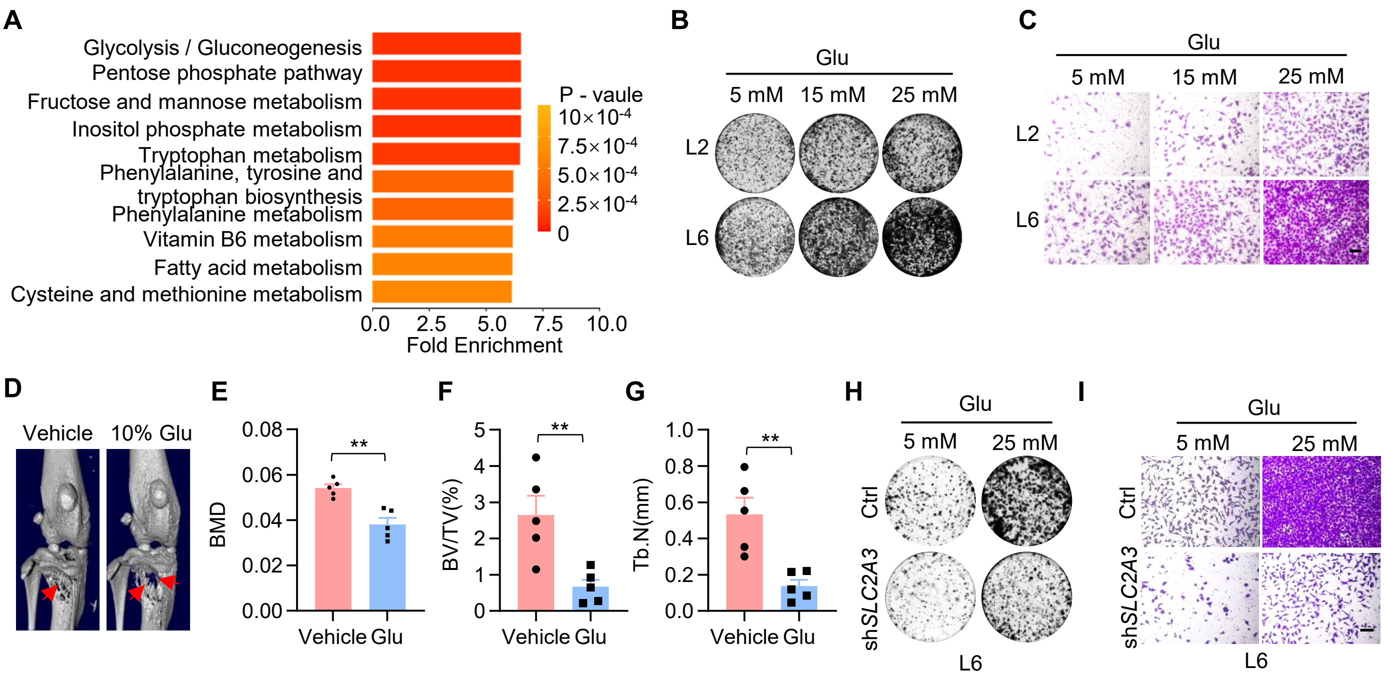

Supplementary Figure 2. A high-glucose environment promotes bone metastasis of lung cancer. Related to Figure2.

A. Metabolic pathway enrichment analysis reveals the top 10 significantly differential pathways in L6 cells after SLC2A3 knockdown. **B.** Colony formation of metastatic cells after treatment with glucose at the concentration of 5mM (Low), 15mM (Medium) and 25mM (High). Images were taken 14 days after seeding. **C.** Representative images of transwell assay show cell migration after treatment with glucose at the concentration of 5mM (Low), 15mM (Medium) and 25mM (High). Images were taken 24 hours after seeding. Scale bar: 100 μm. **D.** Representative micro-CT images of the tibia from the mice in (Figure 2G). **E-G.** Bar graph shows the quantitative micro-CT analysis of trabecular bone in (D). BMD, bone mineral density; BV/TV, bone volume total/volume; Tb. N, trabecular number (per mm). Error bars are mean ± SEM. $^{**}P < 0.01$ using unpaired two-tailed Student's t-test. **H.** Colony formation of L6 cells after knocking down SLC2A3 and treatment with glucose at the concentration of 5mM (Low) and 25mM (High). Images were taken 14 days after seeding. **I.** Representative images of transwell assay show cell migration of L6 cells after knocking down SLC2A3 and treatment with glucose at the concentration of 5mM (Low) and 25mM (High). Images were taken 24 hours after seeding. Scale bar: 100 μm.

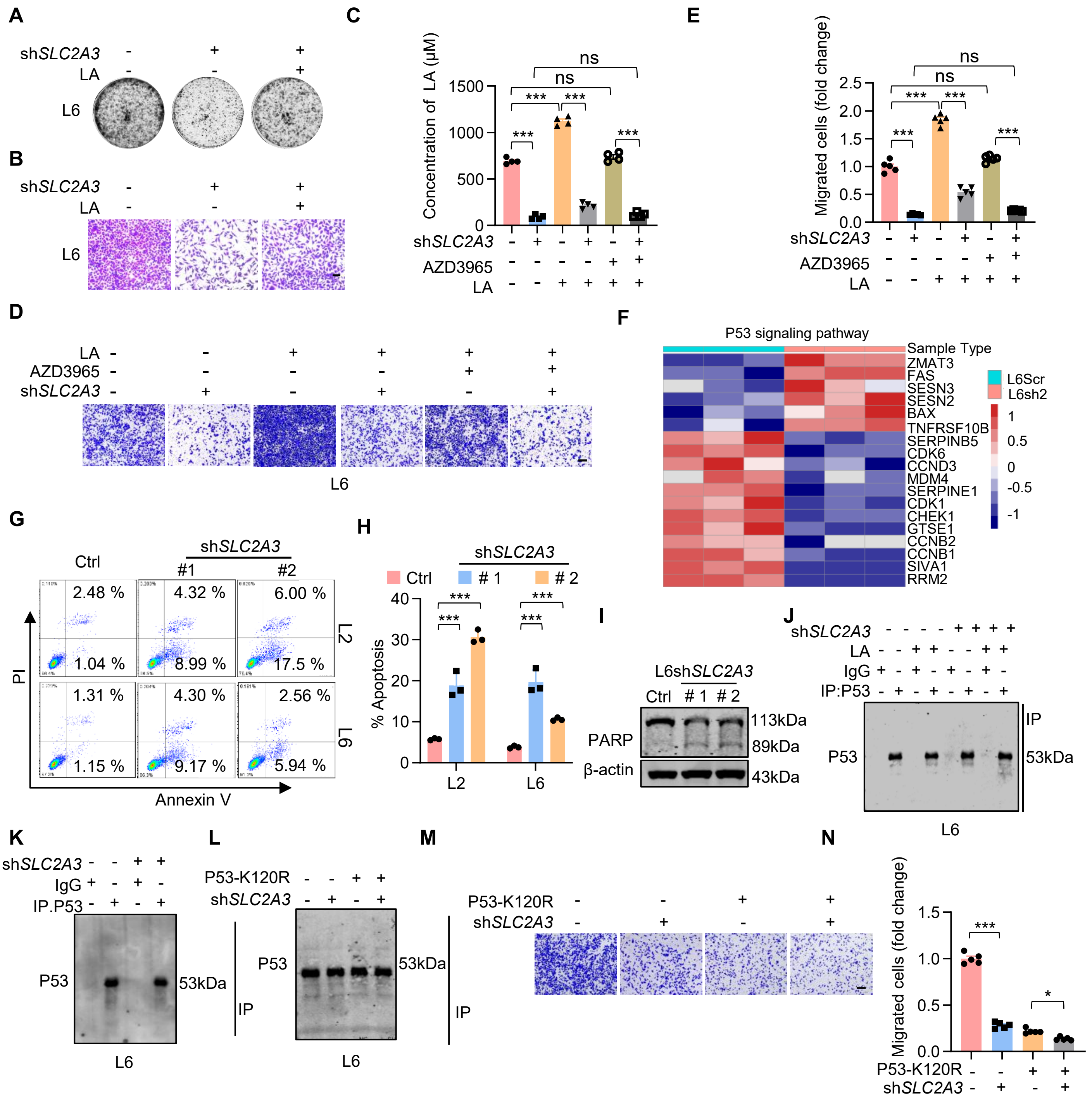

A
shSLC2A3 - + +
LA - - +
L6
B
shSLC2A3 - + +
LA - - +
L6
C
Concentration of LA (μM)
1500
1000
500
0
ns
ns
***
***
***
***
shSLC2A3
AZD3965
LA
E
Migrated cells (fold change)
2.5
2.0
1.5
1.0
0.5
0.0
ns
ns
***
***
***
***
shSLC2A3
AZD3965
LA
D
LA - - + + + +
AZD3965 - - - - + +
shSLC2A3 - + - + - +
L6
F
P53 signaling pathway
Sample Type
ZMAT3
FAS
SESN3
SESN2
BAX
TNFRSF10B
SERPINB5
CDK6
CCND3
MDM4
SERPINE1
CDK1
CHEK1
GTSE1
CCNB2
CCNB1
SIVA1
RRM2
L6Scr
L6sh2
1
0.5
0
-0.5
-1
G
Ctrl
shSLC2A3
#1
#2
2.48 %
4.32 %
6.00 %
1.04 %
8.99 %
17.5 %
1.31 %
4.30 %
2.56 %
1.15 %
9.17 %
5.94 %
L2
L6
PI
Annexin V
H
shSLC2A3
Ctrl
1
2
% Apoptosis
40
30
20
10
0
***
***
***
***
L2
L6
I
L6shSLC2A3
Ctrl
1
2
PARP
β-actin
113kDa
89kDa
43kDa
J
shSLC2A3 - - - - + + + +
LA - - + + - - + +
IgG + - + - + - + -
IP:P53 - + - + - + - +
P53
53kDa
IP
L6
K
shSLC2A3 - - + +
IgG + - + -
IP.P53 - + - +
P53
53kDa
L6
L
P53-K120R - - + +
shSLC2A3 - + - +
P53
53kDa
IP
IP
L6
M
P53-K120R - - + +
shSLC2A3 - + - +
N
Migrated cells (fold change)
1.5
1.0
0.5
0.0
***
*
P53-K120R - - + +
shSLC2A3 - + - +

Supplementary Figure 3. SLC2A3-mediated intracellular LA accumulation promotes P53 lactylation at K120. Related to Figure 3.

A. Colony formation of L6 cells after knocking down SLC2A3 and treatment with or without LA (20 mM). Images were taken 14 days after seeding. **B.** Representative images of transwell assay show cell migration of L6 cells after knocking down SLC2A3 and treatment with or without LA (20 mM). Images were taken 24 hours after seeding. Scale bar: 100 μm. **C.** The bar graph shows the intracellular LA levels in L6 cells following SLC2A3 knockdown with treatment of LA (20 mM) and AZD3965 (2 nM). AZD3965, an MCT1 inhibitor. Each value represents mean ± SEM (n = 4). ***$P < 0.001$; P values were measured by one-way ANOVA with Tukey's multiple comparison test. **D.** Representative images of transwell assay show cell migration of L6 cells after knocking down SLC2A3 and treatment with LA (20 mM) and AZD3965 (2 nM). Images were taken 24 hours after seeding. Scale bar: 100 μm. **E.** Statistical analysis of the number of migrated cells in (D). Each value represents mean ± SEM (n = 5). ***$P < 0.001$; P values were measured by one-way ANOVA with Tukey's multiple comparison test. **F.** Heatmap of significantly differentially expressed proteins involved in the p53 signaling pathway in L6shScr versus L6sh*SLC2A3#2* cells (n = 3). **G.** Flow cytometry analysis of apoptosis in L2 and L6 cells after SLC2A3 knockdown. **H.** Statistical analysis of the apoptosis in (G). Each value represents mean ± SEM (n = 3), ***$P < 0.001$; P values were measured by one-way ANOVA with Tukey's multiple comparison test. **I.** Western blot analysis of PARP1 expression after knocking down SLC2A3. **J.** L6shScr and L6shSLC2A3 cells treated with or without LA (20 mM) were lysed with CHAPS lysis buffer. Immunoprecipitations of cell lysates with anti-p53 or anti-IgG, followed by immunoblotting with antibodies against the indicated proteins. **K.** L6shScr and L6shSLC2A3 cells were lysed with CHAPS lysis buffer. Immunoprecipitations of cell lysates with anti-p53 or anti-IgG, followed by immunoblotting with antibodies against the indicated proteins. **L.** L6 cells after SLC2A3 knockdown and/or p53-K120 mutant were lysed with CHAPS lysis buffer. Immunoprecipitations of cell lysates with anti-HA agarose, followed by immunoblotting with antibodies against the indicated proteins. **M.** Representative images of transwell assay show cell migration of L6 cells after sequential knockdown of SLC2A3 and p53-K120 mutation introduction. Images were taken 24 hours after seeding. Scale bar: 100 μm. **N.** Statistical analysis of the number of migrated cells in (M). Each value represents mean ± SEM (n = 5). *$P < 0.05$, ***$P < 0.001$; P values were measured by one-way ANOVA with Tukey's multiple comparison test.

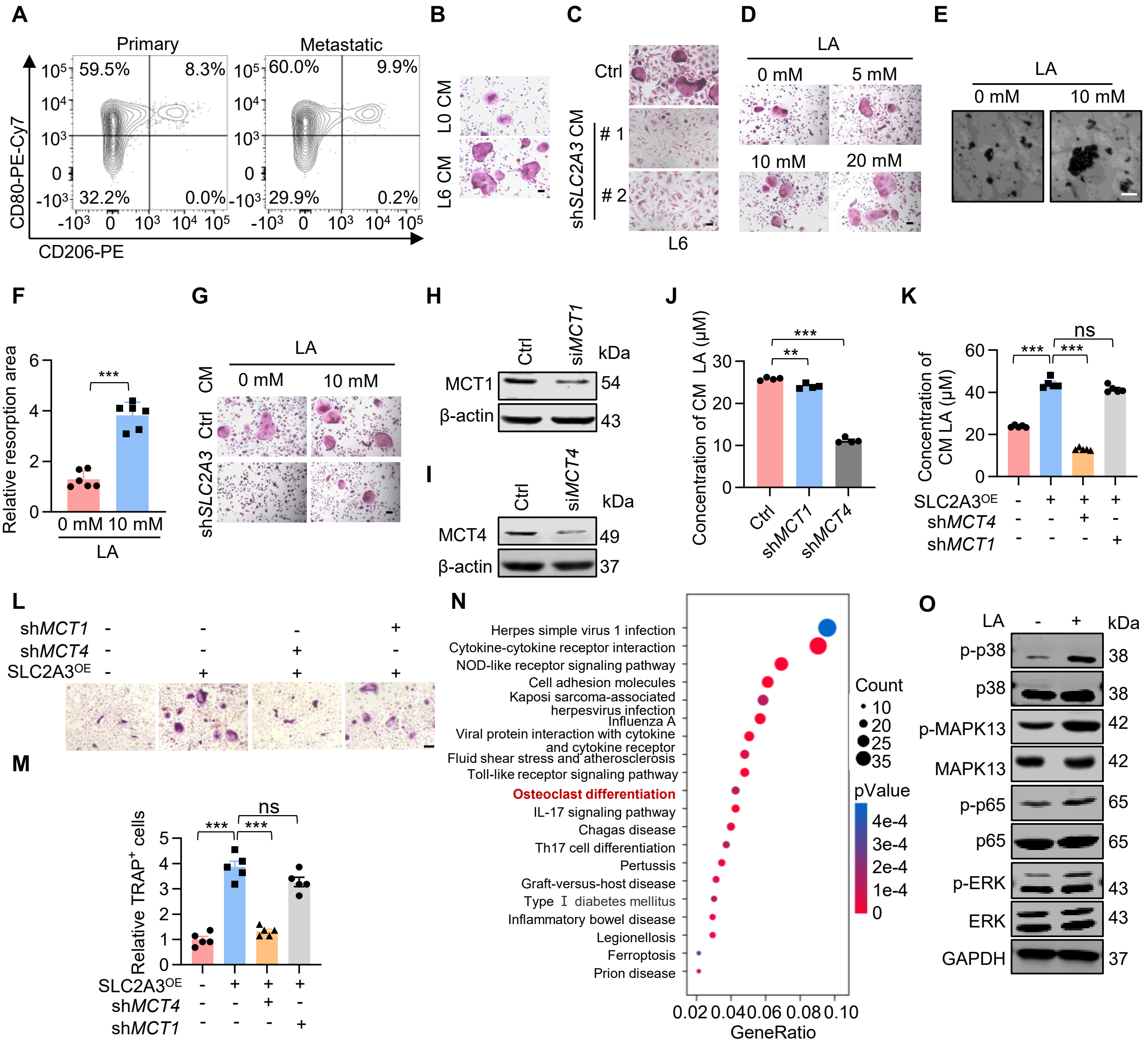

A
Primary
Metastatic
59.5%
8.3%
32.2%
0.0%
60.0%
9.9%
29.9%
0.2%
CD80-PE-Cy7
CD206-PE
B
L0 CM
L6 CM
C
Ctrl
shSLC2A3 CM
1
2
L6
D
LA
0 mM
5 mM
10 mM
20 mM
E
LA
0 mM
10 mM
F
Relative resorption area
0 mM 10 mM
LA
G
LA
0 mM
10 mM
CM
Ctrl
shSLC2A3
H
Ctrl
siMCT1
kDa
MCT1
β-actin
54
43
I
Ctrl
siMCT4
kDa
MCT4
β-actin
49
37
J
Concentration of CM LA (μM)
Ctrl
shMCT1
shMCT4
K
Concentration of CM LA (μM)
SLC2A3OE
shMCT4
shMCT1
L
shMCT1
shMCT4
SLC2A3OE
M
Relative TRAP+ cells
SLC2A3OE
shMCT4
shMCT1
N
Herpes simplex virus 1 infection
Cytokine-cytokine receptor interaction
NOD-like receptor signaling pathway
Cell adhesion molecules
Kaposi sarcoma-associated herpesvirus infection
Influenza A
Viral protein interaction with cytokine and cytokine receptor
Fluid shear stress and atherosclerosis
Toll-like receptor signaling pathway
Osteoclast differentiation
IL-17 signaling pathway
Chagas disease
Th17 cell differentiation
Pertussis
Graft-versus-host disease
Type Ⅰ diabetes mellitus
Inflammatory bowel disease
Legionellosis
Ferroptosis
Prion disease
GeneRatio
Count
pValue
O
LA
kDa
p-p38
p38
p-MAPK13
MAPK13
p-p65
p65
p-ERK
ERK
GAPDH

Supplementary Figure 4. SLC2A3 regulates LA accumulation to promote osteoclast differentiation. Related to Figure 4.

A. Representative FACS plots of M1 and M2 macrophages infiltration in CMT167 tumors from mice following orthotopic lung injection (Primary) and intratibial injection (Metastatic). **B.** Representative TRAP staining images of osteoclast differentiation from BMMs treated with L0 or L6 CM. Scale bars: 100 μm **C.** Representative TRAP staining images of osteoclast differentiation from BMMs treated with L6shScr or L6shSLC2A3 CM. Scale bars: 100 μm. **D.** Representative TRAP staining images of osteoclast differentiation from BMMs treated with LA at the concentration of 5mM, 10mM and 20mM. Scale bars: 100 μm. **E.** Representative images of bone erosion pits from osteoclasts treated with or without LA (10 mM). Scale bar: 100 μm. **F.** Quantification of the osteoclast resorption area in (E). n = 6 mice per group. P value was measured by unpaired two-tailed Student's t-test. **G.** Representative TRAP staining images of osteoclast differentiation from BMMs treated with L6shScr or L6shSLC2A3 CM and supplemented with or without LA (10 mM). Scale bars: 100 μm. **H.** Western blot analysis shows the knockdown efficiency of MCT1. **I.** Western blot analysis shows the knockdown efficiency of MCT4. **J.** Bar graph shows LA levels in CM of L0 cells after knocking down MCT1 or MCT4. Each value represents mean ± SEM (n = 4). **$P < 0.01$, ***$P < 0.001$, P values were measured by unpaired two-tailed Student's t test. **K.** Bar graph shows LA levels in CM of L0 cells following SLC2A3 overexpression and subsequent knockdown of MCT1 or MCT4. ***$P < 0.001$, P values were measured by one-way ANOVA with Tukey's multiple comparison test. **L.** Representative TRAP staining images of osteoclast differentiation from BMMs treated with L0, L0SLC2A3^{OE}, L0SLC2A3^{OE}sh*MCT1* and L0SLC2A3^{OE}sh*MCT4* CM. Scale bars: 100 μm. **M.** Statistical analysis of the number of Trap^{+} cells in (L). The relative osteoclast differentiation ability was normalized to vehicle-treated control. Each value represents mean ± SEM (n = 5). ***$P < 0.001$, P values were measured by one-way ANOVA with Tukey's multiple comparison test. **N.** Pathway enrichment analysis of the top 20 significantly differential pathways in osteoclast differentiation from LA-treated BMMs (10 mM). ($P < 0.05$, FC > 1.5 or < 0.67, n = 3). **O.** Western blot analysis shows the protein expression levels of the MAPK signaling pathway.

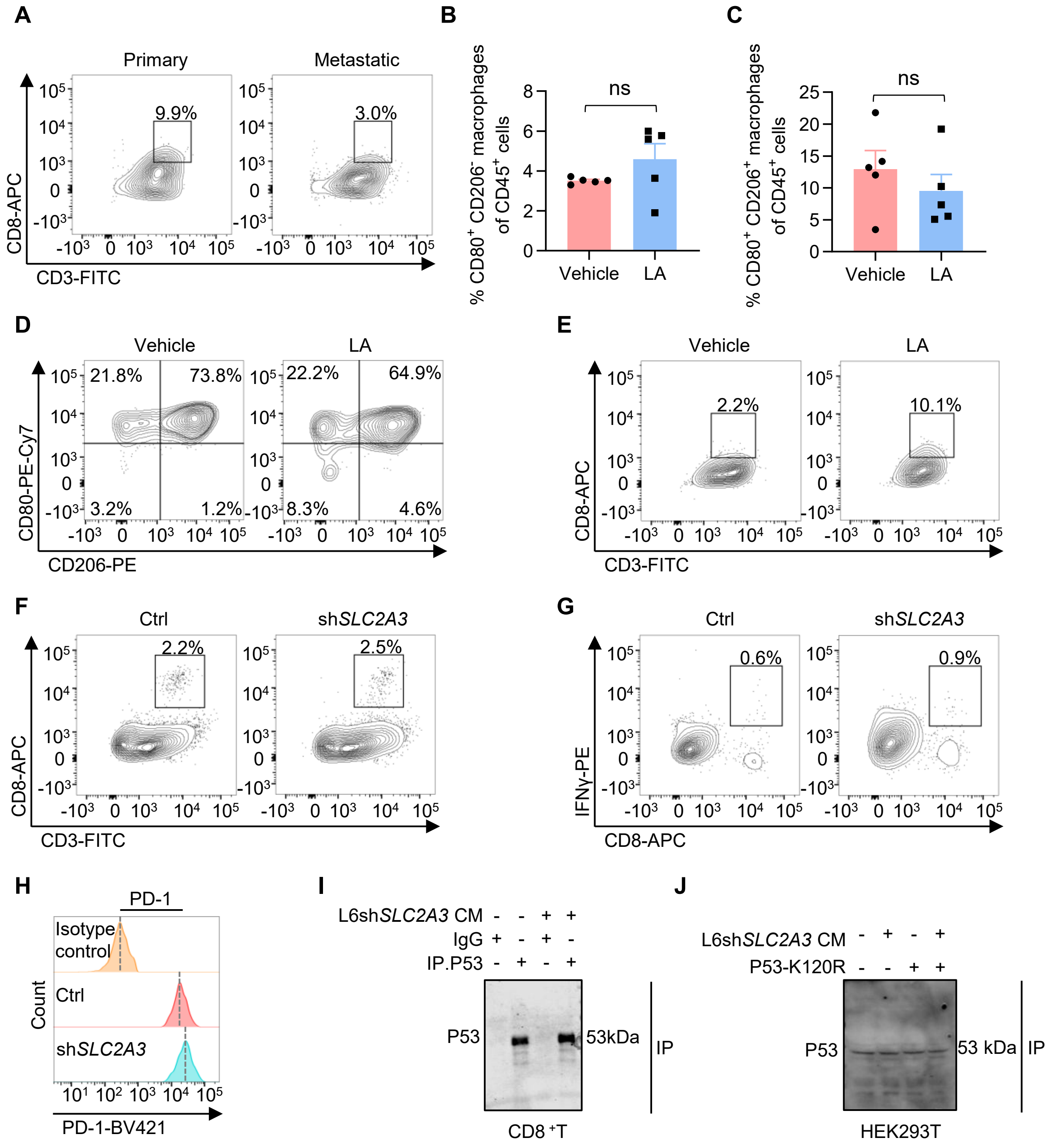
A
Primary
Metastatic
9.9%
3.0%
CD8-APC
CD3-FITC
B
% CD80+ CD206- macrophages of CD45+ cells
ns
Vehicle
LA
C
% CD80+ CD206+ macrophages of CD45+ cells
ns
Vehicle
LA
D
Vehicle
LA
21.8%
73.8%
3.2%
1.2%
22.2%
64.9%
8.3%
4.6%
CD80-PE-Cy7
CD206-PE
E
Vehicle
LA
2.2%
10.1%
CD8-APC
CD3-FITC
F
Ctrl
shSLC2A3
2.2%
2.5%
CD8-APC
CD3-FITC
G
Ctrl
shSLC2A3
0.6%
0.9%
IFNγ-PE
CD8-APC
H
PD-1
Isotype control
Ctrl
shSLC2A3
Count
PD-1-BV421
I
L6shSLC2A3 CM - - + +
IgG + - + -
IP.P53 - + - +
P53
53kDa
IP
CD8 +T
J
L6shSLC2A3 CM - + - +
P53-K120R - - + +
P53
53 kDa
IP
HEK293T

Supplementary Figure 5. SLC2A3 deficiency-induced LA depletion enhances PD-1 expression in $CD8^+$ T cells through reducing lactylation of P53 at K120. Related to Figure 5.

A. Representative FACS plots of $CD8^+$ T cells infiltration in CMT167 tumors from the mice following orthotopic lung injection (Primary) and intratibial injection (Metastatic). **B-C.** Quantitative FACS analysis of M1 and M2 macrophages infiltration in CMT167 tumors from the mice following intratibial injection, with or without LA treatment. n=5 mice per group. *P* value was measured by unpaired two-tailed Student's t-test. **D.** Representative FACS plots of M1 and M2 macrophages infiltration in CMT167 tumors from the mice following intratibial injection, with or without LA treatment. **E.** Representative FACS plots of $CD8^+$ T cells infiltration in CMT167 tumors from the mice following intratibial injection, with or without LA treatment. **F-G.** Representative FACS plots of $CD8^+$ in $CD45^+$ cells and $IFN\gamma^+$ in $CD8^+$ T cells within CMT167 wild-type and SLC2A3-knockdown tumors from the mice following intratibial injection. **H.** Representative histogram plots of PD-1 expression in $CD8^+$ T cells within CMT167 wild-type and SLC2A3-knockdown tumors from the mice following intratibial injection. **I.** $CD8^+$ T cells were isolated from C57BL/6 mice spleens and stimulated with L6shScr or L6shSLC2A3 CM for 24 hours. $CD8^+$ T cells were then lysed with CHAPS lysis buffer. Immunoprecipitations of cell lysates were performed using anti-p53 or anti-IgG, followed by immunoblotting with antibodies against the indicated proteins. **J.** HEK293T-p53-WT and HEK293T-p53-K120R cells after stimulating with L6shScr or L6shSLC2A3 CM were lysed with CHAPS lysis buffer. Immunoprecipitations of cell lysates with anti-HA agarose, followed by immunoblotting with antibodies against the indicated proteins.

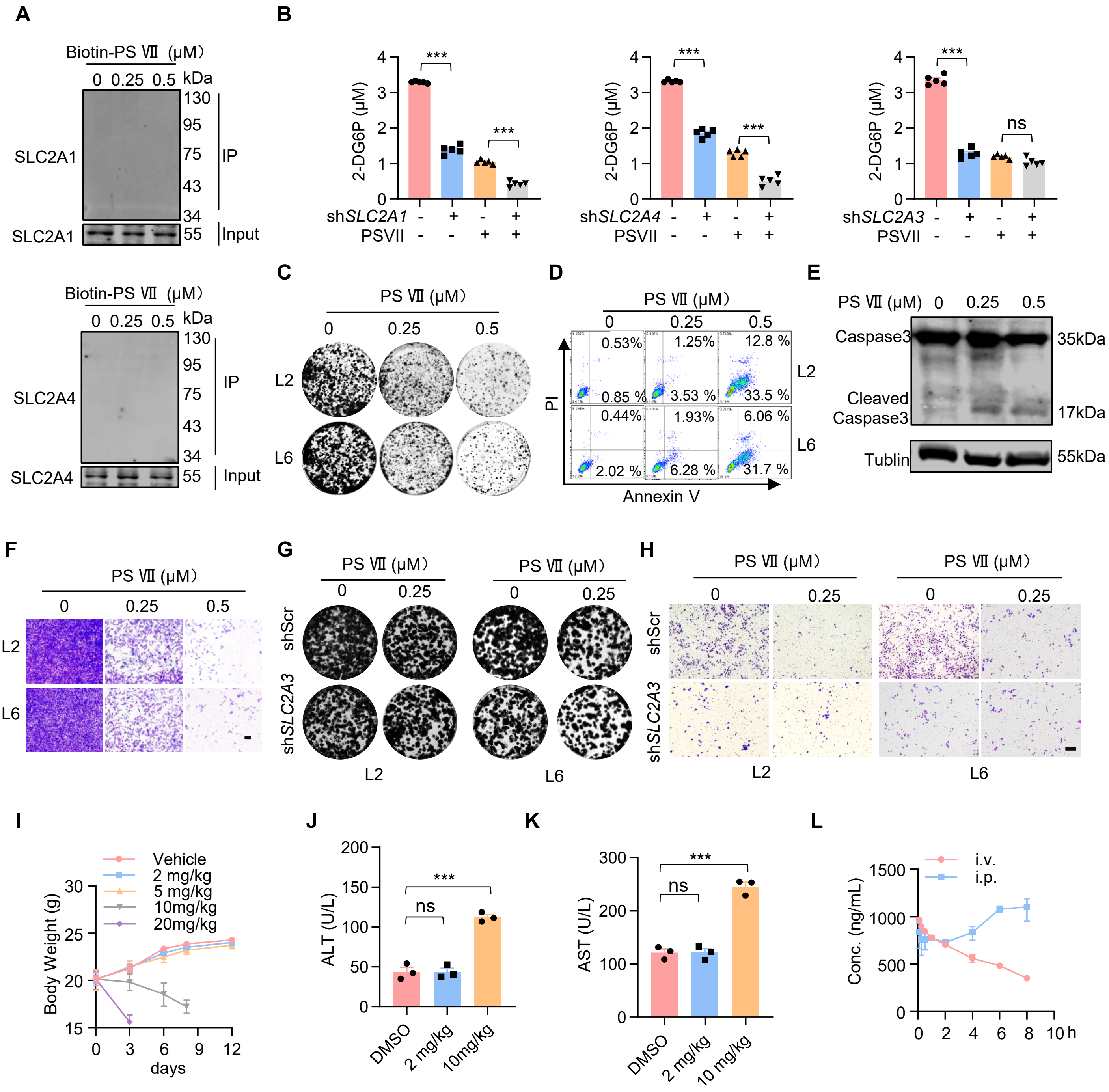
A
Biotin-PS Ⅶ (μM)
0 0.25 0.5 kDa
130
95
75
43
34
55
SLC2A1
IP
Input
SLC2A4
B
2-DG6P (μM)
***
ns
shSLC2A1
shSLC2A4
shSLC2A3
PSVII
C
PS Ⅶ (μM)
L2
L6
D
0.53%
1.25%
12.8 %
0.85 %
3.53 %
33.5 %
0.44%
1.93%
6.06 %
2.02 %
6.28 %
31.7 %
PI
Annexin V
E
Caspase3
Cleaved Caspase3
Tublin
35kDa
17kDa
55kDa
F
G
shScr
shSLC2A3
H
I
Vehicle
2 mg/kg
5 mg/kg
10mg/kg
20mg/kg
Body Weight (g)
days
J
ALT (U/L)
DMSO
10mg/kg
K
AST (U/L)
10 mg/kg
L
i.v.
i.p.
Conc. (ng/mL)
10 h

Supplementary Figure 6. Paris saponin Ⅶ as a potent SLC2A3 inhibitor suppresses lung cancer bone metastasis. Related to Figure 6.

A. L6 protein lysates were treated with biotin or biotin-Paris saponin Ⅶ (biotin-PS Ⅶ) for 3 hours, performed pull-down using Streptavidin Agarose Resin, followed by western blot. **B.** Glucose uptake capacity assay in L6sh*SLC2A1*, L6sh*SLC2A4* and L6sh*SLC2A3* cells after treatment with Paris saponin Ⅶ (0.5 μM). Each value represents mean ± SEM (n = 5). ***P < 0.001; P values were measured by one-way ANOVA with Tukey's multiple comparison test. **C.** Colony formation of metastatic lung cancer cells after treatment with Paris saponin Ⅶ at the concentration of 0.25 μM and 0.5 μM. Images were taken 14 days after seeding. **D.** Flow cytometry analysis of apoptosis in L2 and L6 cells after treatment with Paris saponin Ⅶ at the concentration of 0.25 μM and 0.5 μM. **E.** Western blot analysis of caspase3 expression after treatment with Paris saponin Ⅶ at the concentration of 0.25 μM and 0.5 μM. **F.** Representative images of transwell assay show cell migration after treatment with Paris saponin Ⅶ at the concentration of 0.25 μM and 0.5 μM. Images were taken 24 hours after seeding. Scale bar: 400 μm. **G.** Colony formation of metastatic lung cancer cells after knocking down SLC2A3 and treatment with Paris saponin Ⅶ (0.25 μM). Images were taken 14 days after seeding. **H.** Representative images of transwell assay show cell migration of metastatic lung cancer cells after knocking down SLC2A3 and treatment with Paris saponin Ⅶ (0.25 μM). Images were taken 24 hours after seeding. Scale bar: 400 μm. **I.** The body weight of the ICR mice (n = 5). **J, K.** Effects of Paris saponin Ⅶ on clinical chemistry analysis in mice. Serum samples were collected from 6-week-old ICR mice (n = 3). Liver function indexes include ALT and AST. The value is shown as mean ± SEM. ***P < 0.001. **L.** The logarithmic semi-logarithmic curve of blood drug concentration-time for Paris saponin Ⅶ.

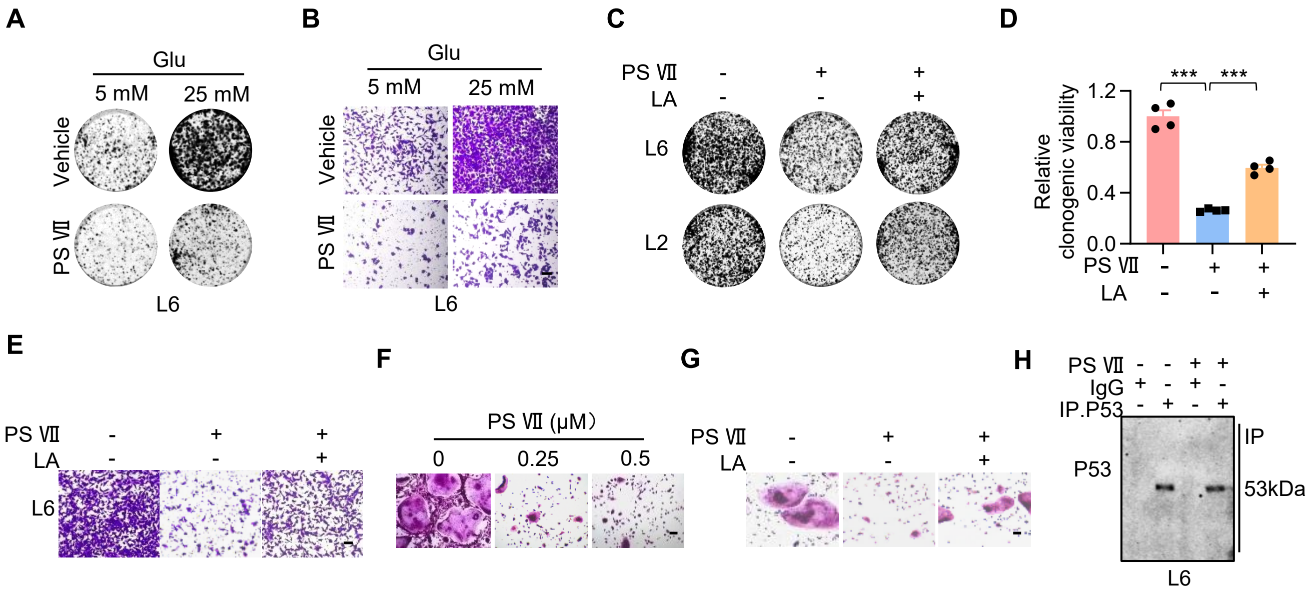

Supplementary Figure 7. Paris saponin Ⅶ inhibits lung cancer bone metastasis by reducing lactate secretion. Related to Figure 7.

A. Colony formation of L6 cells after glucose (5 mM or 25 mM) and Paris saponin Ⅶ (0.25 μM) treatment. Images were taken 14 days after seeding. **B.** Representative images of transwell assay show cell migration in L6 cells after glucose (5 mM or 25 mM) and Paris saponin Ⅶ (0.25 μM) treatment. Images were taken 24 hours after seeding. Scale bar: 100 μm. **C.** Colony formation of metastatic lung cancer cells after LA (20 mM) and Paris saponin Ⅶ (0.25 μM) treatment. Images were taken 14 days after seeding. **D.** Statistical analysis of the colony formation results in (C). Each value represents mean ± SEM (n = 4). ***P < 0.001; P values were measured one-way ANOVA with Tukey's multiple comparison test. **E.** Representative images of transwell assay show cell migration in L6 cells after LA (20 mM) and Paris saponin Ⅶ (0.25 μM) treatment. Images were taken 24 hours after seeding. Scale bar: 100 μm. **F.** Representative images of TRAP staining showing osteoclast differentiation from BMMs. BMMs were stimulated with conditioned medium collected from L6 cells pretreated with Paris saponin Ⅶ at concentrations of 0.25 μM and 0.5 μM. Scale bars: 100 μm. **G.** Representative TRAP staining images of osteoclast differentiation from BMMs. BMMs were treated directly with 20 mM LA, or stimulated with conditioned medium from L6 cells pretreated with Paris saponin Ⅶ at 0.25 μM and 0.5 μM. Scale bars: 100 μm. **H.** L6 cells were treated with Paris saponin Ⅶ (0.25 μM) for 12 h, and WCEs were collected for IP with anti-p53 antibody, followed by immunoblotting.

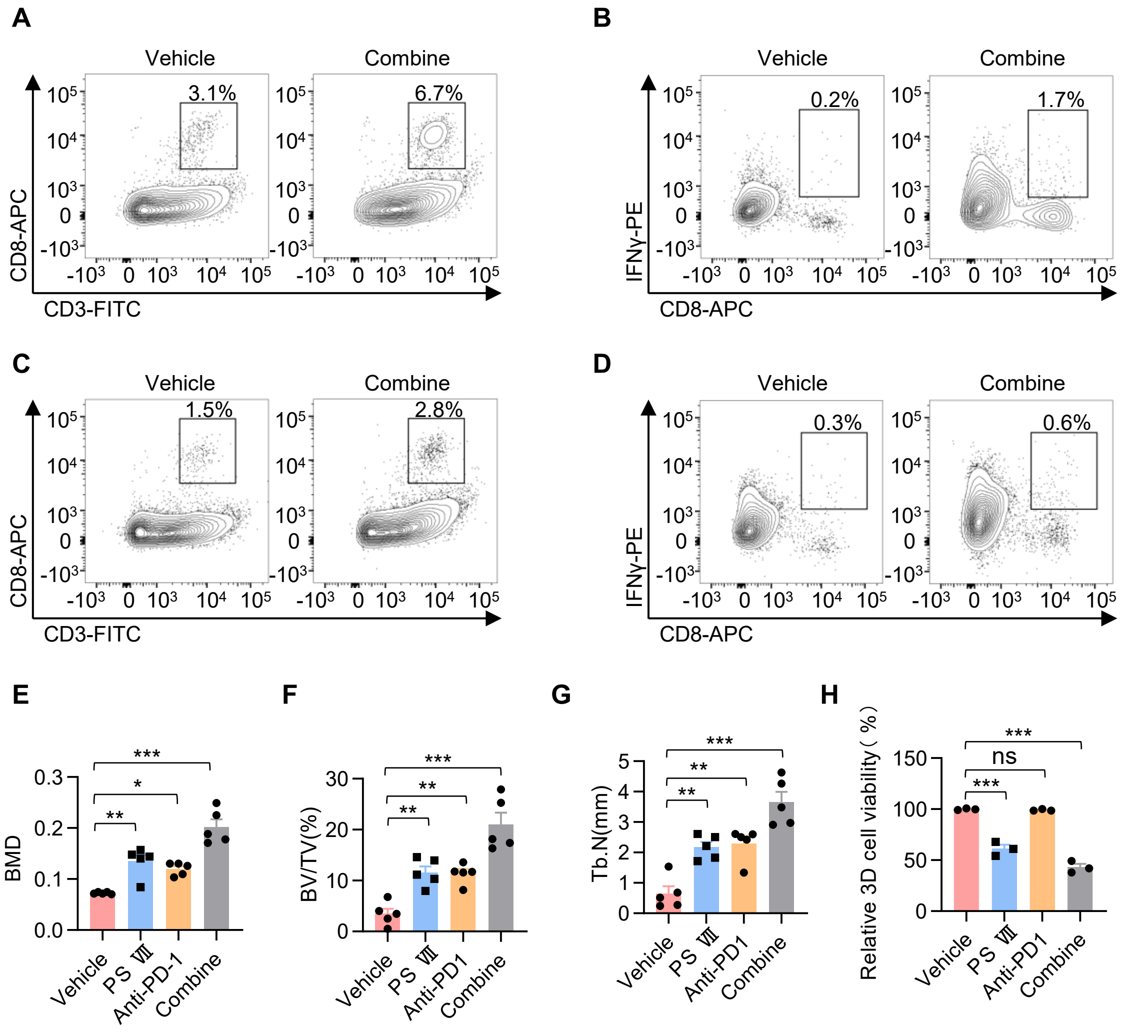

Supplementary Figure 8. Paris saponin Ⅶ enhances the sensitivity of lung cancer bone metastasis to anti-PD-1 treatment. Related to Figure 8.

A-B. Representative FACS plots of $CD8^+$ in $CD45^+$ cells and $IFN\gamma^+$ in $CD8^+$ T cells within CMT167 tumors from the mice following intratibial injection, with or without SLC2A3 genetic blockade and anti-PD-1 treatment. **C-D.** Representative FACS plots of $CD8^+$ in $CD45^+$ cells and $IFN\gamma^+$ in $CD8^+$ T cells within CMT167 tumors from the mice following intratibial injection, with or without Paris saponin Ⅶ and anti-PD-1 treatment. **E-G.** Bar graph shows the quantitative micro-CT analysis of trabecular bone in (Figure 8I). BMD, bone mineral density; BV/TV, bone volume total/volume; Tb. N, trabecular number (per mm). Error bars are mean ± SEM. **$P < 0.01$, ***$P < 0.001$ using one-way ANOVA with Tukey's multiple comparison test. **H.** Relative quantification of 3D cell viability from (Figure 8N). Error bars are mean ± SEM. ***$P < 0.001$; P values were calculated using one-way ANOVA with Tukey's multiple comparison test.
