# Supplementary material for: SLC2A3‐Mediated Lactate Metabolism Promotes Lung Cancer Bone Metastasis by Modulating P53 Lactylation and Immune Evasion
Source: Adv Sci (Weinh). 2026 Feb 4;13(22):e16622. doi: 10.1002/advs.202516622 (PMC13088277; doi:10.1002/advs.202516622)

Original blot images

Full unedited gel for figure 1C

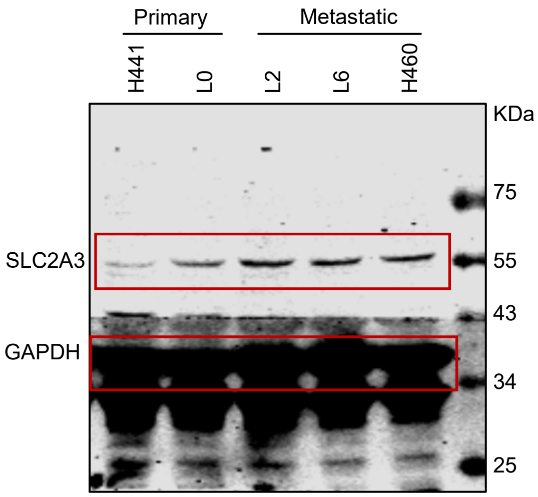

Full unedited gels for figure 1K

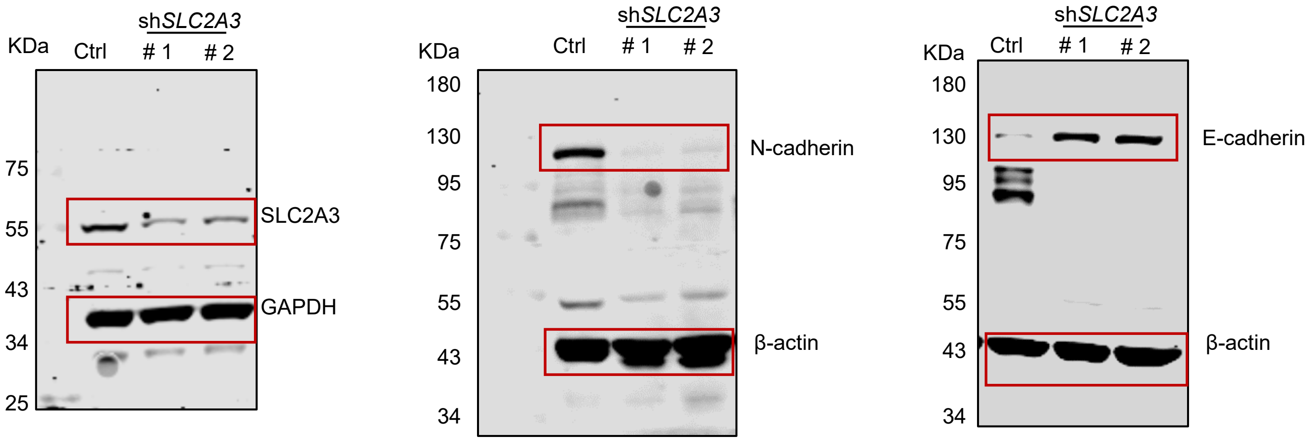

Full unedited gel for figure 2N

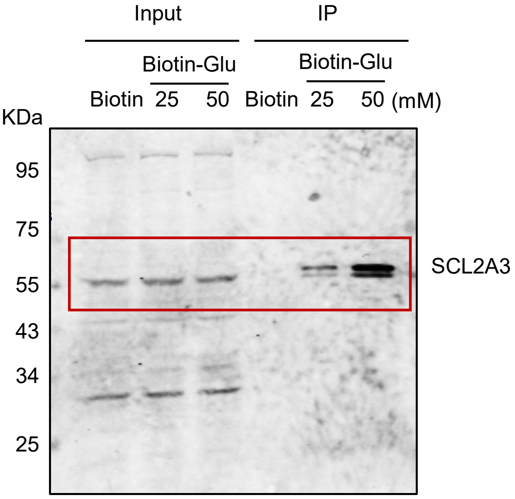

Original blot images

Full unedited gels for figure 3B

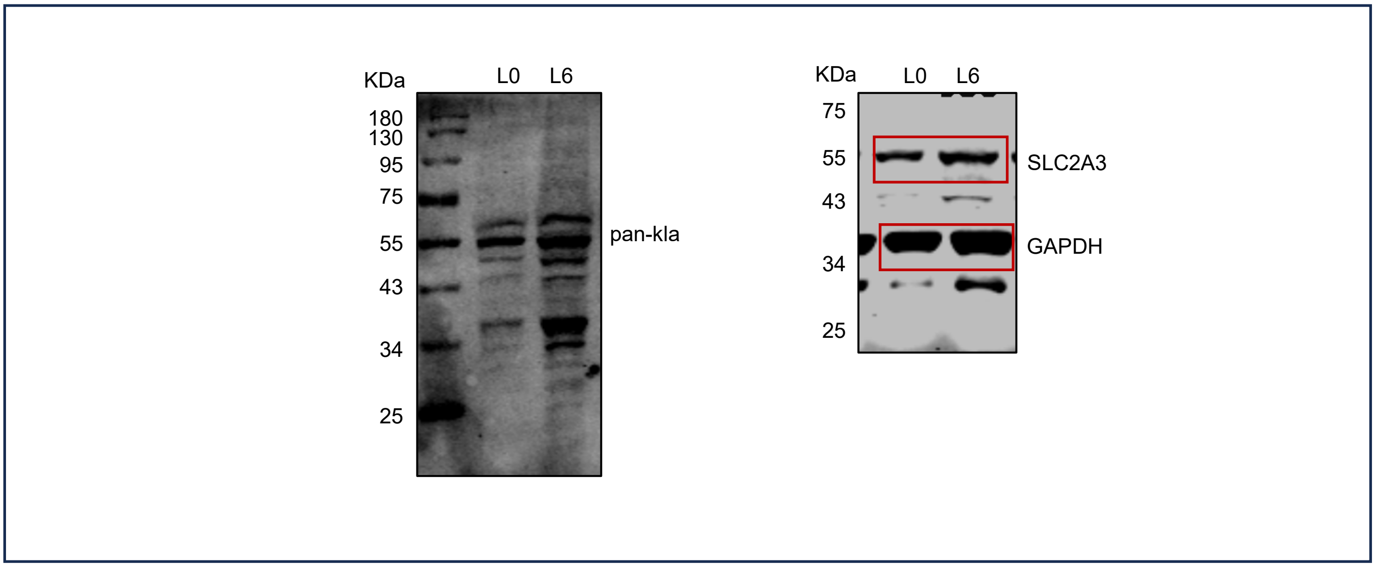

Full unedited gels for figure 3D

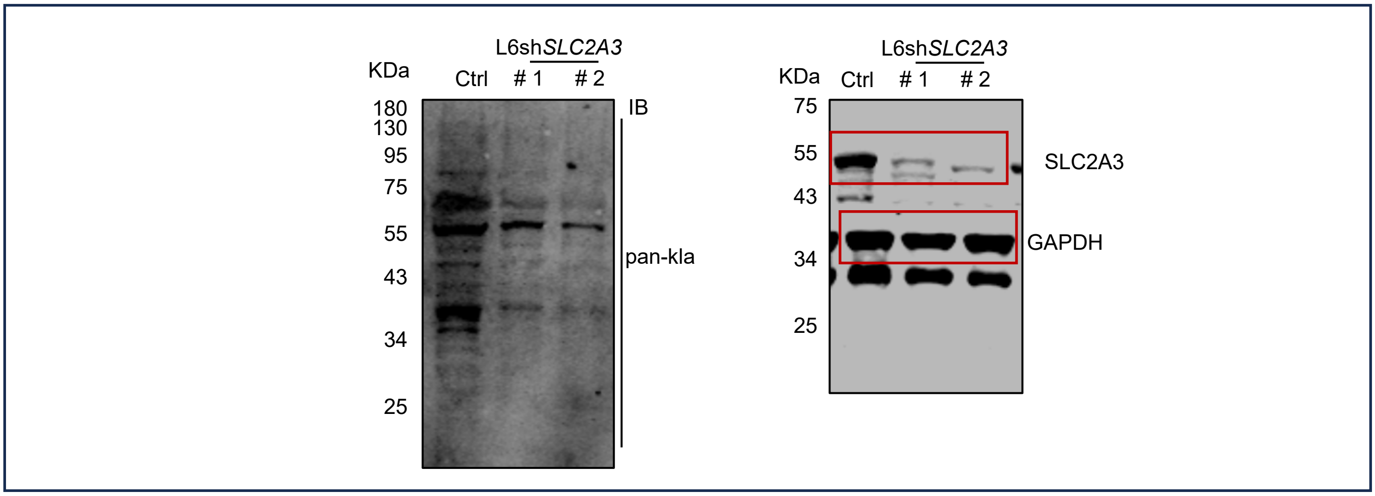

Full unedited gels for figure 3H

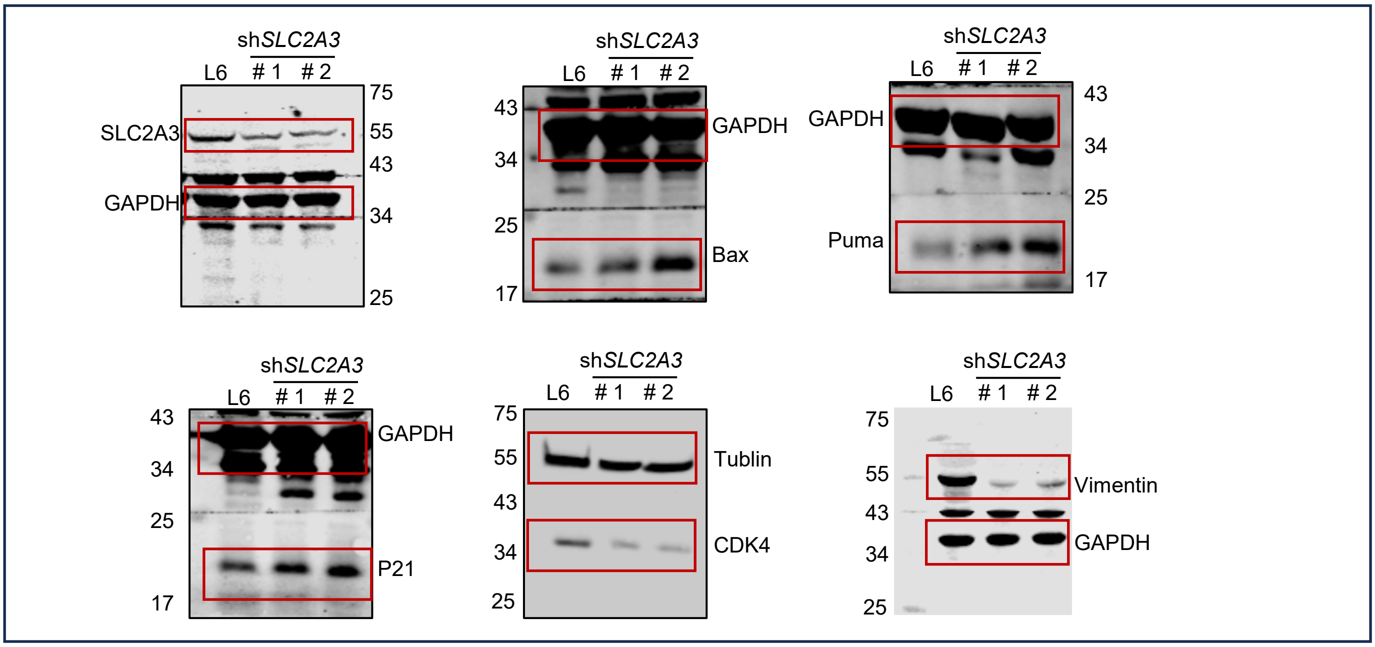

Original blot images

Full unedited gels for figure 3I

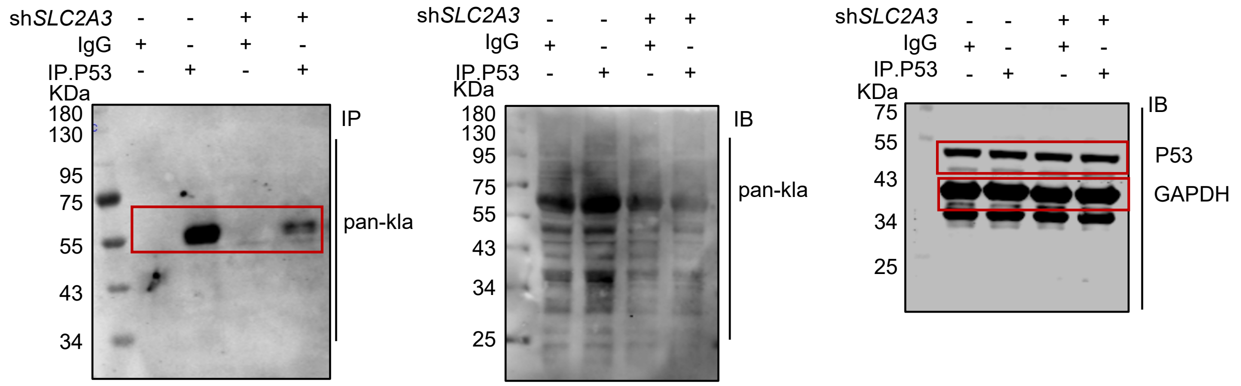

Full unedited gels for figure 3J

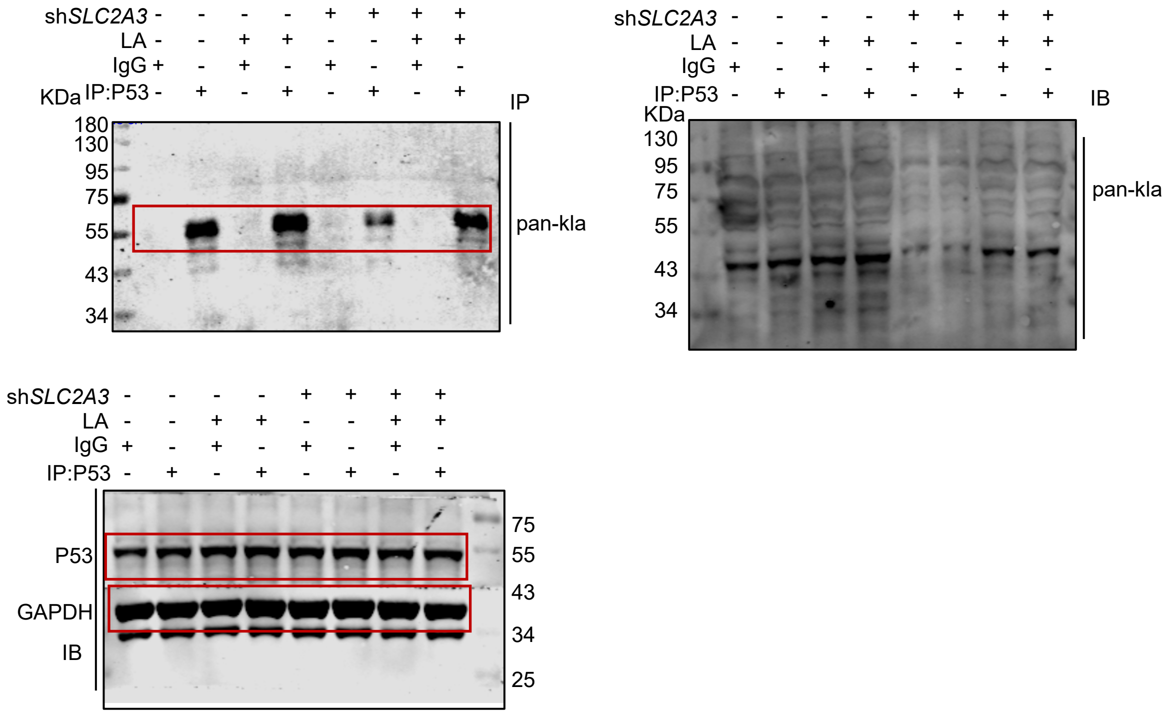

Original blot images

Full unedited gels for figure 3K

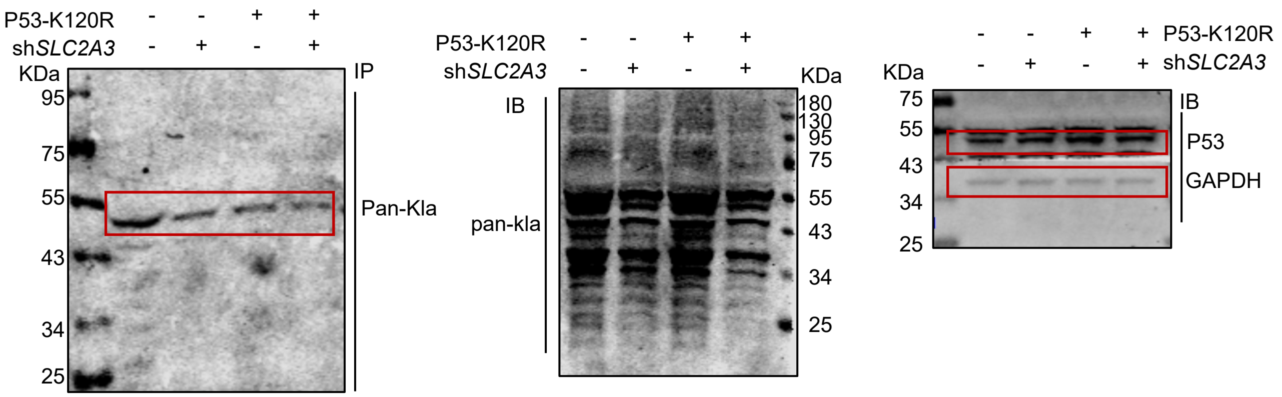

Full unedited gels for figure 3L

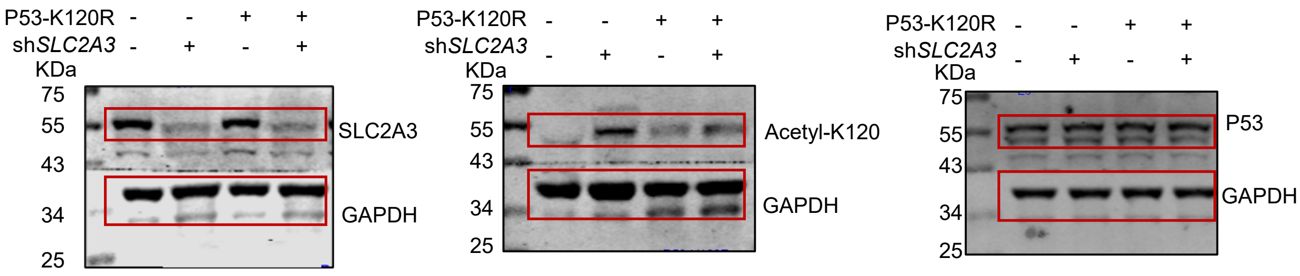

Full unedited gels for figure 3N

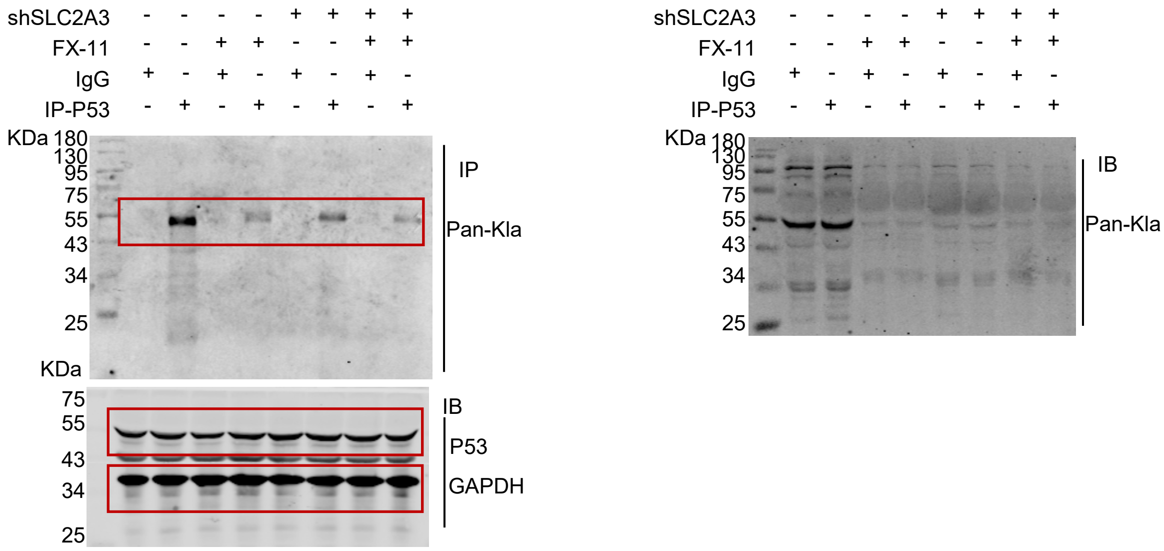

Original blot images

Full unedited gels for figure 5Q

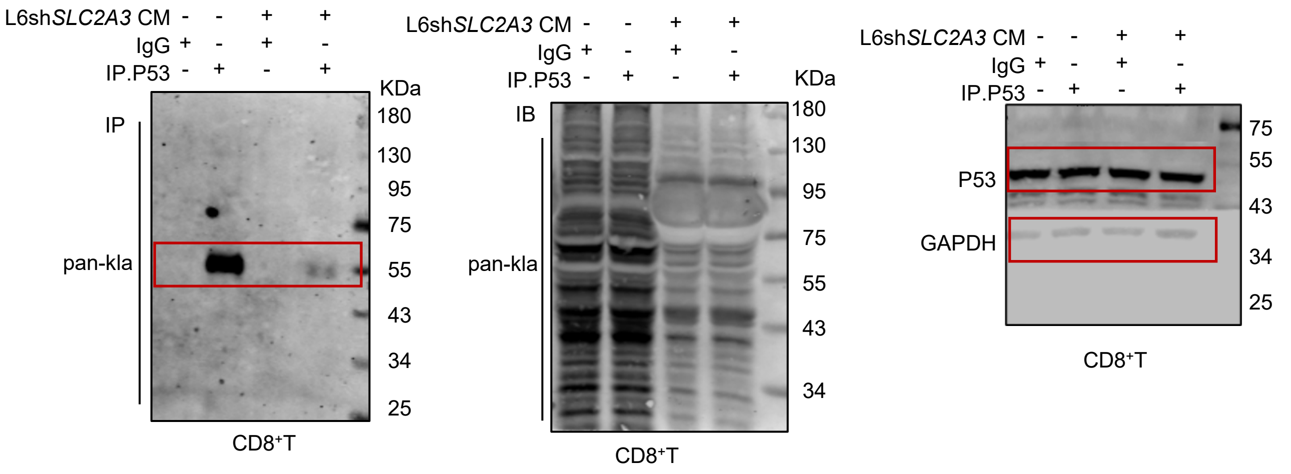

Full unedited gel for figure 5R

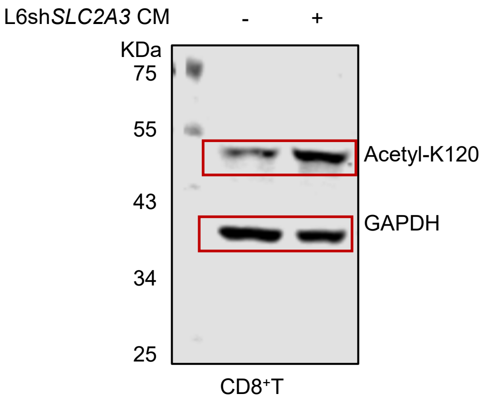

Original blot images

Full unedited gels for figure 5U

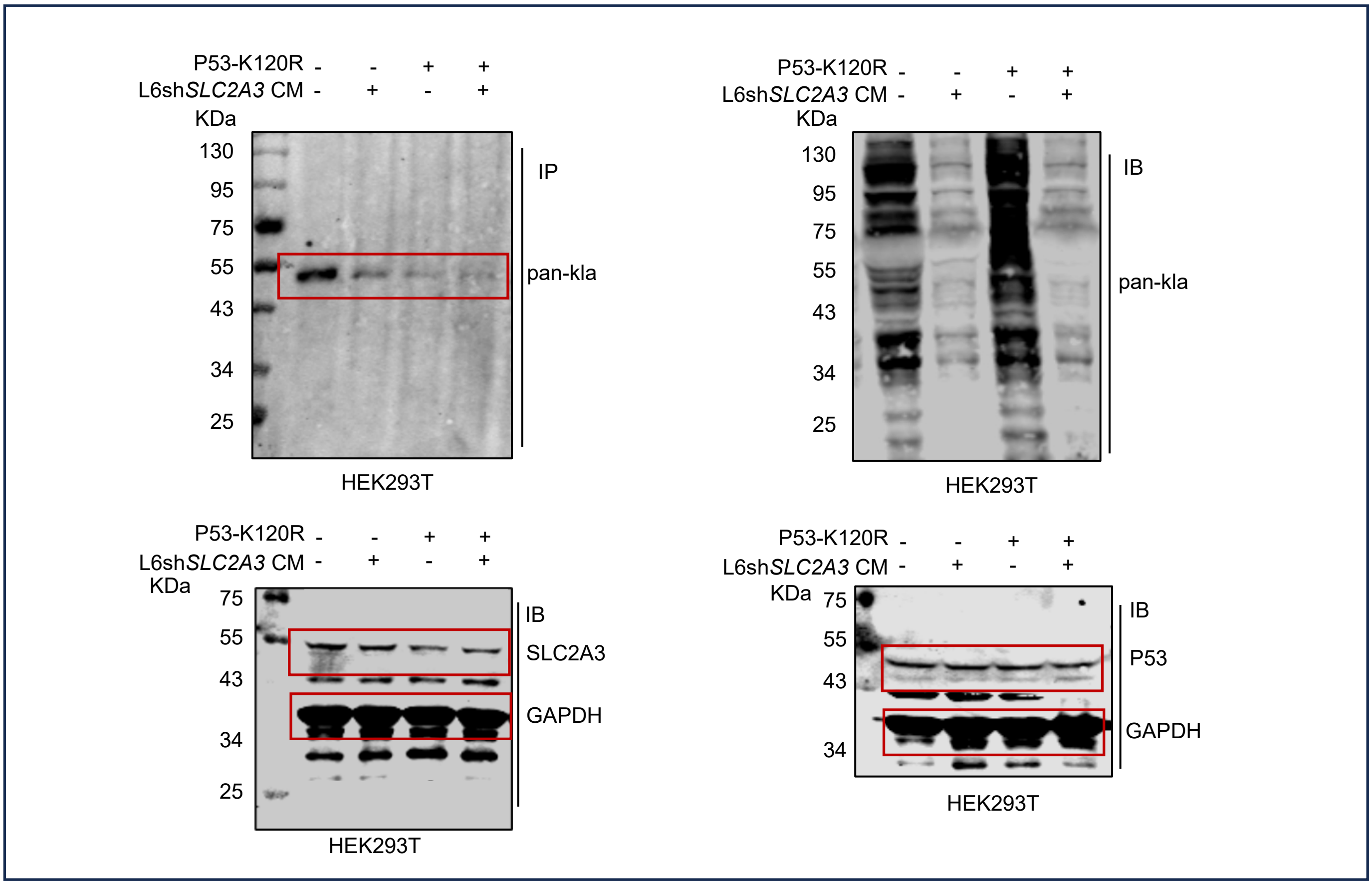

Full unedited gels for figure 5V

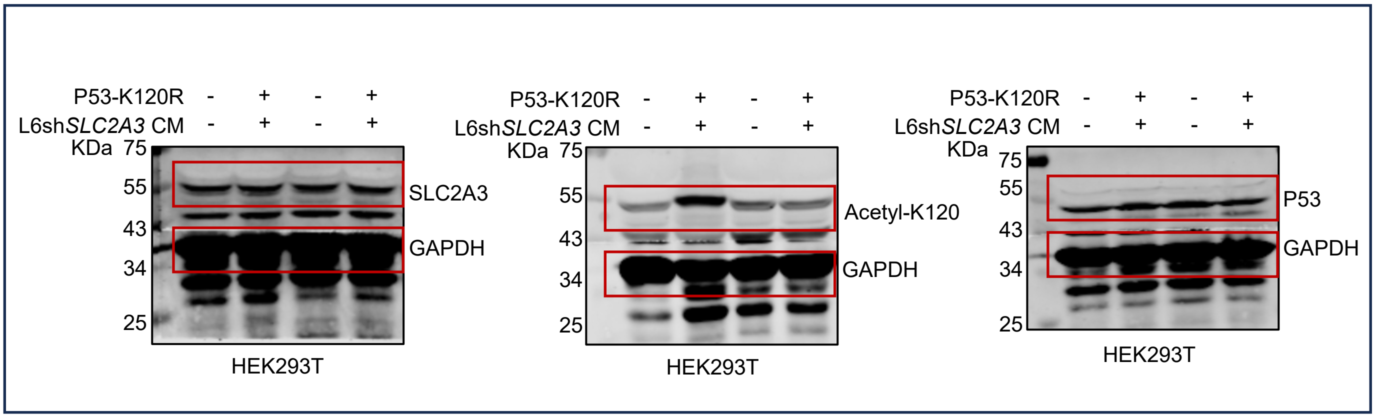

Full unedited gel for figure 6B

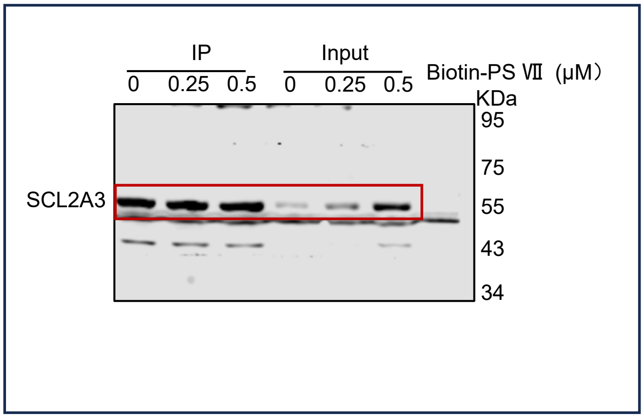

Full unedited gel for figure 6C

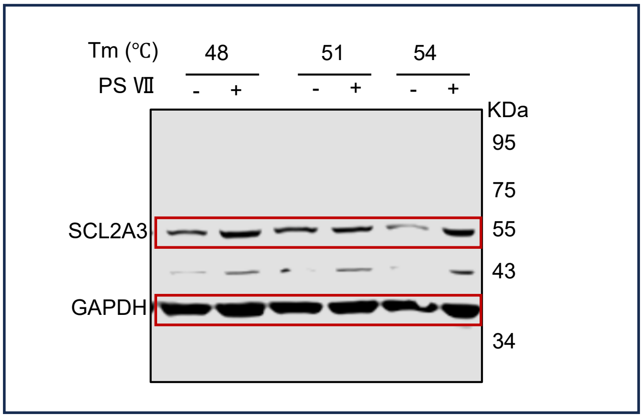

Original blot images

Full unedited gel for figure 6D

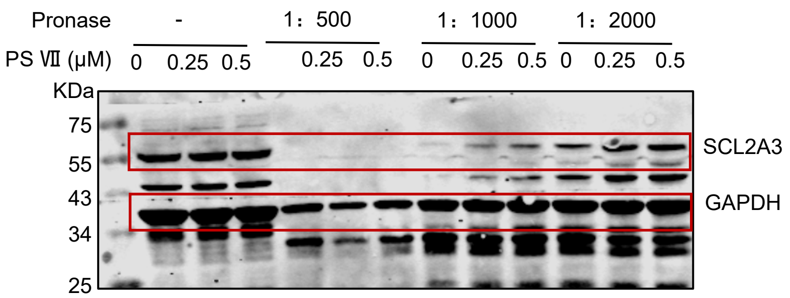

Full unedited gel for figure 6H

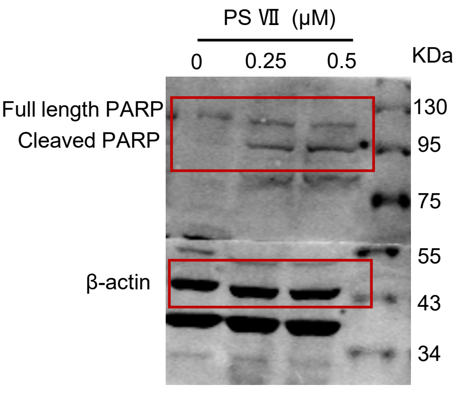

Full unedited gels for figure 6J

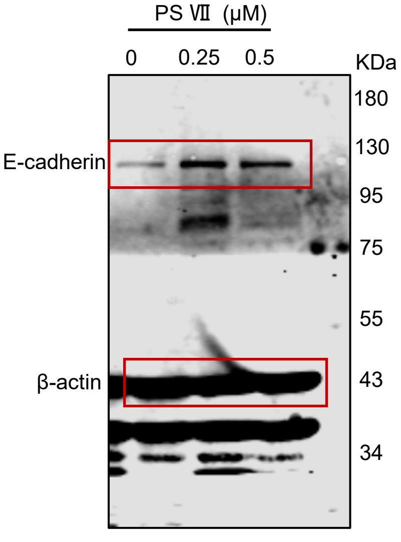

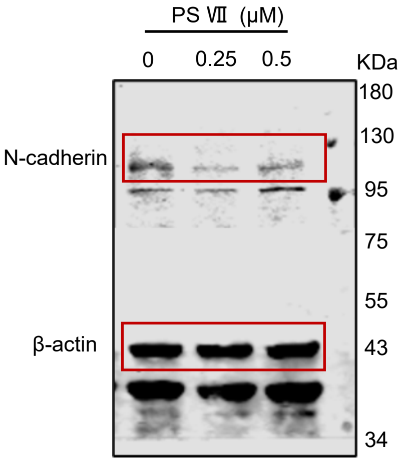

Original blot images

Full unedited gels for figure 7O

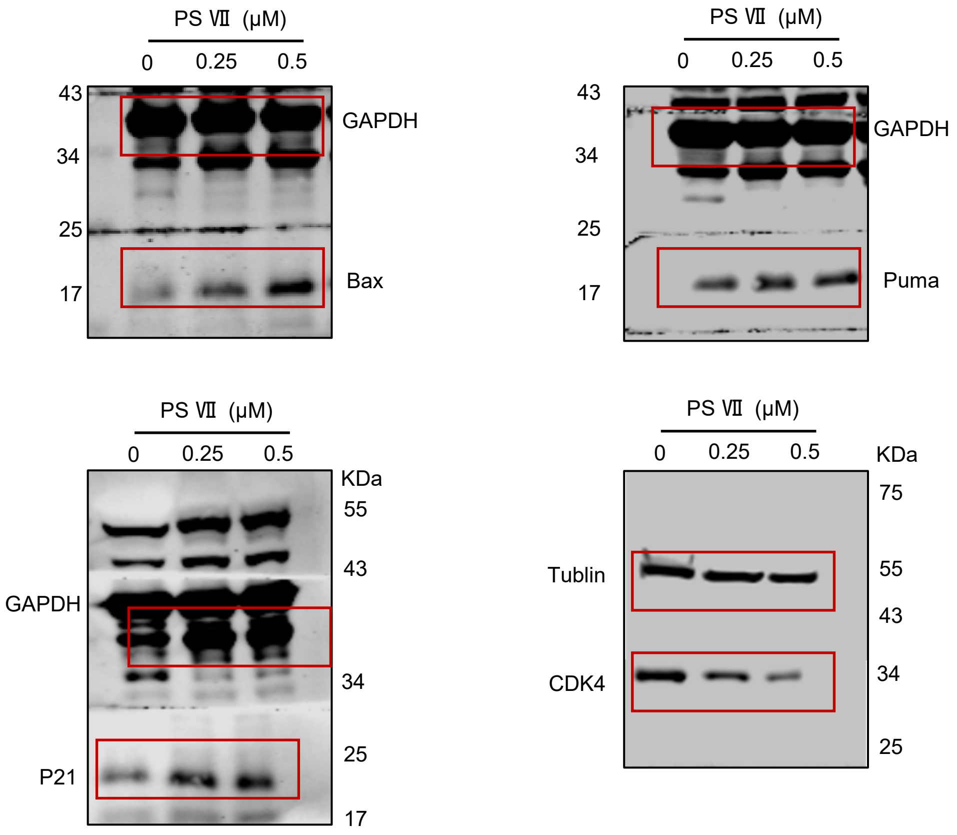

Full unedited gels for figure 7P

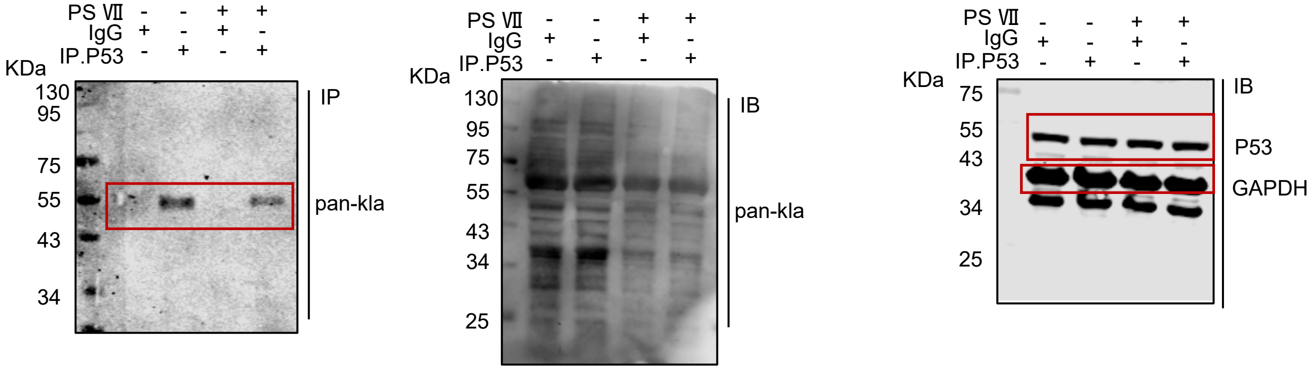

Original blot images

Full unedited gels for figure S1P

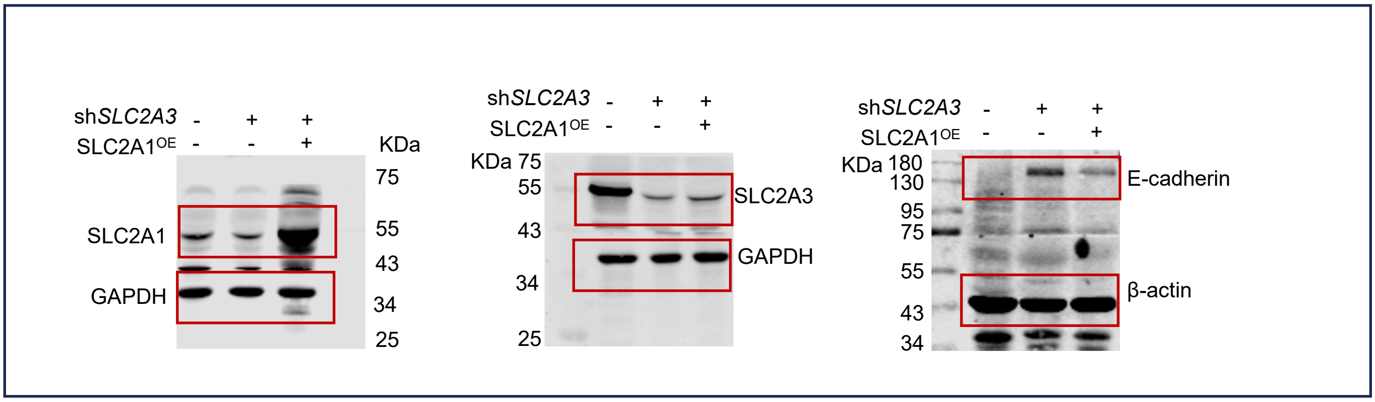

Full unedited gels for figure S1Q

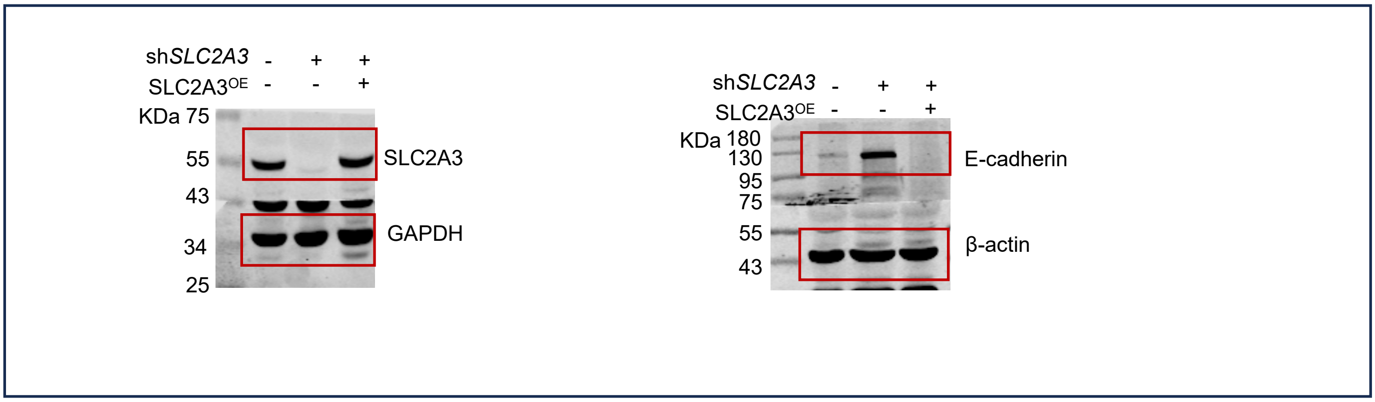

Full unedited gel for figure S3I

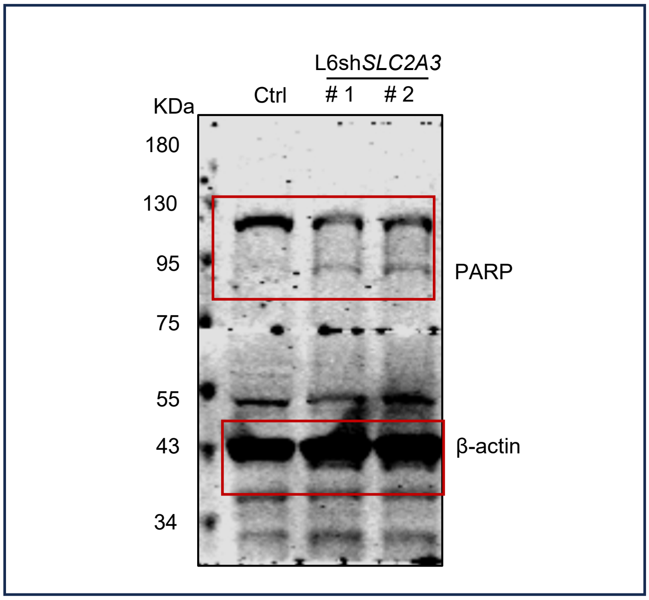

Full unedited gel for figure S3J

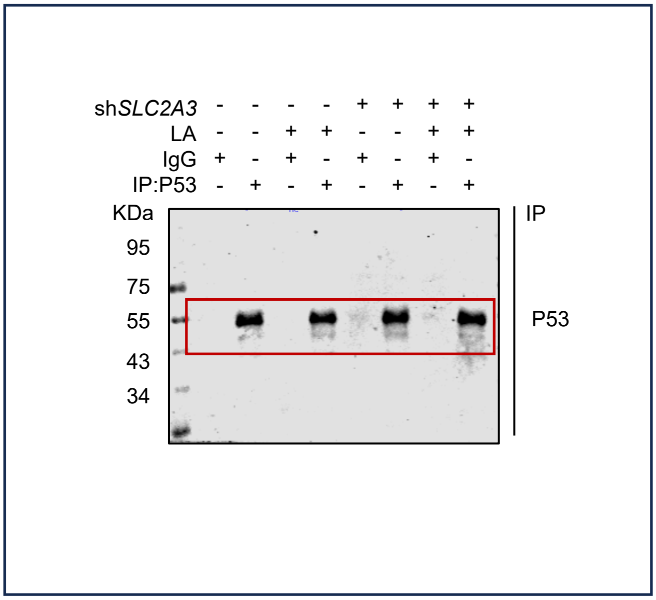

Original blot images

Full unedited gel for figure S3K

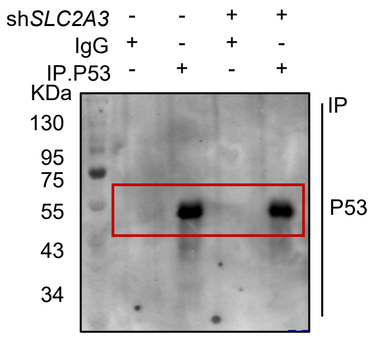

Full unedited gel for figure S3L

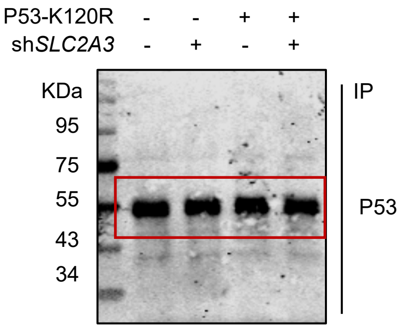

Full unedited gel for figure S4H

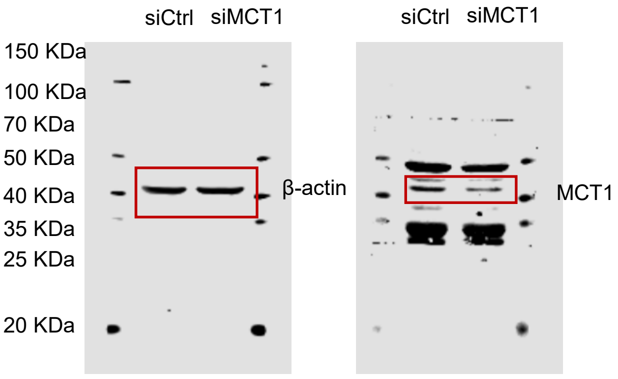

Full unedited gel for figure S4I

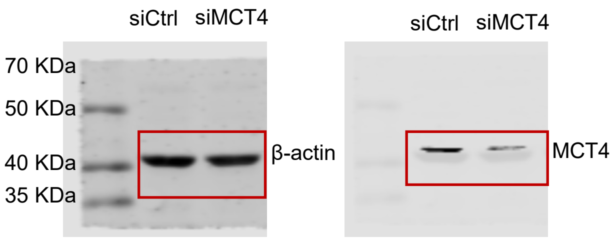

Original blot images

Full unedited gels for figure S4O

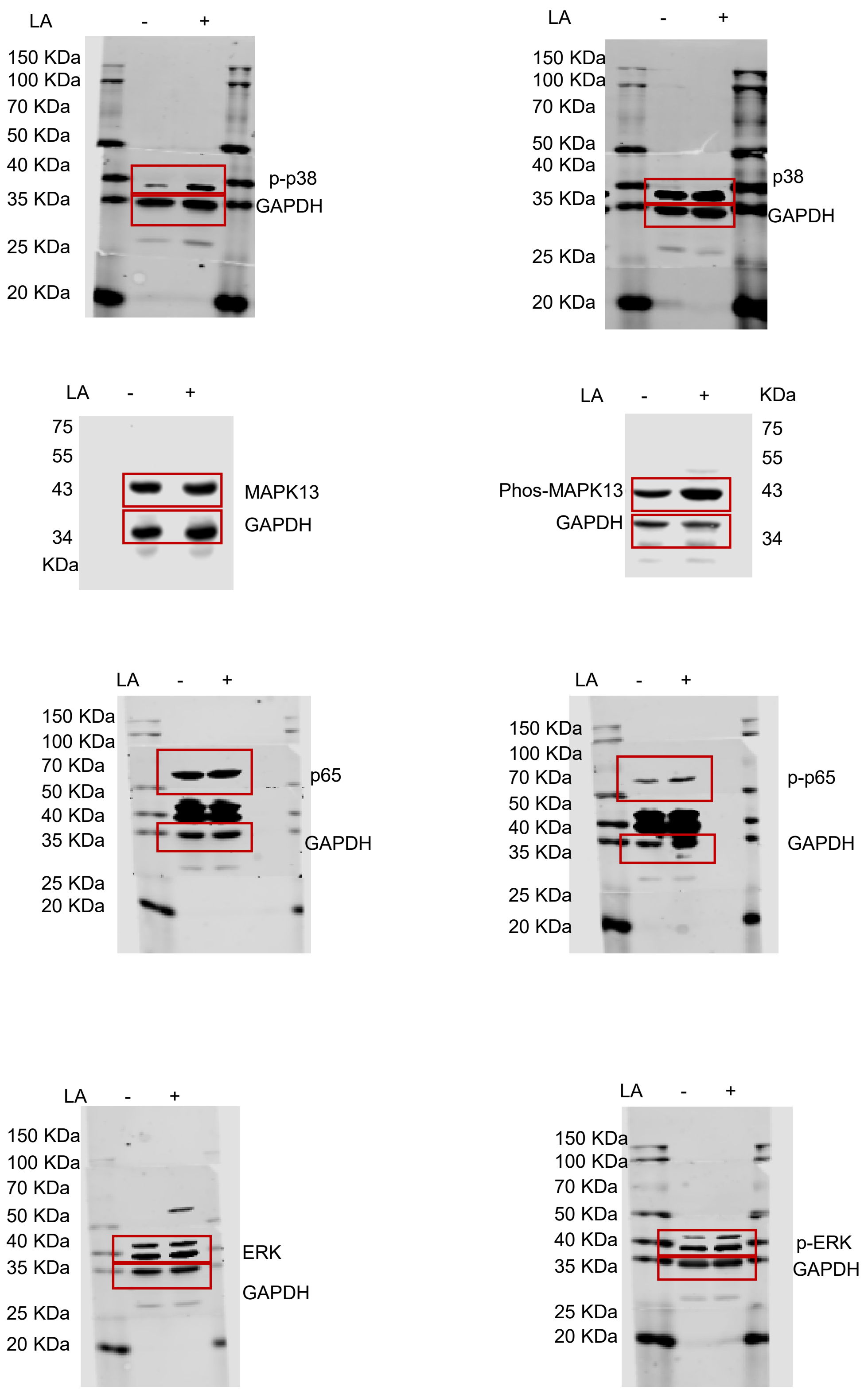

Original blot images

Full unedited gel for figure S5I

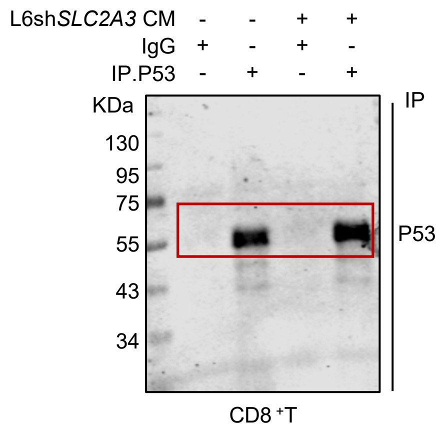

Full unedited gel for figure S5J

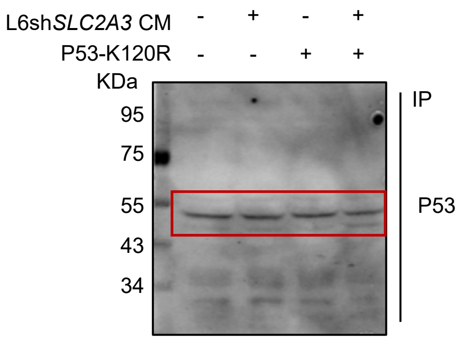

Full unedited gel for figure S6A

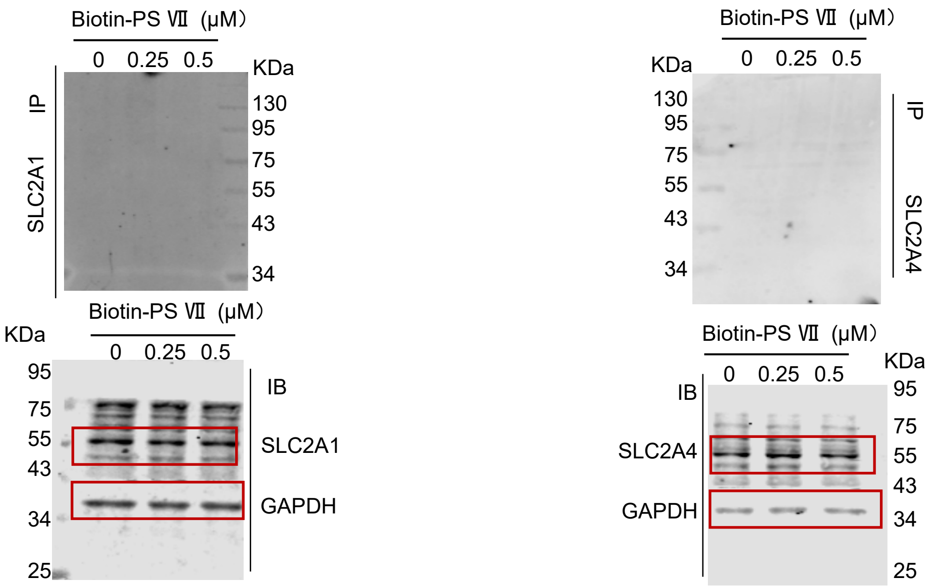

Full unedited gel for figure S6E

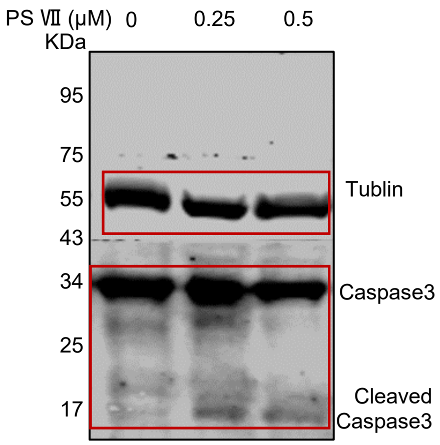

Full unedited gel for figure S7H

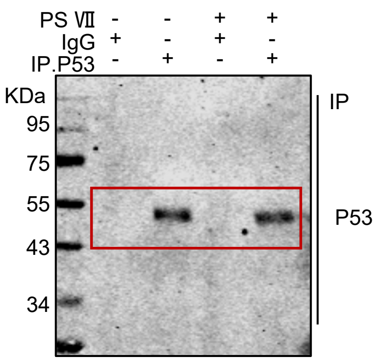

Supplement: Supplementary file 2 — Supporting File 2: advs74072‐sup‐0002‐SuppMat.pdf. [file ADVS-13-e16622-s001.pdf]
